# Rapid spontaneous generation of organosulfur from inorganic sulfur in atmospheric microdroplets

**DOI:** 10.1038/s41467-026-77473-5

**Published:** 2026-09-10

**Authors:** Huixia Han, Dongmei Zhang, Zegang Dong, Jiaqi Wang, Shuang Chen, Junjun Deng, Libin Wu, Wei Hu, Mingjin Tang, Bo Long, Jialei Zhu, Cong-Qiang Liu, Pingqing Fu

**Affiliations:** 1 Institute of Surface-Earth System Science, School of Earth System Science, Tianjin University, Tianjin, China; 2 School of Materials Science and Engineering, Guizhou Minzu University, Guiyang, China

**Keywords:** Atmospheric chemistry, Environmental chemistry

## Abstract

Organosulfur compounds are important constituents of atmospheric aerosols and have been extensively investigated through field observations, laboratory experiments, and atmospheric modeling. However, the mechanisms underlying their formation and atmospheric abundance remain incompletely understood. Ubiquitous in the atmosphere, micrometer-sized droplets provide unique interfacial reaction environments that may facilitate organosulfur formation. Here, we show that sulfite-derived inorganic sulfur species react with oxygenated volatile organic compounds in microdroplets to generate organosulfur compounds under catalyst-free conditions without external energy input. Theoretical calculations suggest that interfacial electric fields facilitate organosulfur formation by lowering reaction activation barriers. Furthermore, several organosulfur species identified in laboratory experiments were also detected in ambient aerosols collected from an urban site and a high-altitude mountain station, supporting the potential atmospheric relevance of this pathway. Our findings provide insights into the role of microdroplet interface in organosulfur formation and their potential contribution to atmospheric aerosol chemistry.

## Abstract (web summary)

Atmospheric microdroplets facilitate organosulfur formation from inorganic sulfur and oxygenated volatile organic compounds, revealing their potential role in atmospheric aerosol chemistry.

## Introduction

Organosulfur compounds play a crucial role in radiative forcing, global climate regulation and human health owing to their hygroscopicity, optical properties, and toxicity^1–3^. Traditionally known organosulfur formation mechanisms include the reactive uptake of epoxides^4,5^, sulfate esterification reactions^6,7^, heterogeneous reactions of SO_2_^8,9^, and radical-initiated processes that typically require transition metal ions (TMI) catalysis (e.g., Fe^3+^, Mn^2+^) or additional radiation in bulk aqueous solutions^10–12^. However, estimates of organosulfur concentrations remain subject to large uncertainty^13^. These discrepancies highlight a critical gap in understanding the formation mechanisms of organosulfur under atmospheric conditions.

Field observations consistently show strong correlations between organosulfur and inorganic sulfur species^2^, implying that their transformation is likely coupled in the atmosphere. In inorganic sulfur chemistry, the oxidation of S(IV) (SO_3_^2-^, HSO_3_^-^, and/or SO_2_•H_2_O) to S(VI) (SO_4_^2-^) is a fundamental atmospheric process that underpins sulfate aerosol formation, haze evolution, and acid rain^14,15^. Conventionally, sulfate formation has been primarily attributed to homogeneous oxidation of S(IV), either in the gas phase or in the bulk aqueous phase, through reactions with oxidants such as •OH, H_2_O_2_, O_3_, HONO, or NO_2_^16^. Despite extensive studies on the formation mechanisms of sulfate, atmospheric models consistently underestimate sulfate concentrations during haze events, revealing substantial gaps in our understanding of S(IV) oxidation pathways^17–19^. The observed sulfate deficit correlates strongly with aerosol liquid water content^19^, suggesting that heterogeneous or interfacial S(IV) oxidation processes may play a crucial role in polluted atmospheric environments. However, current studies on sulfur oxidation occurring at the interface remain largely focused on inorganic sulfur species^20–22^. In contrast, organosulfur formation has received limited attention and has mainly been investigated in a few specific organosulfur systems, such as dimethyl sulfide, thiols, and thioethers^23–25^. Consequently, the broader conversion of inorganic sulfur into organosulfur compounds remains largely unexplored.

Recently, microdroplet chemistry has gained prominence as a transformative approach due to the fact that chemical reactions within microdroplets may be accelerated by up to 6 orders of magnitude relative to those in bulk water, which can even trigger some reactions that cannot occur in bulk solution^26–28^. This remarkable acceleration has been ascribed to the distinct physicochemical environment of microdroplets, including extreme pH, partial solvation, enrichment and orientation of the reagents, and ultrahigh electric field on the order of 10^9 ^V·m^−1^^29–34^. The origin of such intense electric field has been attributed to electric double layers, alignment of the interfacial O–H bonds, or the partial charge separation yielding H_2_O^+^-H_2_O^-^ pairs^32–35^. The interfacial electric field strength is sufficiently strong to extract electrons from hydroxide ions (OH^-^), generating hydroxyl radicals (•OH) and solvated electrons^36,37^, which can subsequently initiate oxidation and reduction reactions^38–41^. These findings highlight that microdroplets can serve as active microreactors in which unique interfacial physicochemical properties govern reaction dynamics, offering valuable insights into chemical transformations occurring within the ubiquitous atmospheric micrometer-sized aqueous droplets, such as cloud (10–20 µm), fog ( ~ 10 µm), and wet aerosols (0.002–10 µm)^42–44^.

Given the variable valence states of sulfur and the spontaneous redox capability of microdroplets, it is plausible that inorganic S(IV) could generate reactive sulfur species at the air-water interface of microdroplets and subsequently participate in the formation of organosulfur compounds. In this study, we used sulfite (SO_3_^2-^) and several representative oxygenated volatile organic compounds (OVOCs) as substrates to elucidate potential organosulfur compounds formation pathways in water microdroplets. Notably, a variety of organosulfur compounds rapidly formed within hundreds of microseconds and subsequently detected using high-resolution mass spectrometry. Mechanistically, the strong interfacial electric field of water microdroplets drives the oxidation of SO_3_^2-^ to SO_3_^-•^, which then adds to unsaturated C=C bond. Meanwhile, HSO_3_^-^ undergoes nucleophilic addition to carbonyl and epoxide groups. Collectively, these processes enable the interfacial conversion of inorganic sulfur into organosulfur compounds. Quantum chemical calculations and metadynamics simulations were further employed to elucidate the mechanisms of organosulfur formation at the air-water interface of microdroplets. Furthermore, organosulfur products identified in these microdroplet experiments were detected in ambient aerosol samples collected from urban Tianjin and a high-mountain site at the Shanghuang Station in East China using Fourier transform ion cyclotron resonance mass spectrometry (FT-ICR MS). This work highlights the contribution of aerosol microdroplet interface to atmospheric organosulfur formation and secondary organic aerosol (SOA) chemistry.

## Results

### Spontaneous oxidation of inorganic sulfur

**Fig. 1:**
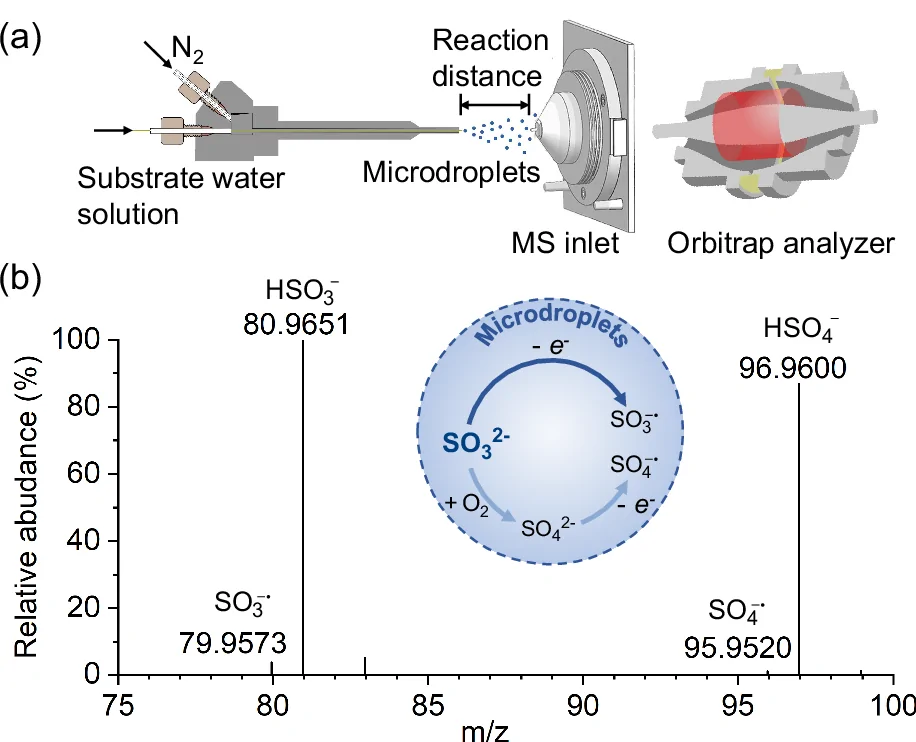
Spontaneous oxidation of SO_3_^2-^ at the air-water interface of microdroplets. **a** Schematic illustration of the experimental setup. **b** Mass spectrum of aqueous Na_2_SO_3_ solution (40 µM) nebulized into microdroplets obtained by a LTQ Orbitrap Velos. Mass spectrometry (MS), mass-to-charge ratio (*m*/*z*).

To probe the behavior of SO_3_^2-^ at the microdroplet interface, an aqueous solution of Na_2_SO_3_ (40 µM) was prepared, representative of atmospherically relevant aqueous-phase S(IV) concentrations^19^. The solution was then nebulized using high-pressure N_2_ (100 psi) and introduced into the heated inlet of a mass spectrometer (LTQ Orbitrap Velos) operated in negative ion mode (Fig. 1a). The high-pressure N_2_ exerts strong aerodynamic shear on the liquid stream, breaking it into microdroplets through pneumatic atomization. The mass spectrum (Fig. 1b) shows that the dominant peak at mass-to-charge ratio (*m*/*z*) 80.9651 corresponds to HSO_3_^-^, which is attributed to the ionization of SO_3_^2-^ in the presence of water. In addition, a distinct signal at *m*/*z* 79.9573 was identified as SO_3_^-•^, which demonstrates that SO_3_^2-^ can undergo rapid and spontaneous oxidation involving one-electron loss. This oxidation is likely facilitated by the strong interfacial electric field in microdroplets and occurs without catalysts, external potential or radiation. Furthermore, a pronounced signal at *m*/*z* 96.9600 (HSO_4_^-^) indicates efficient transformation of S(IV) to S(VI) through the oxidation reaction between SO_3_^2-^ and O_2_. Similarly, the weak peak at *m*/*z* 95.9520, corresponding to SO_4_^-•^, is generated from SO_4_^2-^ through one-electron loss.

The oxidation of SO_3_^2-^ to SO_3_^-•^ could potentially arise from several other factors, including dissolved oxygen, trace transition metals in solution, or ionization-induced processes in the mass spectrometer. To evaluate these possibilities, a series of control experiments were conducted. Reducing the dissolved oxygen concentration from 7.07 to 2.43 mg·L^−1^ by boiling ultrapure water had no significant effect on the SO_3_^-•^ signal intensity. Additionally, inductively coupled plasma tandem mass spectrometry (ICP-MS) analysis showed that trace metal concentrations in ultrapure, deionized, and distilled water were all below 0.1 ppb (Table S1), and no significant differences in SO_3_^-•^ production were observed among microdroplet experiments performed with these different water matrices. Furthermore, SO_3_^-•^ was not detected when a Na_2_SO_3_ solution was analyzed using the same mass spectrometer operated with a conventional electrospray ionization (ESI) source, and no external voltage was applied during the microdroplet experiments. Taken together, these results rule out dissolved oxygen, trace transition metals, and ionization-induced processes as major contributors to SO_3_^-•^ formation.

To further investigate the role of the interfacial electric field in SO_3_^-•^ formation, pressure-dependent experiments were conducted. Previous studies have shown that increasing the sheath gas pressure from 40 to 120 psi reduces droplet diameter from approximately 20 to 4 μm, thereby enhancing the interfacial electric field due to increased surface curvature^32,39^. As shown in Fig. S2, the SO_3_^-•^ signal intensity increased with increasing sheath gas pressure. This trend is consistent with a contribution from enhanced interfacial electric field in smaller microdroplets, which may facilitate electron transfer processes involved in SO_3_^-•^ generation. Nevertheless, enhanced SO_3_^-•^ abundance may arise from the combined effects of the interfacial electric field and other physicochemical characteristics inherent to microdroplet environments, including evaporation-driven concentration effects, reactant enrichment, altered solvation environments, and nonequilibrium interfacial dynamics^45,46^.

### Conversion of inorganic sulfur to organosulfur

With the generation of SO_3_^-•^ and SO_4_^-•^ at the microdroplet interface, we explored the reactions between representative OVOC precursors and Na_2_SO_3_. Several representative biogenic and anthropogenic OVOCs, including glyoxal, alpha-pinene oxide, methacrolein, and 3-methyl-2-butenal, were selected as substrates. The resulting spray of microdroplets, an aqueous mixture of OVOC substrates and Na_2_SO_3_ forced through a fused silica capillary by a syringe pump under high-pressure N_2_ (100 psi), was directed toward the inlet of LTQ Orbitrap Velos for real-time analysis. Because the reaction effectively ceases upon entering the heated inlet of the mass spectrometer, the flight time of the microdroplets between the nebulizer outlet and the inlet is taken as the reaction time^47^. Under these conditions, the size of the microdroplets is approximately 5 μm, and speed is about 90 m/s^48,49^. These parameters were not directly measured in this work and should therefore be considered approximate. Given a reaction distance of 20 mm, the corresponding reaction time is inferred to be 220 μs, which indicates that the reactions occur on an ultrafast timescale.

**Fig. 2:**
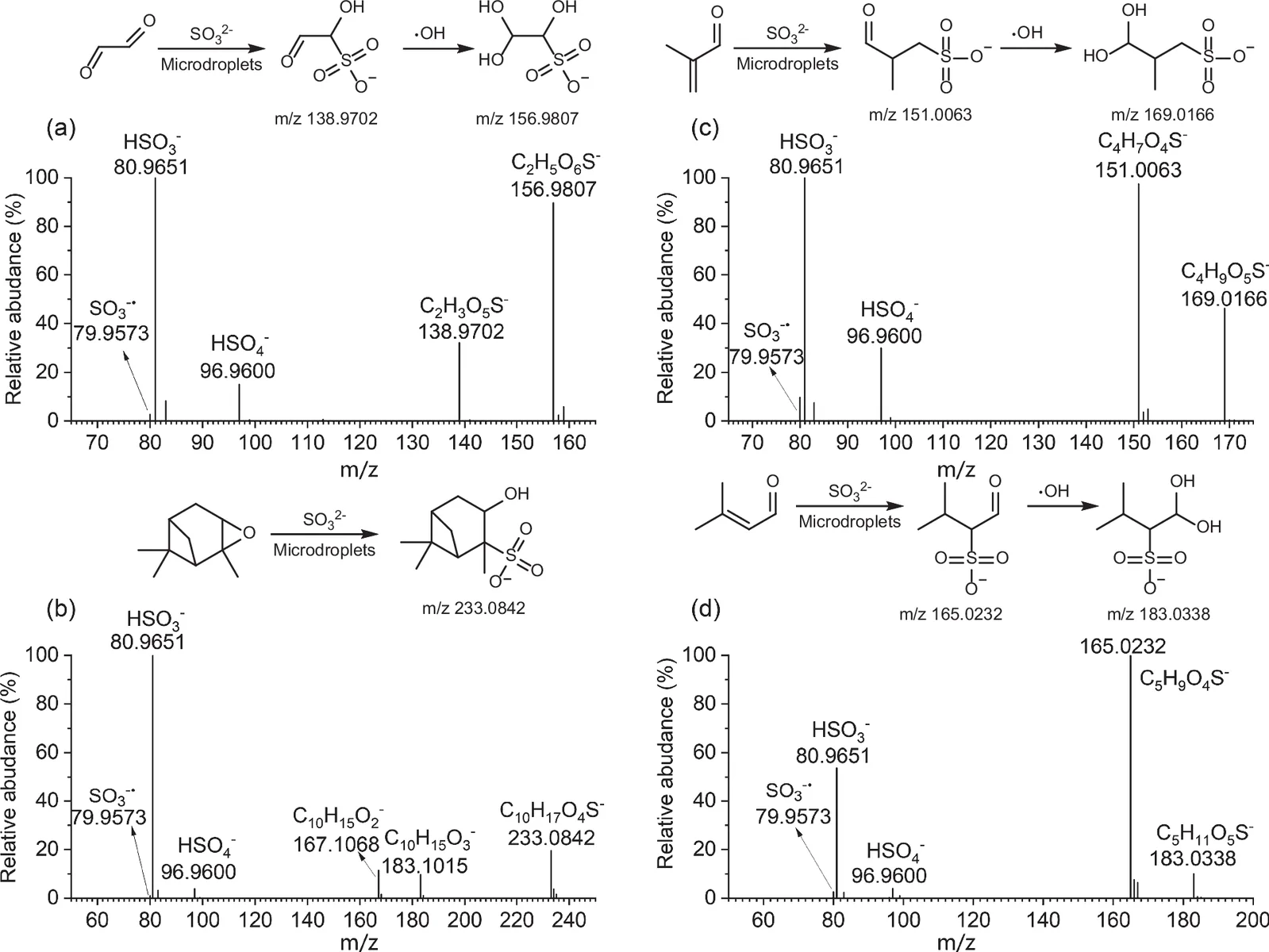
Typical mass spectra of a mixed solution of Na_2_SO_3_ and four representative oxygenated volatile organic compounds (OVOCs) sprayed into microdroplets obtained by LTQ Orbitrap Velos. **a** Na_2_SO_3_ (100 µM) and glyoxal (300 µM). **b** Na_2_SO_3_ (100 µM) and alpha-pinene oxide (700 µM). **c** Na_2_SO_3_ (100 µM) and methacrolein (100 µM). **d** Na_2_SO_3_ (100 µM) and 3-methyl-2-butenal (30 µM). Mass-to-charge ratio (*m*/*z*).

Figure 2 displays the mass spectra obtained from spraying a mixture of individual OVOC with Na_2_SO_3_. The peaks at *m*/*z* 79.9573 (SO_3_^-•^), *m*/*z* 80.9651 (HSO_3_^-^), and *m*/*z* 96.9600 (HSO_4_^-^) were consistently detected in all mass spectra, indicating the ubiquitous spontaneous oxidation of SO_3_^2-^ at the air-water interface of microdroplets. When spraying the mixed solution of Na_2_SO_3_ and glyoxal, a product peak at *m*/*z* 138.9702 was observed (Fig. 2a), which possibly results from the nucleophilic attack of HSO_3_^-^ on the carbonyl group to form C_2_H_3_O_5_S^-^. The structures of the detected products were confirmed by tandem mass spectrometry (MS/MS) using collision induced dissociation (CID). The MS/MS spectrum of *m*/*z* 138.9702 exhibits a fragment ion peak at *m*/*z* 80.9650 corresponding to HSO_3_^-^ (Fig. S3a), which further supports that the structure for C_2_H_3_O_5_S^-^ contains a sulfonate. Notably, an additional peak at *m*/*z* 156.9807 (C_2_H_5_O_6_S^-^) was detected, which can be attributed to the attack of •OH on the carbonyl functionality of the primary product (*m*/*z* 138.9702). Previous studies have confirmed that •OH can spontaneously arise at the air-water interface of water microdroplets under a strong electric field, attributing to an electron loss from OH^-^^37,50–54^. To further verify the generation of •OH in water microdroplets, we introduced 2,2,6,6-tetramethyl-1-piperidinyloxy (TEMPO), a widely used •OH scavenger^55,56^, into the sprayed solution. As expected, the capture product at *m*/*z* 174.1482 (TEMPOOH) was detected (Fig. S4), which is consistent with previous reports^55,56^. In addition, the MS/MS spectrum of *m*/*z* 156.9807 was identified, which displayed not only a characteristic fragment ion at *m*/*z* 80.9650 (HSO_3_^-^), but also a neutral loss of 18 Da (H_2_O) fragment from the parent ion (Fig. S3b), supporting the presence of both a sulfonate moiety and a hydroxyl functional group in the structure of C_2_H_5_O_6_S^-^. These results demonstrate that HSO_3_^-^ and carbonyl compounds can readily react to form sulfonate in water microdroplets.

The major product was observed at *m*/*z* 233.0842, corresponding to C_10_H_17_O_4_S^-^, when spraying the mixed solution of Na_2_SO_3_ and alpha-pinene oxide (Fig. 2b). Mechanistically, the air-water interface of microdroplets exhibits acidic properties due to the preferential accumulation of protons within the interfacial region^57,58^. Under such conditions, epoxide group of alpha-pinene oxide proceeds acid-catalyzed ring opening followed by subsequent nucleophilic addition with HSO_3_^-^^59,60^. Notably, the MS/MS spectrum of *m*/*z* 233.0842 shows a fragmentation peak at *m*/*z* 80.9650 (HSO_3_^-^) (Fig. S5). These results indicate the spontaneous formation of sulfonate species at the air-water interface of microdroplets. Sulfonate formation involves the addition of HSO_3_^-^ on the epoxide carbon to form a C–S bond and a neighboring hydroxyl group.

In addition, the product peaks at *m*/*z* 167.1068 and *m*/*z* 183.1015 were also observed in the mass spectrum (Fig. 2b). These products may result from the acid catalyzed isomerization reaction of alpha-pinene oxide to form campholenic aldehyde under acidic environment at the microdroplets interface (Fig. S6)^57,58^. This mechanism of acid catalyzed isomerization of alpha-pinene oxide is widely accepted in previous studies^57,58^. The peaks at *m*/*z* 167.1068 and *m*/*z* 183.1015 are identified as oxidation products of campholenic aldehyde, as indicated by the successive mass shifts of +16 and +32 Da, respectively. The product peak at *m*/*z* 167.1068, corresponding to C_10_H_15_O_2_^-^, is likely formed via •OH addition followed by hydrogen-atom abstraction. Subsequently, the C=C bond in C_10_H_15_O_2_^-^ can further react with •OH to form a hydroxyalkyl radical, which then reacts with ambient O_2_ to produce a hydroxyperoxy radical. This peroxy group may undergo intramolecular hydrogen abstraction, releasing an HO_2_ group and yielding an enol intermediate^61^. This enol product then undergoes the enol-keto tautomerization, to yield a more thermodynamically stable carbonyl compound, giving rise to the product at *m*/*z* 183.1015 (C_10_H_15_O_3_^-^). Detailed reaction mechanisms are illustrated in Fig. S7. The MS/MS spectrum of *m*/*z* 167.1068 shows a neutral loss of 44 Da, suggesting the presence of a carboxylic acid group in this product (Fig. S8a). Similarly, the MS/MS spectrum of *m*/*z* 183.1015 also exhibits a neutral loss of 44 Da, confirming that *m*/*z* 183.1015 is a further oxidation product derived from *m*/*z* 167.1068 (C_10_H_15_O_2_^-^) via •OH. More fragment information of *m*/*z* 167.1068 and *m*/*z* 183.1015 is shown in Fig. S8, which is consistent with proposed oxidation mechanisms above and campholenic aldehyde standard reagent (Figs. S7 and S9). This result indicates that sulfonate formed from the alpha-pinene oxide and SO_3_^2-^ may include structural isomers derived from in situ isomerization (Fig. S15).

A prominent product peak at *m*/*z* 151.0063 was detected when a mixed solution of Na_2_SO_3_ and methacrolein was sprayed (Fig. 2c). This product, assigned to C_4_H_7_O_4_S^-^, probably formed through the addition of SO_3_^-•^ to the C=C bond of methacrolein, followed by hydrogen transfer from the aqueous environment. This preference for the alkene over the carbonyl group is attributed to the more electron-rich π-system of the C=C bond, which offers a kinetically more accessible and thermodynamically favorable pathway for radical addition^62,63^. The MS/MS spectrum of *m*/*z* 151.0063 shows a fragment ion peak at *m*/*z* 80.9650 (HSO_3_^-^) (Fig. S10a), confirming the incorporation of the sulfonate moiety. These findings suggest that the C=C bond can serve as a highly reactive site for SO_3_^-•^ addition, enabling the spontaneous formation of stable organosulfur products at the air-water interface of microdroplets. Notably, in aqueous environments, SO_3_^-•^ is a highly transient intermediate with an effective lifetime in the sub-millisecond regime^64,65^, whereas its gas phase lifetime remains undefined because solvation is required for stabilization^66^. The transient nature of SO_3_^-•^ is consistent with its low MS signal intensity and rapid involvement in subsequent reactions, with the microsecond residence time of microdroplets providing a temporal window compatible with these radical-mediated transformations. In addition, another product peak was detected at *m*/*z* 169.0166 (C_4_H_9_O_5_S^-^) (Fig. 2c), which can be attributed to further oxidation of the primary product (*m*/*z* 151.0063) initiated by •OH. The MS/MS spectrum of *m*/*z* 169.0166 (C_4_H_9_O_5_S^-^) displays a characteristic fragment ion at *m*/*z* 80.9650 (HSO_3_^-^) and a neutral loss of 18 Da (H_2_O) fragment (Fig. S10b), indicating that C_4_H_9_O_5_S^-^ contains a sulfonate moiety and a hydroxyl functional group. Subsequently, a structurally similar OVOC, 3-methyl-2-butenal, was investigated to evaluate the generality of this reaction pathway. A prominent product at *m*/*z* 165.0232 (C_5_H_9_O_4_S^-^) was detected (Fig. 2d) when spraying a mixed solution of Na_2_SO_3_ and 3-methyl-2-butenal, corresponding to SO_3_^-•^ addition to the C=C bond. The MS/MS spectrum shows a characteristic fragment ion at *m*/*z* 80.9650 (HSO_3_^-^), supporting the formation of a sulfonate group (Fig. S11). A subsequent product at *m*/*z* 183.0338 (C_5_H_11_O_5_S^-^) is attributed to further •OH functionalization. Consistent with methacrolein, these results highlight an efficient pathway for converting inorganic sulfur into stable organosulfur compounds at the air-water interface of microdroplets.

To further evaluate the role of •OH in organosulfur formation, tert-butanol (TBA) was used as a •OH scavenger^67^. As shown in Fig. S12, the addition of 50 μM TBA led to substantial decreases in the intensity ratios of C_2_H_5_O_6_S^-^/C_2_H_3_O_5_S^-^, C_4_H_9_O_5_S^-^/C_4_H_7_O_4_S^-^, and C_5_H_11_O_5_S^-^/C_5_H_9_O_4_S^-^, which are derived from the reactions of Na_2_SO_3_ with glyoxal, methacrolein, and 3-methyl-2-butenal, respectively. These results indicate that •OH contributes to the formation of organosulfur products in water microdroplets. Notably, C_2_H_5_O_6_S^-^, C_4_H_9_O_5_S^-^, and C_5_H_11_O_5_S^-^ may also be produced through hydrolysis-related pathways.

For structural identification, microdroplets were collected for 1 h and subsequently characterized by attenuated total reflectance Fourier-transform infrared (ATR-FTIR) spectroscopy. As shown in Fig. S13, the characteristic strong absorption bands at 1193 and 1050 cm^−1^ are assigned to the antisymmetric and symmetric stretching vibrations of the sulfonate group, respectively^68^. In addition, the signal observed near 621 cm^−1^ is attributed to C–S–O stretching vibrations, and the broad absorption centered at approximately 3327 cm^−1^ is assigned to O–H stretching vibrations^69^. These spectral features are consistent with previous reports and provide complementary spectroscopic evidence for the formation of organosulfur compounds in microdroplets.

Furthermore, ultra-high performance liquid chromatography-mass spectrometry (UPLC-MS) was utilized to evaluate potential constitutional and positional isomers. As shown in the extracted ion chromatograms (EICs) (Fig. S14), *m*/*z* 138.9702, *m*/*z* 156.9807, *m*/*z* 151.0063, *m*/*z* 169.0166, *m*/*z* 165.0232, and *m*/*z* 183.0338 each exhibited a single chromatographic peak, indicating that no significant isomeric contributions were detected under the present chromatographic conditions. In contrast, the EIC of *m*/*z* 233.0842, generated from the reaction of Na_2_SO_3_ and alpha-pinene oxide, displayed three distinct chromatographic peaks at retention times of 4.861, 5.084, and 5.443 min, suggesting the possible presence of multiple constitutional and/or positional isomers. The proposed structures of *m*/*z* 233.0842 are shown in Fig. S15. Compounds 1 and 2 are tentatively assigned to sulfonate adducts formed through nucleophilic ring-opening of alpha-pinene oxide by HSO_3_^-^, whereas compound 3 is proposed to originate from nucleophilic addition of HSO_3_^-^ to C=O of campholenic aldehyde, a rearrangement product generated through acid-catalyzed isomerization of alpha-pinene oxide in microdroplets. However, without commercially available standards for these organosulfur products, assigning the exact stereochemical configuration to each specific chromatogram peak remains a limitation.

Moreover, semi-quantitative analysis was performed using hydroxymethanesulfonate (HMS) as an internal standard at different reaction distances and OVOC concentrations. By adjusting the reaction distance from 5 to 40 mm, the corresponding reaction time was estimated to range from 55 to 495 μs. A clear increase in product yield was observed with increasing distance (Fig. S16). The average yield of these products reached up to 50% when the reaction distance is 20 mm. These results indicate that organosulfur formation occurs during the microsecond timescale flight of microdroplets rather than within the mass spectrometer inlet region, which is consistent with previous studies^47,70^. In addition, concentration-dependent experiments revealed that organosulfur product yields decreased with increasing OVOC concentrations (Fig. S17). This trend may be attributed to interfacial effects, as lower bulk concentrations increase the relative fraction of reactants partitioned to the air-water interface of microdroplets^71^. Taken together, these observations highlight the important role of interfacial processes in organosulfur formation and suggest that reactions occurring in microdroplets can provide a rapid and efficient pathway for sulfur conversion under atmospherically relevant conditions.

### Mechanistic studies

To conclude, organosulfur compounds can spontaneously form at the air-water interface of microdroplets when Na_2_SO_3_ and OVOCs are nebulized together, without the need for catalysts, strong oxidants, elevated temperatures, irradiation, or electrochemical inputs. The interfacial chemistry is initiated by the removal of an electron from the SO_3_^2-^ to form SO_3_^-•^, which is energy-demanding and rate-limiting in bulk solution but becomes feasible in microdroplets. Bulk phase experiments were conducted for all OVOC systems using the same reactant concentrations as those employed in the microdroplet experiments. After 72 h, trace amounts of organosulfur products were observed only in the Na_2_SO_3_ and 3-methyl-2-butenal system, whereas no detectable products were observed in the other OVOC systems. Previous studies further show that organosulfur formation in bulk requires substantially higher concentrations (2 mM) and the presence of Fe^3+^, with reaction times of 12 h^10,72^. These comparisons suggest that organosulfur formation is significantly enhanced in microdroplets relative to bulk experiments, despite differences in experimental conditions, such as concentration, oxidant availability, and mixing environments.

This enhanced feasibility and kinetic acceleration may originate from the unique interfacial environment of microdroplets, including partial solvation of reactants and transition states, local pH variations, and strong electric fields. For example, at the air-water interface, partial solvation of reactants and transition states reduces the activation enthalpy^73^. Concurrently, restricted translational and rotational degrees of freedom at the interface lower the configurational entropy barrier, leading to a net decrease in activation free energy^74^. In addition, the strong interfacial electric field further facilitates electron transfer from SO_3_^2-^ and stabilizes charged and radical intermediates^75^.

**Fig. 3:**
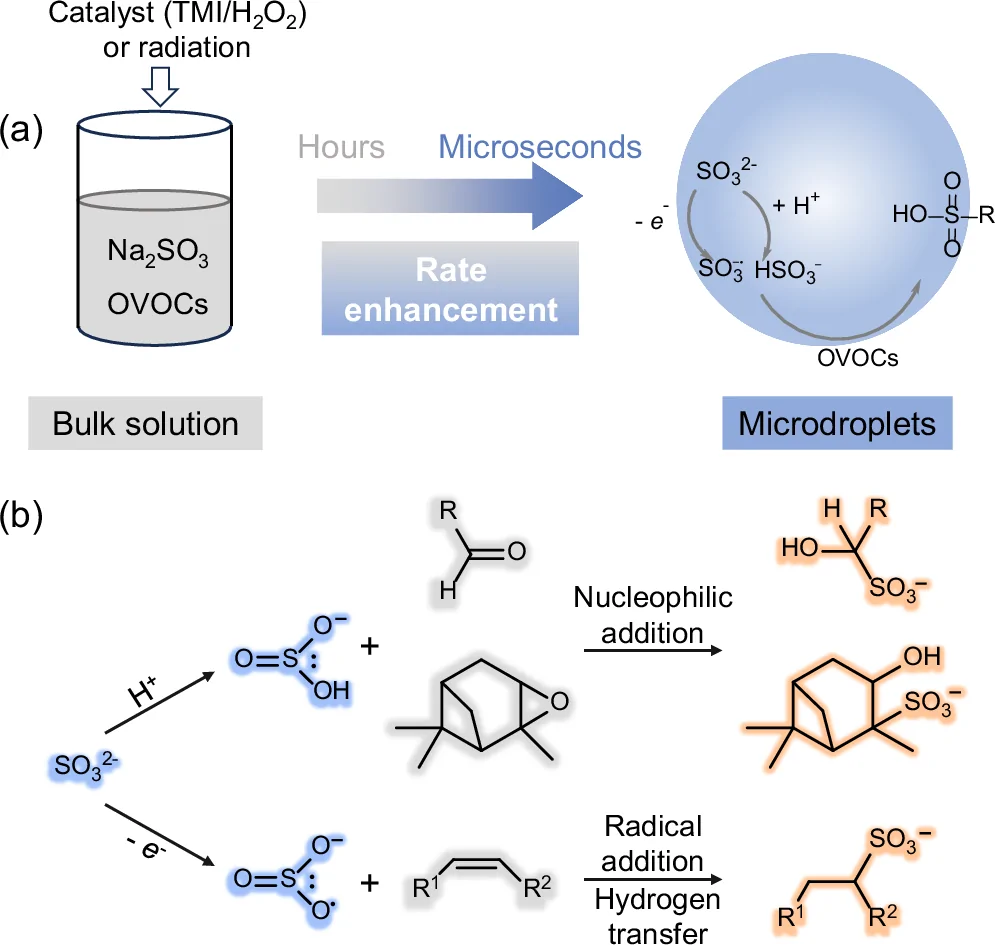
Interfacial mechanisms underlying organosulfur formation in microdroplets. **a** Schematic comparison of reactions between Na_2_SO_3_ and oxygenated volatile organic compounds (OVOCs) in bulk solution and microdroplets. **b** Proposed reaction mechanisms for organosulfur formation at the air-water interface of microdroplets. Transition metal ions (TMI).

The proposed reaction mechanisms between Na_2_SO_3_ and OVOCs involve both nucleophilic and radical pathways (Fig. 3b). Specifically, HSO_3_^-^ acts as a nucleophile toward the electrophilic carbon center in carbonyl groups and epoxide groups, leading to C–S bond formation through nucleophilic addition. The formation of SO_3_^-•^ triggers radical-mediated reactions with C=C bond of unsaturated OVOCs, followed by hydrogen transfer. In addition, it is known that the intense interfacial electric field of water microdroplets facilitates the spontaneous generation of •OH through electron transfer process from OH^-^, which provides oxidant that further modifies unsaturated functional groups (such as C=O bond) within the newly formed organosulfur species. Moreover, some OVOCs may undergo oxidation and isomerization in the distinct chemical environment at the air-water interface of microdroplets. This study reveals the fast and spontaneous transformation from inorganic sulfur to organosulfur in water microdroplets, providing a previously overlooked pathway for the generation of organosulfur compounds in atmospheric microdroplets.

**Fig. 4:**
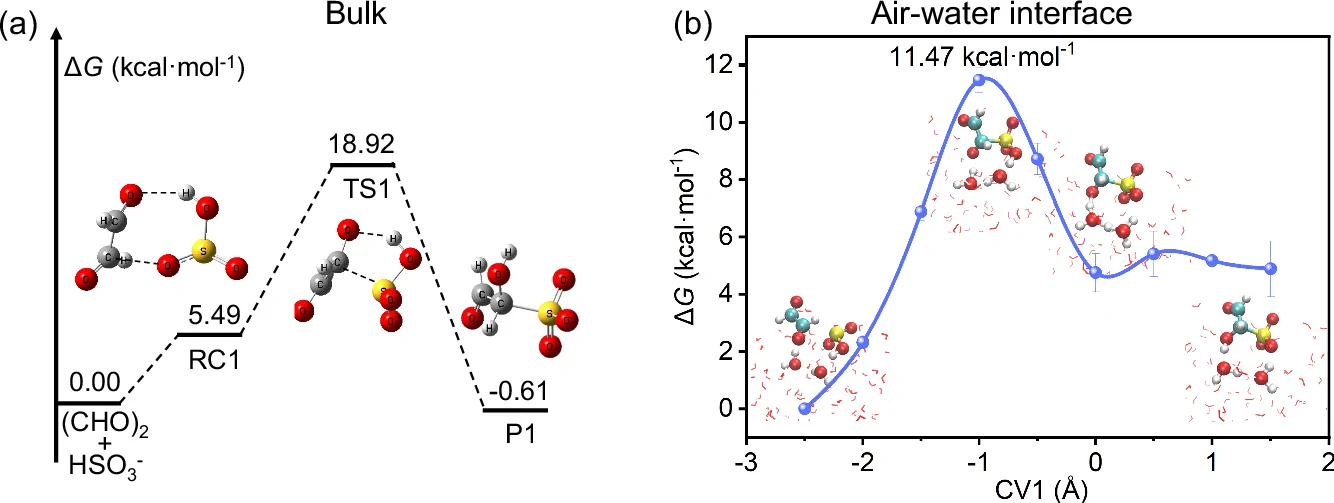
Microdroplet interface facilitates HSO_3_^-^ addition to glyoxal ((CHO)_2_) by lowering the reaction barrier. The calculated relative Gibbs free energy (Δ*G*) profiles for HSO_3_^-^ addition to glyoxal **a** in bulk solution and **b** at the air-water interface of microdroplets. Free energy profiles were obtained using thermodynamic integration. Error bars represent the standard error of the mean (SEM) estimated by block averaging time-series data from a single molecular dynamics (MD) trajectory at each *λ* value. Pre-reactive complex (RC), transition state (TS), product (P), collective variable (CV).

**Fig. 5:**
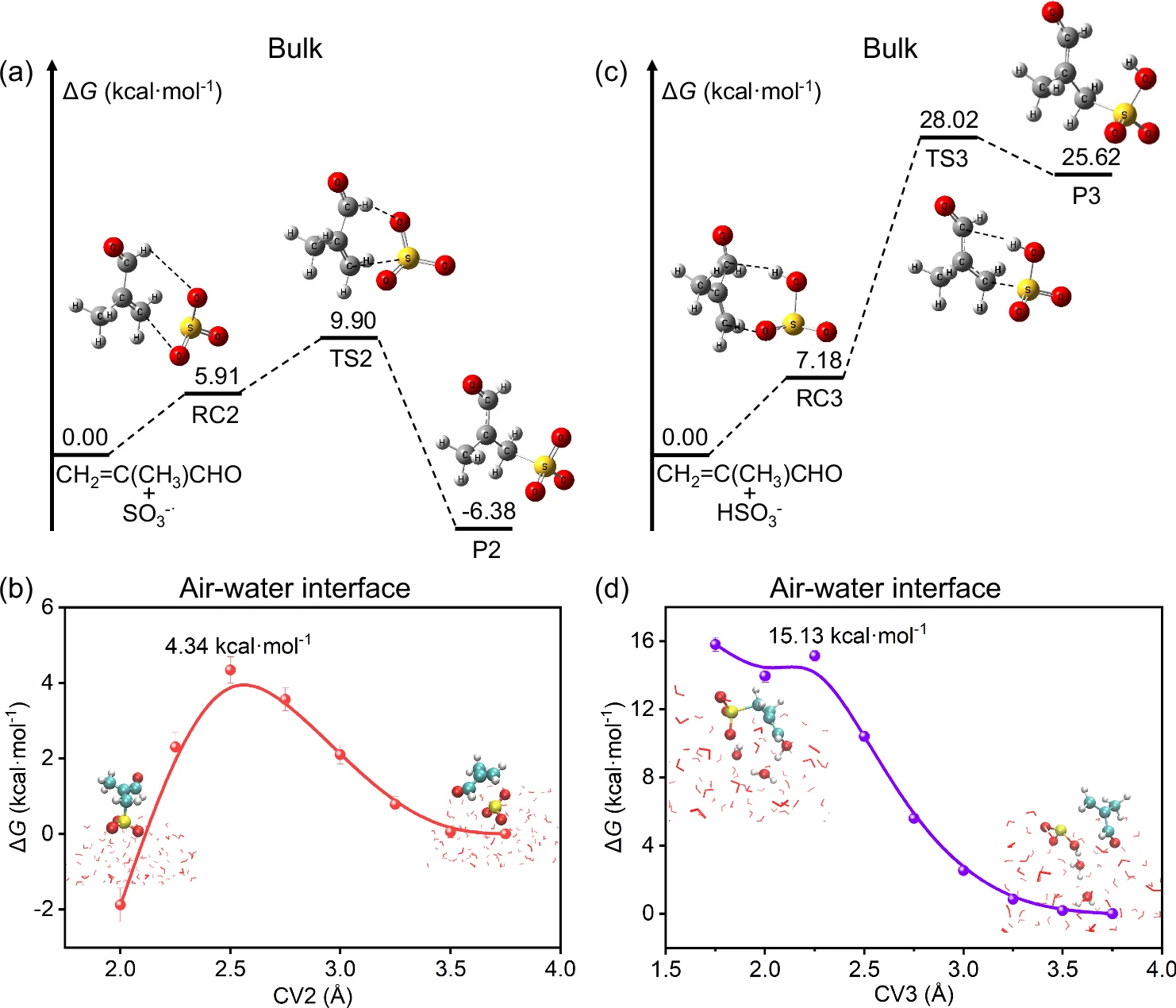
Microdroplet interface facilitates SO_3_^-•^ and HSO_3_^-^ addition to methacrolein (CH_2_ = C(CH_3_)CHO) by lowering the reaction barrier. Calculated Gibbs free energy (Δ*G*) for SO_3_^-•^ addition to the C=C bond of methacrolein **a** in bulk solution and **b** at the air-water interface of microdroplets. Calculated Δ*G* for HSO_3_^-^ addition to the C=C bond of methacrolein **c** in bulk solution and **d** at the air-water interface of microdroplets. Free energy profiles were obtained using thermodynamic integration. Error bars represent the standard error of the mean (SEM) estimated by block averaging time-series data from a single molecular dynamics (MD) trajectory at each *λ* value. Pre-reactive complex (RC), transition state (TS), product (P), collective variable (CV).

To further elucidate these mechanisms, quantum chemical calculations and metadynamics simulations based on Born-Oppenheimer molecular dynamics (BOMD) were performed. Considering that the epoxide moiety of alpha-pinene oxide readily undergoes acid-catalyzed ring opening to form carbonyl-containing intermediates, the theoretical investigations focused on the reactions of HSO_3_^-^ with carbonyl compounds and SO_3_^-•^ with unsaturated OVOCs containing C=C bond. The calculations indicate that HSO_3_^-^ reacts with carbonyl compounds through a nucleophilic addition mechanism. The reaction is initiated by C–S bond formation, followed by proton transfer from HSO_3_^-^ to the carbonyl oxygen, mediated by a hydrogen-bonded bridge formed by two interfacial water molecules. The Gibbs free energy (Δ*G*) at the air-water interface is 11.47 kcal·mol^−1^ (Fig. 4b), which is 7.45 kcal·mol^−1^ lower than that in bulk solution (Fig. 4a), indicating that carbonyl sulfonation is kinetically enhanced in microdroplets. In addition, SO_3_^-•^ preferentially adds to the less substituted carbon of the unsaturated C=C bond, with a Δ*G* of 4.34 kcal·mol^−1^ at the air-water interface (Fig. 5b), which is 5.56 kcal·mol^−1^ lower than that in bulk solution (Fig. 5a). The reduced reaction energy barrier at the air-water interface is widely attributed to the strong interfacial electric fields in microdroplets^22,74^. In addition, previous studies have demonstrated that external electric fields can further lower reaction barriers and thereby facilitate chemical reactions, highlighting the potential role of interfacial electric fields in modulating microdroplet reactivity^76,77^. In contrast, direct addition of HSO_3_^-^ to the C=C bond exhibits a substantially higher activation barrier (15.13 kcal·mol^−1^) (Fig. 5d). These results indicate that alkene sulfonation at the air-water interface proceeds predominantly via an SO_3_^-•^-mediated radical pathway rather than direct nucleophilic addition by HSO_3_^-^. Overall, the theoretical results suggest that interfacial electric fields and interfacial water molecules facilitate the reactions. The combined experimental and computational evidence supports the proposed reaction mechanisms.

### Atmospheric implications

This remarkable acceleration highlights the unique chemical environment at the air-water interface of microdroplets, where strong electric field and high surface-to-volume ratios can drastically alter reaction kinetics. Such interfacial effects have significant implications for atmospheric chemistry. In the atmosphere, OVOCs bearing C=C bond, C=O bond or epoxide group, are abundant and originate from both biological and anthropogenic sources. Micrometer-sized aqueous droplets containing dissolved SO_3_^2-^ are also ubiquitous, such as aerosol and cloud water, which can act as unique microreactors for atmospheric chemical reactions. Hence, based on our laboratory findings that SO_3_^2-^ can be spontaneously oxidized at the microdroplet interface by the intrinsic strong electric field to form SO_3_^-•^, which subsequently react with OVOCs to form organosulfur compounds, we propose that this reaction pathway is likely widespread and efficient under real atmospheric conditions. Notably, the transformation in microdroplets proceeds on a timescale that is orders of magnitude faster than that in bulk water (Fig. 3a), indicating that atmospheric microdroplets could serve as highly reactive sites for organosulfur compounds formation. Overall, these findings imply that atmospheric microdroplets play a significant role in atmospheric chemistry, which deserves further detailed investigations.

**Fig. 6:**
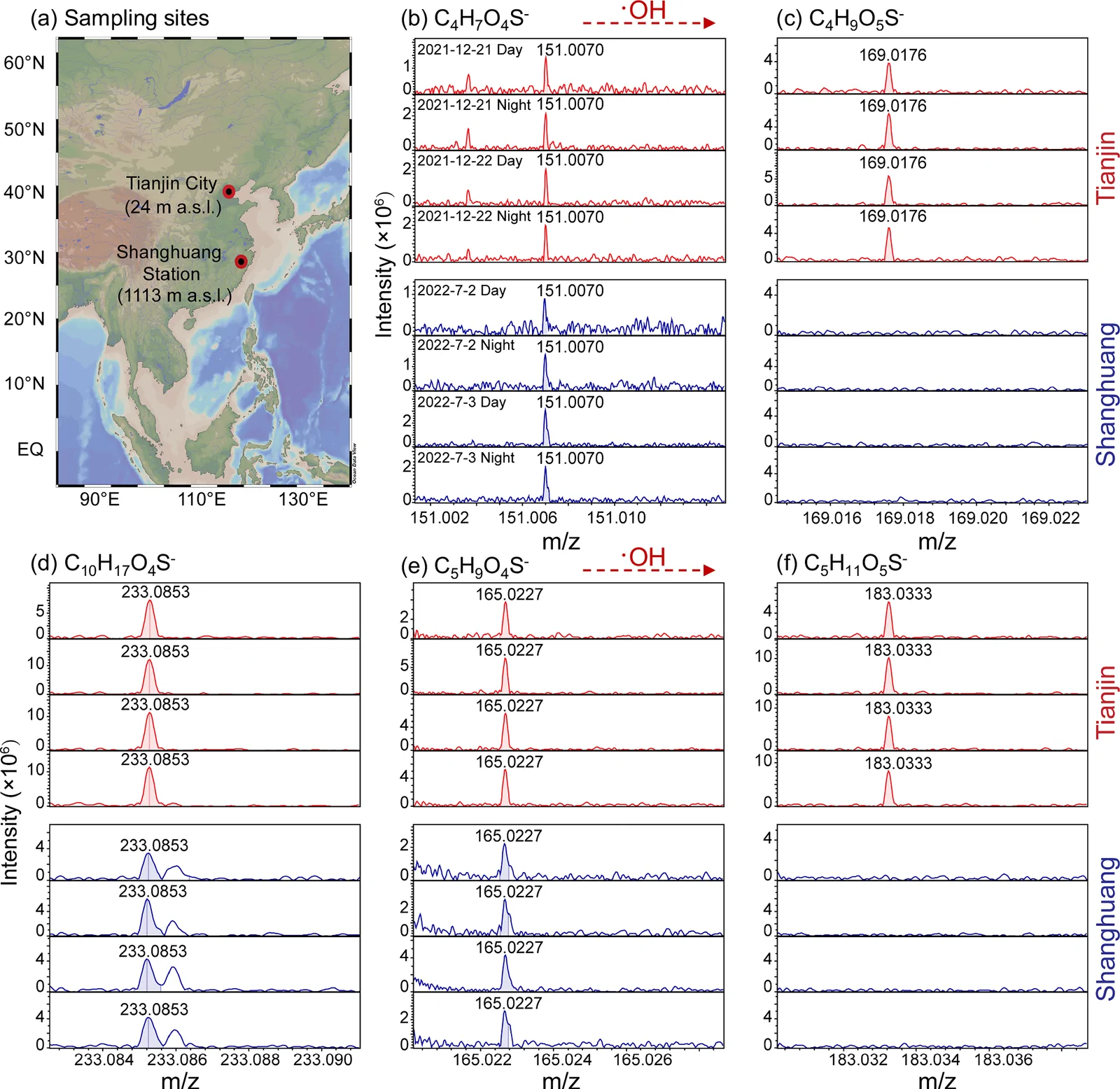
Detection of organosulfur compounds in atmospheric aerosols. **a** Ambient aerosol sampling sites at Shanghuang Station and in Tianjin, China. **b**–**f** Fourier transform ion cyclotron resonance mass spectrometry (FT-ICR MS) peaks corresponding to organosulfur compounds detected in aerosol samples collected from Tianjin (red) and Shanghuang Station (blue): **b** mass-to-charge ratio (*m*/*z*) 151.0070, **c** *m*/*z* 169.0176, **d** *m*/*z* 233.0853, **e** *m*/*z* 165.0227, and **f** *m*/*z* 183.0333.

The ambient aerosol samples were collected from a high-mountain site at the Shanghuang Station and in urban Tianjin, representing a typical background site with minimal local influence from anthropogenic sources and an urban site with severe air pollution, respectively. The geographic locations of the sampling sites are shown in Fig. 6a, generated using Ocean Data View (ODV)^78^. The molecular composition of organosulfur species in the aerosol samples was analyzed using ESI-FT-ICR MS in negative ion mode. ESI-FT-ICR MS method for the analysis of dissolved organic matter (DOM) is conducted at the molecular formula level rather than resolving exact molecular structures, and for accuracy considerations, the analysis generally focuses on compounds with *m*/*z* values greater than 150.

As shown in Fig. 6, the peaks at *m*/*z* 151.0070 (C_4_H_7_O_4_S^-^), *m*/*z* 165.0227 (C_5_H_9_O_4_S^-^) and *m*/*z* 233.0853 (C_10_H_17_O_4_S^-^) were consistently detected in eight aerosol samples collected from Tianjin and Shanghuang Station, and the peaks at *m/z* 169.0176 (C_4_H_9_O_5_S^-^) and *m*/*z* 183.0333 (C_5_H_11_O_5_S^-^) were only observed in four aerosol samples from Tianjin. These results provide strong evidence that organosulfur compounds generated in microdroplets under laboratory conditions are also prevalent in real atmospheric environments. Notably, the absence of the peaks at *m*/*z* 169.0176 (C_4_H_9_O_5_S^-^) and *m*/*z* 183.0333 (C_5_H_11_O_5_S^-^) in samples collected from Shanghuang Station may be associated with the higher relative humidity (67–69%) at this background site compared with that in Tianjin (53–59%) (https://www.ncei.noaa.gov/maps/lcd). The increased aerosol liquid water content under higher relative humidity could promote particle growth, potentially weakening the interfacial electric field strength of microdroplets due to the decreased curvature^32,39^, thereby reducing the generation of •OH and subsequently suppressing the further oxidation of the *m*/*z* 151.0070 (C_4_H_7_O_4_S^-^) and *m*/*z* 165.0227 (C_5_H_9_O_4_S^-^) product^79^. In addition, lower OVOC precursor abundance at Shanghuang station^80,81^ may contribute to the absence of these products. Moreover, the signal intensity of *m*/*z* 233.0853 (C_10_H_17_O_4_S^-^) is higher in samples from Tianjin than in those from Shanghuang, which may be due to higher concentrations of anthropogenic OVOCs and more favorable interfacial oxidation conditions. Organosulfur products identified in both laboratory simulations and field observations, such as C_4_H_8_O_4_S, C_5_H_10_O_4_S and C_10_H_18_O_4_S, may serve as potential tracers of SOA.

To further confirm the structure of the organosulfur compounds detected in ambient aerosols, MS/MS analyses were performed using an LTQ Orbitrap Velos on aerosol sample collected from Tianjin. The MS/MS measurements were carried out in LTQ mode with low resolution because of weak and unstable product ion signals. As shown in Fig. S18, the characteristic fragment ions from the ambient aerosol were consistent with those generated in the microdroplet experiments. In particular, the fragment ion at *m*/*z* 81 (HSO_3_^-^) was observed in all organosulfur products generated in microdroplet experiments and ambient samples, indicating the presence of sulfonate functional groups. Together with the agreement in accurate mass measurements from FT-ICR MS, the MS/MS fragmentation patterns provide evidence that the organosulfur compounds formed in microdroplets are structurally analogous to those present in atmospheric aerosols, supporting the atmospheric relevance of the proposed microdroplet formation pathway.

## Discussion

Our study unveils the compellingly rapid and spontaneous generation of organosulfur from inorganic sulfur at the air-water interface of microdroplets via two pathways: nucleophilic addition of HSO_3_^-^ to carbonyl groups and epoxide groups, and radical addition of SO_3_^-•^ to C=C bond of OVOCs. This SO_3_^-•^ is formed through one-electron loss from the SO_3_^2-^ driven by the strong electric field of microdroplets. Strikingly, the reaction of organosulfur generation proceeds on a timescale several orders of magnitude faster in microdroplets than in bulk solution, despite the absence of any catalysts, oxidants, light, or external energy input that are typically required under bulk-phase conditions. Theoretical calculations indicate that strong interfacial electric fields facilitate organosulfur formation by significantly reducing the activation barriers of the reactions. Furthermore, key organosulfur products formed in laboratory microdroplets were identified in ambient aerosol samples, indicating that this reaction pathway is prevalent under real atmospheric conditions. Taken together, these findings reveal an overlooked heterogeneous pathway for the conversion of inorganic sulfur to organosulfur in the atmosphere. This work highlights the potentially important role of microdroplets and aerosol interfacial chemistry in atmospheric transformation and suggests that such processes may need to be considered in future predictive atmospheric models.

## Methods

### Chemicals required

Sodium sulfite (Na_2_SO_3_) was purchased from Shanghai Macklin Biochemical Co., Ltd. Glyoxal (C_2_H_2_O_2_), methacrolein (C_4_H_6_O), 3-methyl-2-butenal (C_5_H_8_O), alpha-pinene oxide (C_10_H_16_O), campholenic aldehyde (C_10_H_16_O), 2,2,6,6-tetramethyl-1-piperidinyloxy (TEMPO), tert-butanol (TBA), hydroxymethanesulfonate (HMS) and chromatographic grade methanol were purchased from Shanghai Aladdin Biochemical Technology Co., Ltd. Stock solutions (10 mM) of glyoxal, alpha-pinene oxide, methacrolein, 3-methyl-2-butenal, campholenic aldehyde, TBA, HMS and 2,2,6,6-tetramethyl-1-piperidinyloxy were firstly obtained by dissolving in methanol and stored at −20 °C, respectively. Then the solutions with desired concentrations were made by diluting the stock solutions in water. Na_2_SO_3_ was freshly prepared in Milli-Q water on the day of the experiment. Milli-Q water (18.2 MΩ·cm) used for all experiments was purified through a Milli-Q ultrapure water system (Merck Millipore, Burlington, USA). Deionized and distilled water were purchased from BKMAMLAB. Compressed nitrogen (N_2_, 99.9 %) was supplied by Air Liquide.

### Generation of microdroplets and mass spectrometric analysis

Figure 1a illustrates the experimental setup for the microdroplet reactions using a simple nebulized spray system, and a photographic image of the setup is displayed in Fig. S1. Briefly, an aqueous solution of sodium sulfite (Na_2_SO_3_), either alone or mixed with a representative OVOC (glyoxal, alpha-pinene oxide, methacrolein, or 3-methyl-2-butenal) was sprayed through a fused silica capillary (inner diameter: 100 µm, outer diameter: 193 µm) using a syringe pump operating at a flow rate of 15 µL·min^−1^, and high purity N_2_ at high pressure (100 psi) served as the nebulizing gas. The high-pressure N_2_ assists the nebulization of the liquid stream, where the pressure-driven gas flow exerts aerodynamic shear that fragments the liquid into micron-sized droplets through pneumatic atomization, producing microdroplets with large surface-to-volume ratios. The reaction products were detected and analyzed by LTQ Orbitrap Velos (Thermo Fisher Scientific, Bremen, Germany) at a mass resolving power of 30,000. Full-scan negative ion mode across a *m*/*z* range from 50 to 1000 was selected. The inlet capillary temperature of the mass spectrometer was maintained at 350 °C. The distance between the outlet of the nebulizer and the inlet of the mass spectrometer was defined as the reaction distance, within which the flight time of the microdroplets represents the reaction time^48,49^. In addition, the concentration-dependent and distance-dependent experiments were performed. Owing to the lack of commercially available pure standards for these products, the samples were spiked with HMS as internal standards for semi-quantitative analysis. Unlike electrospray ionization, no external voltage was applied to the solutions. Collision-induced dissociation (CID) was also conducted to identify the structure of the reaction products.

The nebulization and subsequent microdroplet reactions were carried out under ambient laboratory conditions (temperature: ~26 °C, relative humidity: ~47%). The freshly prepared mixed solutions of Na_2_SO_3_ and OVOCs exhibited near-neutral pH values ranging from 6.81 to 7.57 prior to spraying, as measured using a pH meter (Star A215, Thermo Scientific, USA). For comparison, bulk-phase reactions were performed in a 5 mL glass reactor at room temperature using the same initial concentrations of Na_2_SO_3_ and OVOCs as those used in the microdroplet experiments.

### Control experiments for dissolved oxygen and trace transition metals

To evaluate the potential contribution of dissolved oxygen to SO_3_^2-^ oxidation, microdroplet experiments were performed using freshly prepared and boiled ultrapure water. The dissolved oxygen concentrations were measured using a YSI Pro20i dissolved oxygen meter (YSI, Yellow Springs, OH, USA). To assess the influence of trace transition metals, ultrapure water, deionized water, and distilled water were used as reaction media. The concentrations of trace metals in these water matrices were determined by inductively coupled plasma mass spectrometry (ICP-MS, 7900, Agilent Technologies, USA). Microdroplet experiments were subsequently conducted under identical conditions using each water matrix.

### ATR-FTIR and UPLC-MS analysis

For structural characterization, microdroplets were collected into a 2 mL brown glass vial over a period of 1 h. The organosulfur products formed in the microdroplets were analyzed using attenuated total reflectance Fourier-transform infrared spectroscopy (ATR-FTIR; Bruker Optics, Ettlingen, Germany). Spectra were recorded over the range of 4000–400 cm^−1^ with a resolution of 4 cm^−1^ accumulating 32 scans per sample. The background absorption of methanol was subtracted to obtain the final infrared spectra.

Chromatographic separation was performed using a Shimadzu LC-30A ultra-high performance liquid chromatography (UPLC) system coupled to a Shimadzu Q-TOF mass spectrometer (9030, Kyoto, Japan) equipped with an electrospray ionization (ESI) source in negative ion mode. Separation was performed on an Acquity HSS T3 column (1.8 µm, 2.1 × 100 mm, Waters) maintained at 40 °C. The gradient elution started with 100% mobile phase A (10 mM ammonium acetate) at a flow rate of 0.35 mL·min^−1^ for 1.7 min, then changed to 100% of mobile phase B (methanol) with a flow rate 0.25 mL·min^−1^ in 4.7 min and maintained constant for 1 min. The mass spectrometer scanned over *m*/*z* 50–1000 with a scan time of 0.1 s. Data acquisition and processing were performed using LabSolution software.

### Theoretical methods

All quantum chemical calculations were performed using the Gaussian 16 software package^82^. Geometry optimizations and frequency calculations were carried out at the M06-2X^83^/6–311+ +G(d,p) level. Solvent effects were incorporated in all calculations via the continuum solvation model (SMD)^84^. To confirm the validity of the transition state structures, intrinsic reaction coordinate (IRC) analyses were conducted^85^, verifying that each transition state connects the two designated minima.

Metadynamics simulations based on Born-Oppenheimer molecular dynamics (BOMD) were performed using the CP2K software to investigate the reaction mechanism at the air-water interface^86^. The interfacial model was constructed with a 35 Å cubic box containing 191 water molecules; this box size reproduces the correct water density and prevents spurious long-range interactions between periodic images^87,88^. Prior to the BOMD simulations, the system was fully relaxed using the Becke-Lee-Yang-Parr functional^89,90^ with Grimme’s dispersion correction^91,92^ to accurately describe weak dispersion interactions. The electronic structure was treated with a double-ζ basis set in conjunction with Goedecker-Teter-Hutter norm-conserving pseudopotentials and the corresponding auxiliary basis set^93–95^; the plane-wave cutoff and the cutoff for the Gaussian basis set were set to 280 and 40 Ry, respectively. All dynamics were propagated in the NVT ensemble at 300 K using a Nosé-Hoover chain thermostat with an integration time step of 1 fs^96,97^.

The free energy of the reaction was computed using the two-step metadynamics coupled with thermodynamic integration (TMTI) strategy^98^. This approach combines metadynamics, multiple-walker metadynamics^99^, and thermodynamic integration^100^ in a two-step “pathway + energy” scheme. The reaction path is identified by deploying high and wide Gaussian hills along a one-dimensional collective variable, and the free energy is subsequently refined along the obtained path through parallel multiple-walker sampling or point-by-point thermodynamic integration, enabling efficient and high-fidelity reconstruction of the free energy surface for complex reaction systems.

### Aerosol sample collection

The fine particulate matter (PM_2.5_, aerodynamic diameter < 2.5 μm) samples were collected at the rooftop of a teaching building (approximately 21 m above ground level) of Tianjin University (39°06′26″N, 117°10′25″E). Sampling was conducted on a 12 h basis from 21 to 22 December 2020 by a high-volume sampler (Tisch TE-PM_2.5_ HVP-BL). The PM_2.5_ samples were collected on a 12 h basis from 2 to 3 July 2022 at Shanghuang Eco-Environmental Observatory of the Chinese Academy of Sciences, located at Mt. Damaojian (28°34′48″N, 119°30′36″E) in Wuyi County, Zhejiang Province. The sampler was cleaned and calibrated regularly according to standard procedures, and the flow rate was maintained at 1.0 m^3^·min^−1^. All aerosol samples were collected onto pre-combusted quartz fiber filters (20 cm × 25 cm, Pallflex) that had been heated at 450 °C for 6 h in a muffle furnace. After the sampling, the filters were stored in a refrigerator at −20 °C until analysis.

### DOM preparation and solid-phase extraction

Approximately 6 cm^2^ of each quartz filter was put into a glass bottle (pre-combusted at 450 °C, 6 h) to extract three times, with 10 mL ultrapure Milli-Q to sonicate for 10 min each time. The extract was combined and filtered by 0.45 μm PTFE filters. The hydrophilic-lipophilic balance (Oasis HLB) solid-phase extraction (SPE) cartridges (200 mg, 6 mL, Waters Corporation, USA) were pretreated with 15 mL of methanol and 10 mL of Milli-Q water. Each set of samples was percolated through a SPE cartridge. Then, the cartridges were rinsed with 5 mL of Milli-Q water and dried under a nitrogen flow for 1 h and eluted using 6 mL rinses of methanol. The extract was concentrated to 1 mL by a rotary evaporator (BUCHI, Switzerland). Finally, they were stored at −20 °C until analysis.

The pretreated extracts were analyzed by an ESI FT-ICR MS (Bruker Daltonik, GmbH, Bremen, Germany) equipped with a 7.0 T superconducting magnet. Samples were analyzed by infusion into the ESI unit with the capillary voltage set to 4.0 kV in the negative ion mode. Before transferred into the ICR cell, ions were accumulated for 0.1 s in the hexapole. Within-mass spectrum limit from 150 to 1000 Da, 200 scans were collected. An average resolving power of over 400,000 (at *m*/*z* = 400 Da) was acquired. Control blanks were analyzed following the same procedure.

### FT-ICR MS analysis

The mass spectra received by FT-ICR MS were internally recalibrated using a reference list formed by manually assigned known formula in Data Analysis 4.0 (Bruker Daltonics). Molecular formula assignment was obtained in the mass range between 150 and 1000 Da, with elemental limits of ^12^C (1–50), ^1^H (1−100), ^16^O (1–50), ^14^N (0–4), and ^32^S (0–2). Only peaks with an error threshold of ±0.5 ppm and with a signal-to-noise (S/N) ratio ≥ 4 were considered for further analysis. Further restrictions for formula calculation were applied as follows: H/C ≤ 2.5, O/C ≤ 1.2, and N/C ≤ 0.5, nitrogen rule for even electron ions, limit that DBE (double bond equivalent) must be an integer value^101,102^. Unambiguous molecular formula assignment was determined with the help of the homologous series approach for multiple formula assignments^103,104^.

## Supplementary information

Supplementary Information

Transparent Peer Review file

## Data Availability

The datasets generated and analyzed during this study are available in the Zenodo repository: https://doi.org/10.5281/zenodo.21582618^105^.
