# Supplementary material for: Rapid spontaneous generation of organosulfur from inorganic sulfur in atmospheric microdroplets
Source: Nat Commun. 2026 Sep 10;17:10684. doi: 10.1038/s41467-026-77473-5 (PMC13655161; doi:10.1038/s41467-026-77473-5)
Supplement: Supplementary file 1 [file 41467_2026_77473_MOESM1_ESM.pdf]

Supplementary Information

Rapid Spontaneous Generation of Organosulfur from Inorganic Sulfur in Atmospheric Microdroplets

Huixia Han[1], Dongmei Zhang[1]*, Zegang Dong[2], Jiaqi Wang[1], Shuang Chen[1], Junjun Deng[1], Libin Wu[1], Wei Hu[1], Mingjin Tang[1], Bo Long[2], Jialei Zhu[1], Cong-Qiang Liu[1], Pingqing Fu[1]*

[1]Institute of Surface-Earth System Science, School of Earth System Science, Tianjin University, No. 92 Weijin Road, Nankai District, Tianjin 300072, China

[2]School of Materials Science and Engineering, Guizhou Minzu University, Guiyang 550025, China

*e-mails: zdm_1126@tju.edu.cn; fupingqing@tju.edu.cn.

Table S1. Concentrations of trace metals in different water matrices determined by inductively coupled plasma tandem mass spectrometry (ICP-MS)**.**

Sample	Ti	V	Cr	Mn	Fe	Co	Ni	Cu	Y	Mo
Ultrapure water	0.016	<0.000	0.006	<0.000	0.089	0.007	<0.000	0.009	<0.000	<0.000
Deionized water	<0.000	<0.000	<0.000	<0.000	<0.000	0.0	0.0	<0.000	<0.000	<0.000
Distilled water	<0.000	<0.000	0.0	<0.000	<0.000	<0.000	0.001	0.001	<0.000	<0.000

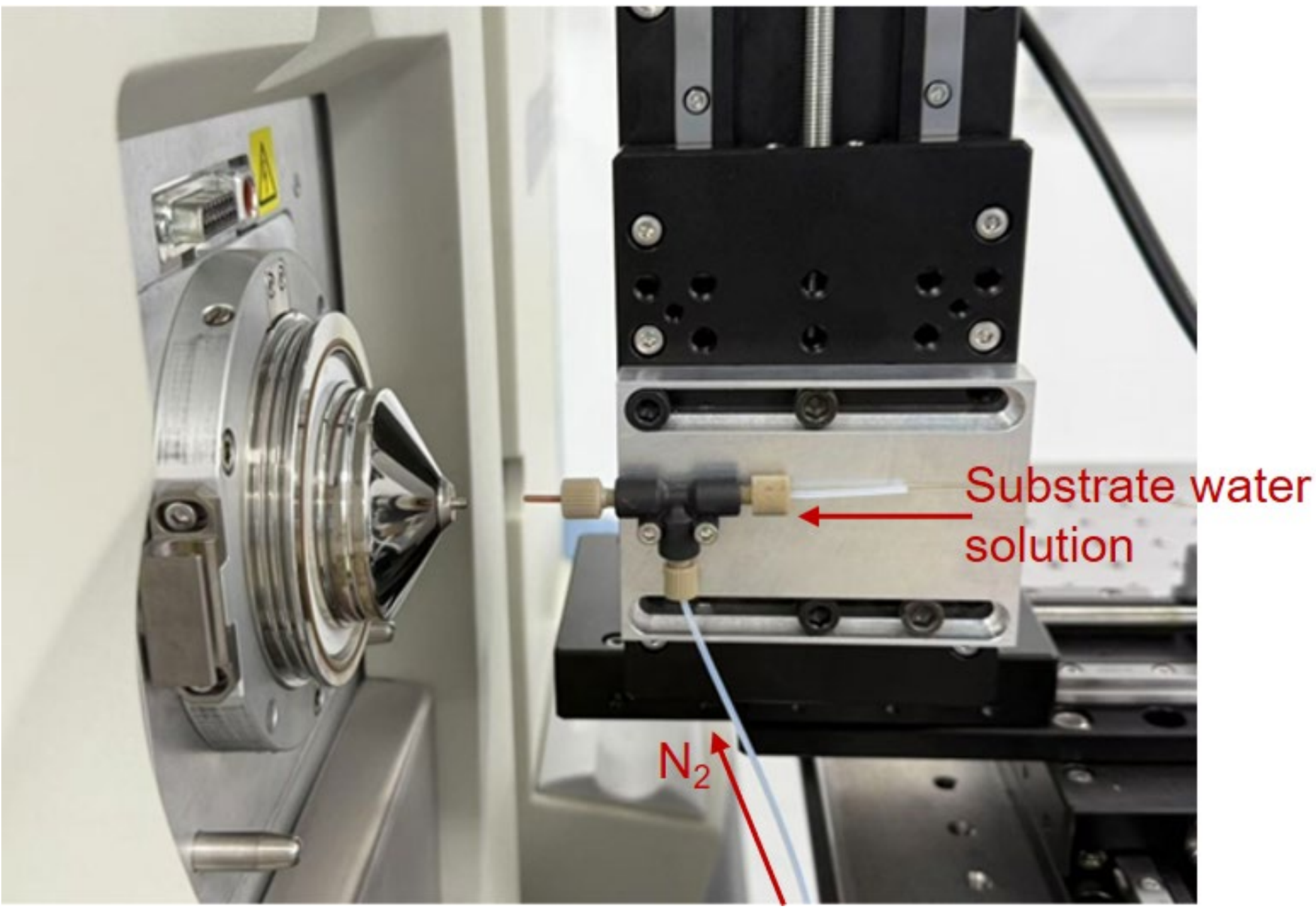

Figure S1. Photographic image of the experimental setup used for the microdroplet reactions.

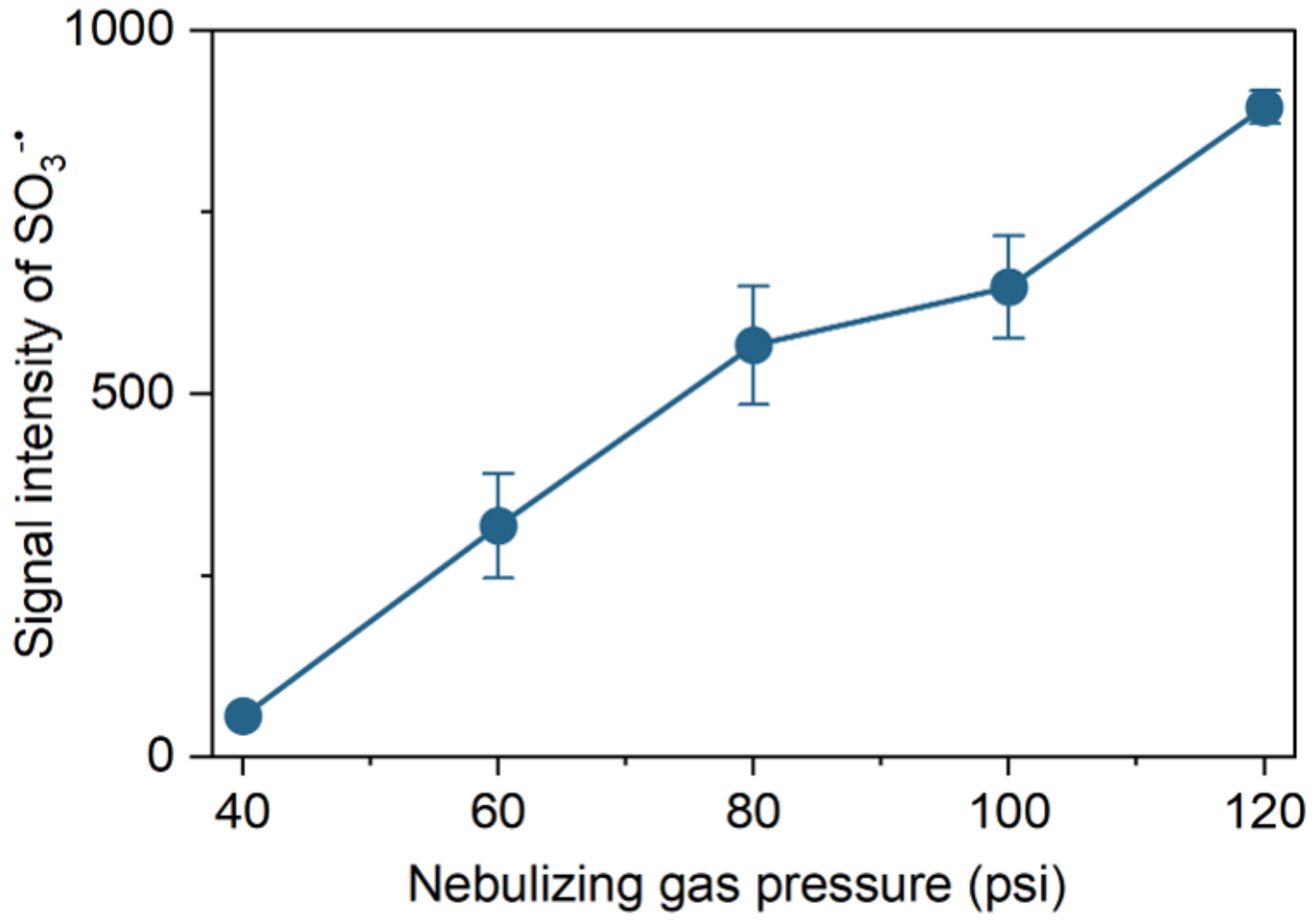

Figure S2. Effect of sheath gas pressure on $SO_3^{-\bullet}$ signal intensity in microdroplets generated from a 40 μM Na_2SO_3 solution at a reaction distance of 20 mm. Error bars (mean ± standard deviation (SD), n = 3)

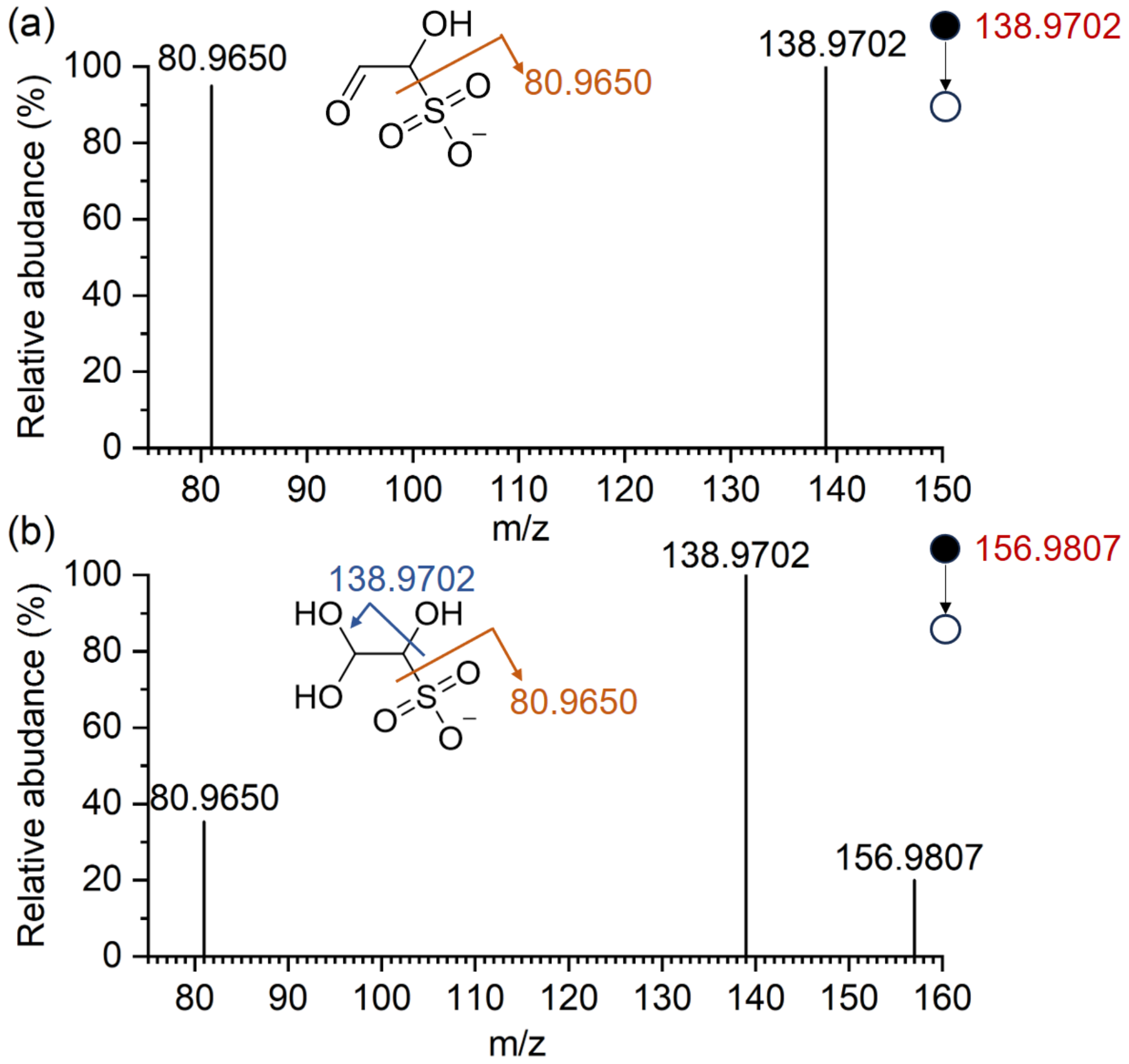

Figure S3. Tandem mass spectrometry (MS/MS) spectra when spraying Na_2SO_3 and glyoxal. (a) Mass-to-charge ratio (m/z) 138.9702. (b) m/z 156.9807.

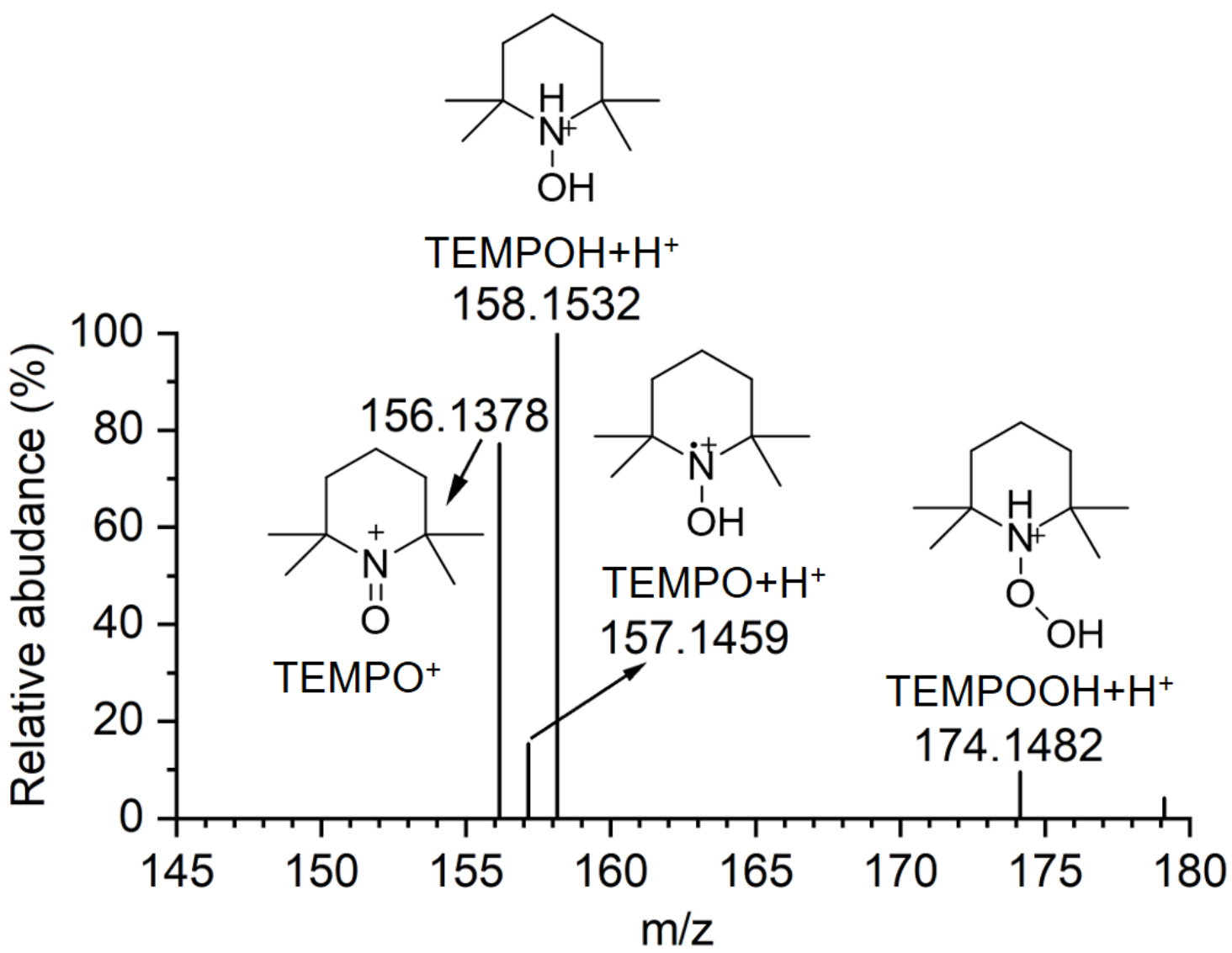

Figure S4. Capture of •OH by 2,2,6,6-tetramethyl-1-piperidinyloxy (TEMPO) at the air-water interface. Mass-to-charge ratio (m/z).

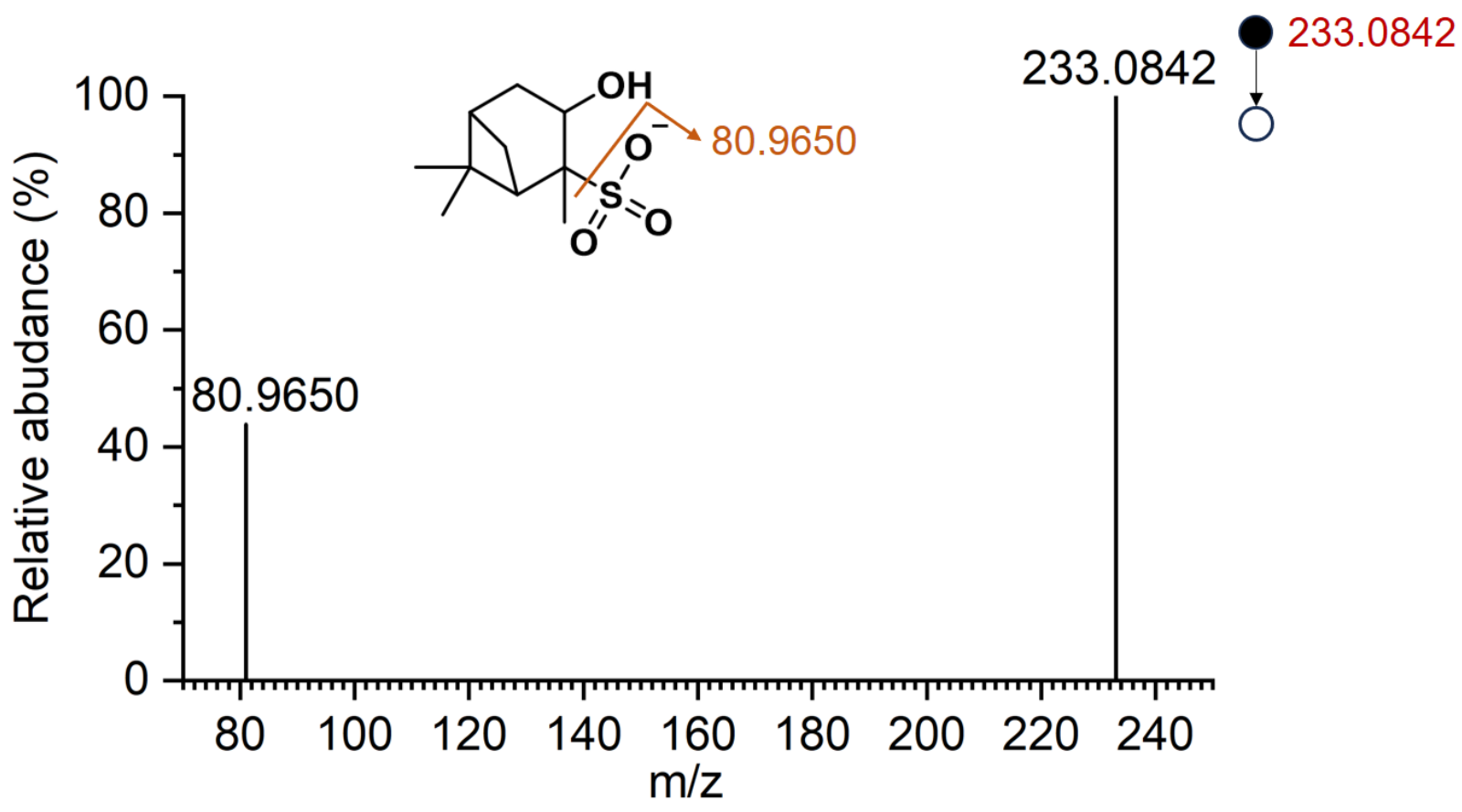

Figure S5. Tandem mass spectrometry (MS/MS) spectrum of mass-to-charge ratio (m/z) 233.0842 when spraying Na_2SO_3 and alpha-pinene oxide.

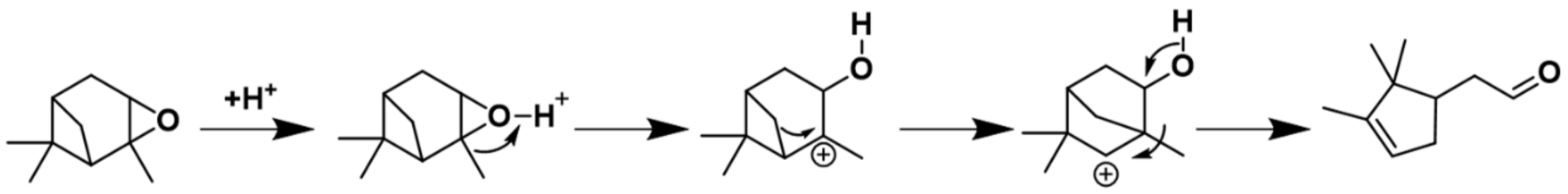

Figure S6. Scheme for the proposed acid catalyzed isomerization mechanisms of alpha-pinene oxide at the air-water interface of microdroplets.

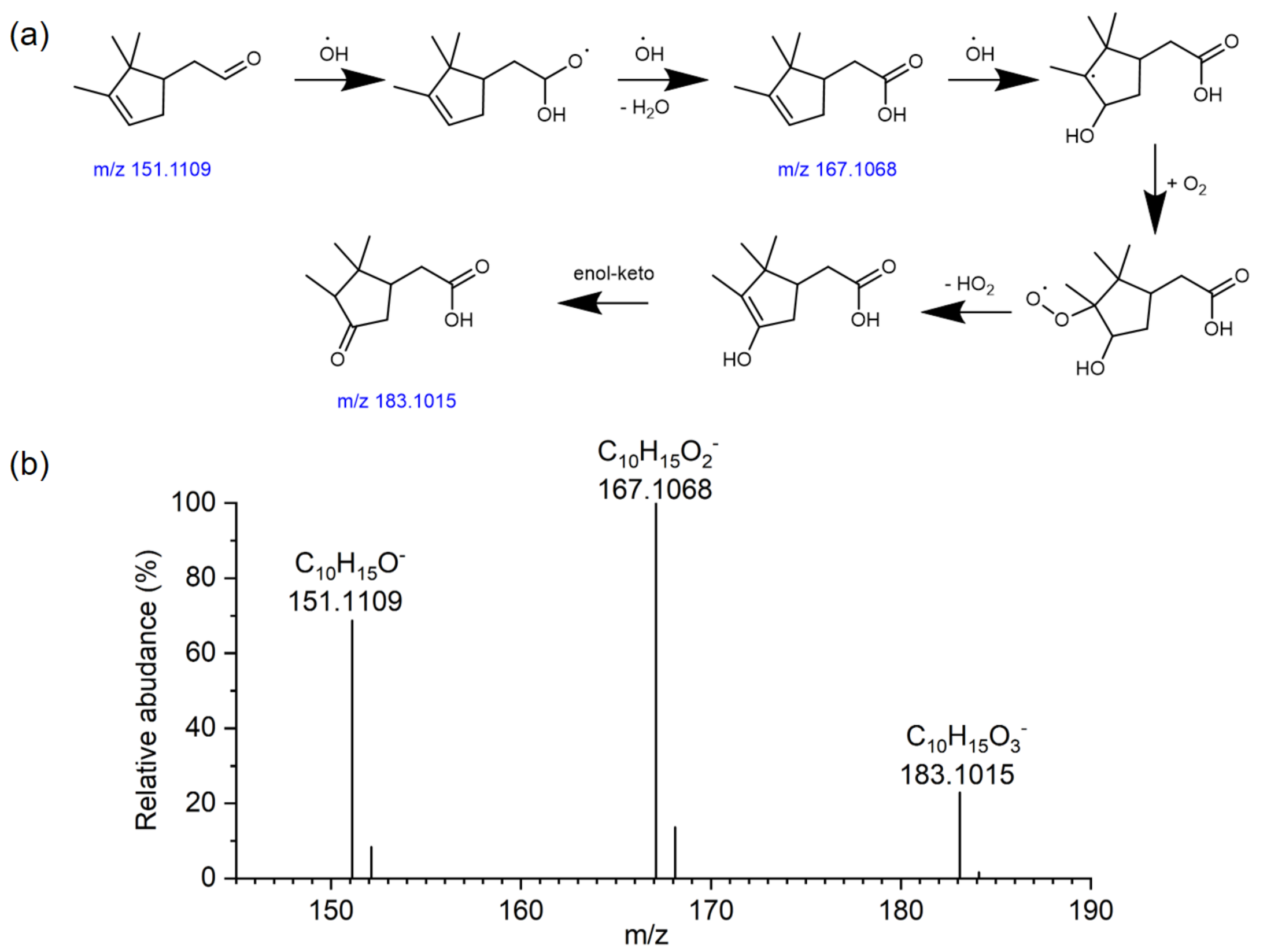

Figure S7. (a) Scheme for the proposed oxidation mechanisms of the campholenic aldehyde at the air-water interface of microdroplets**.** (b) Typical mass spectrum when spraying campholenic aldehyde standard reagent. Mass-to-charge ratio (m/z).

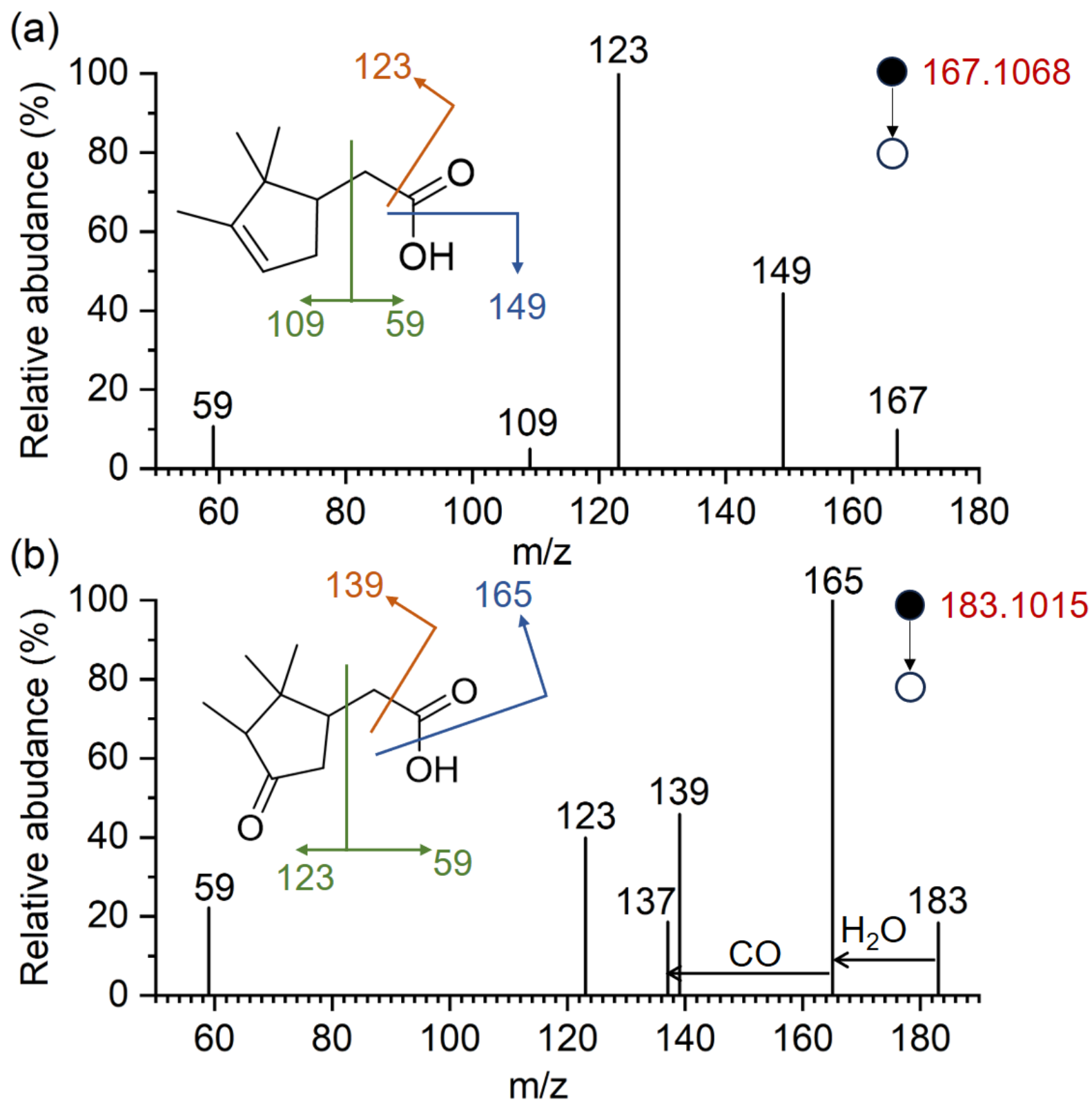

Figure S8. Tandem mass spectrometry (MS/MS) spectra when spraying Na_2SO_3 and alpha-pinene oxide. (a) Mass-to-charge ratio (m/z) 167.1069. (b) m/z 183.1017.

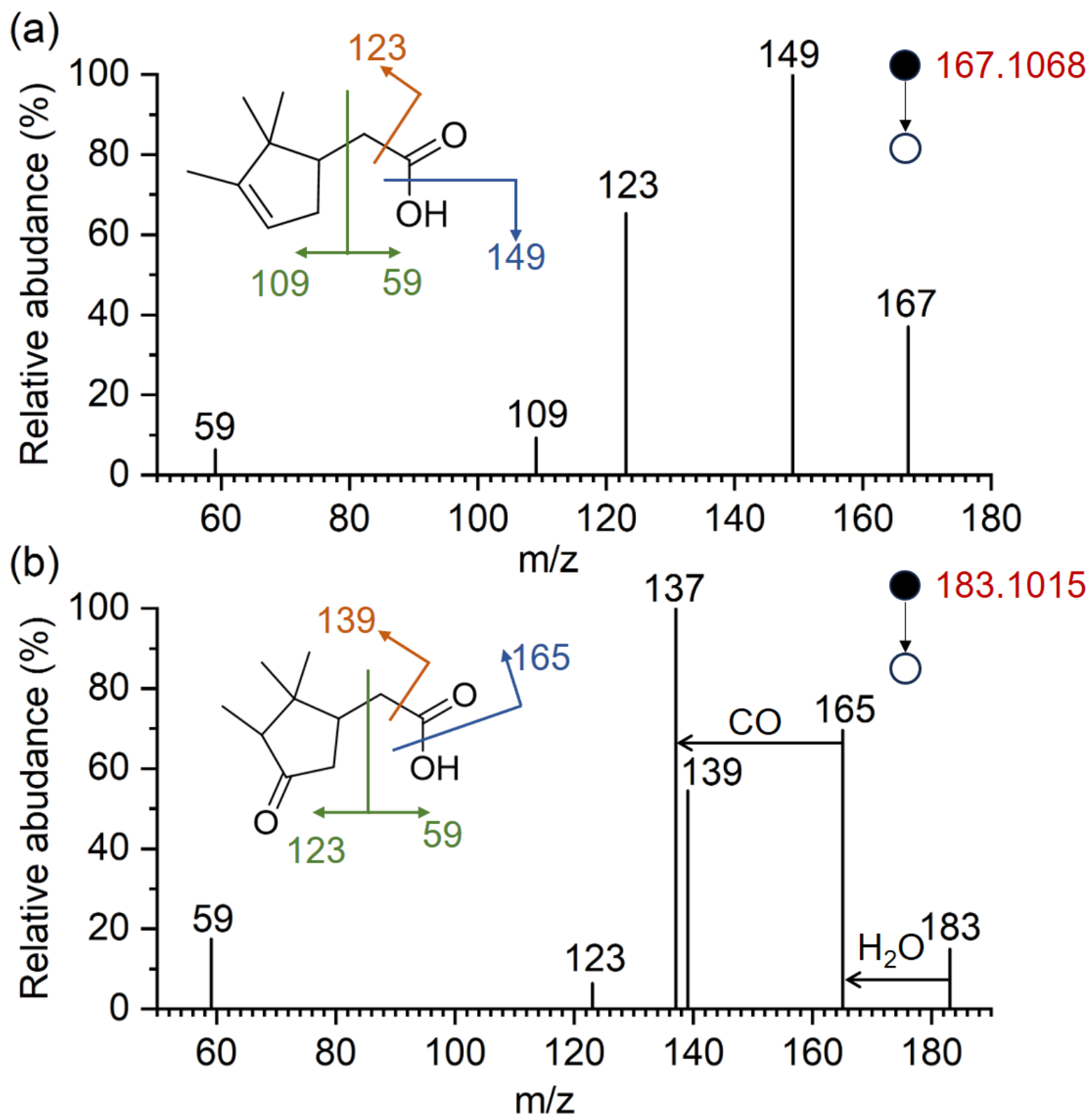

Figure S9. Tandem mass spectrometry (MS/MS) spectra when spraying campholenic aldehyde standard reagent. (a) Mass-to-charge ratio (m/z) 167.1069. (b) m/z 183.1017.

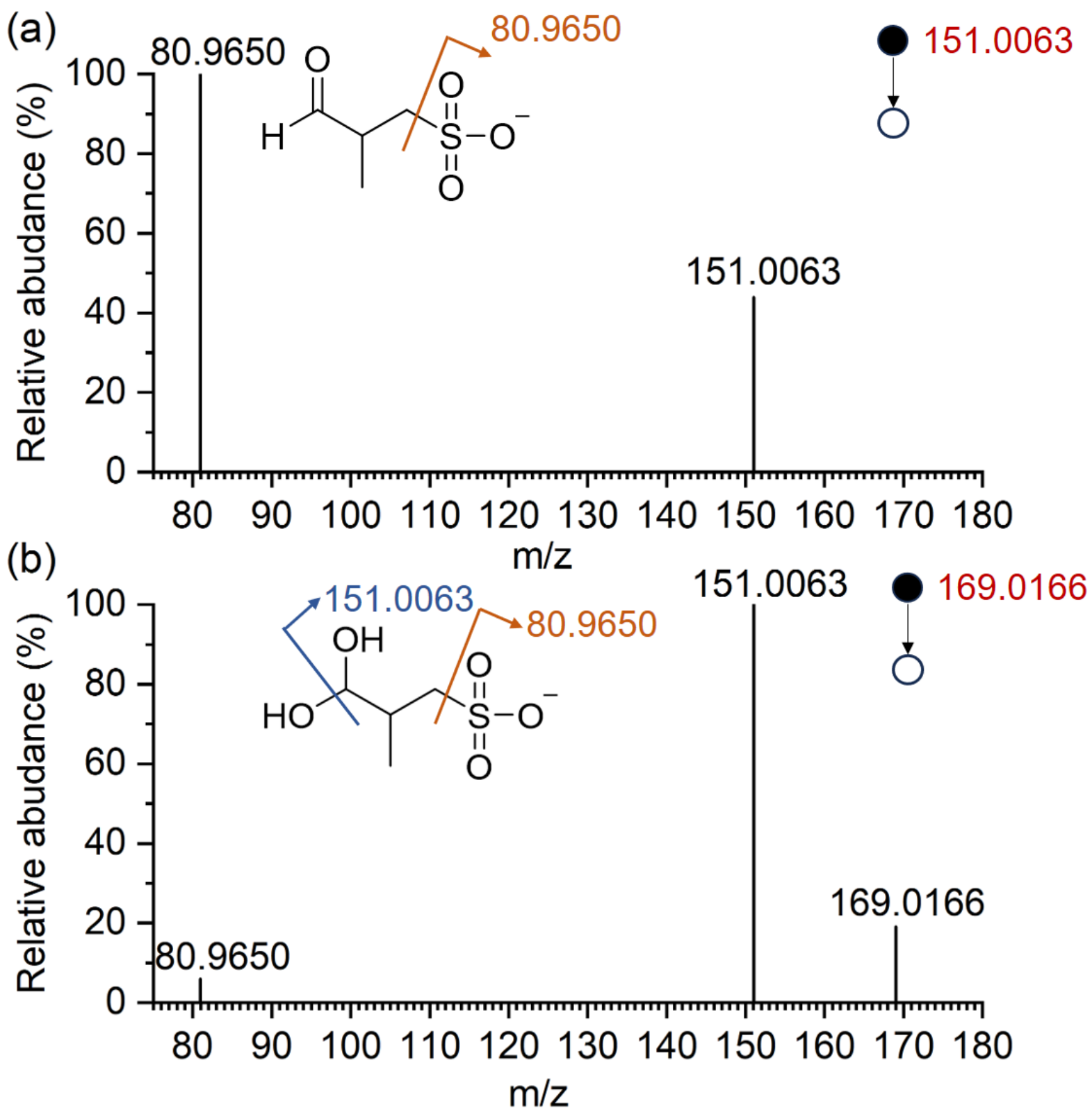

55

56 **Figure S10.** Tandem mass spectrometry (MS/MS) spectra when spraying Na_2SO_3 and

57 methacrolein. (a) Mass-to-charge ratio (m/z) 151.0063. (b) m/z 169.0166.

58

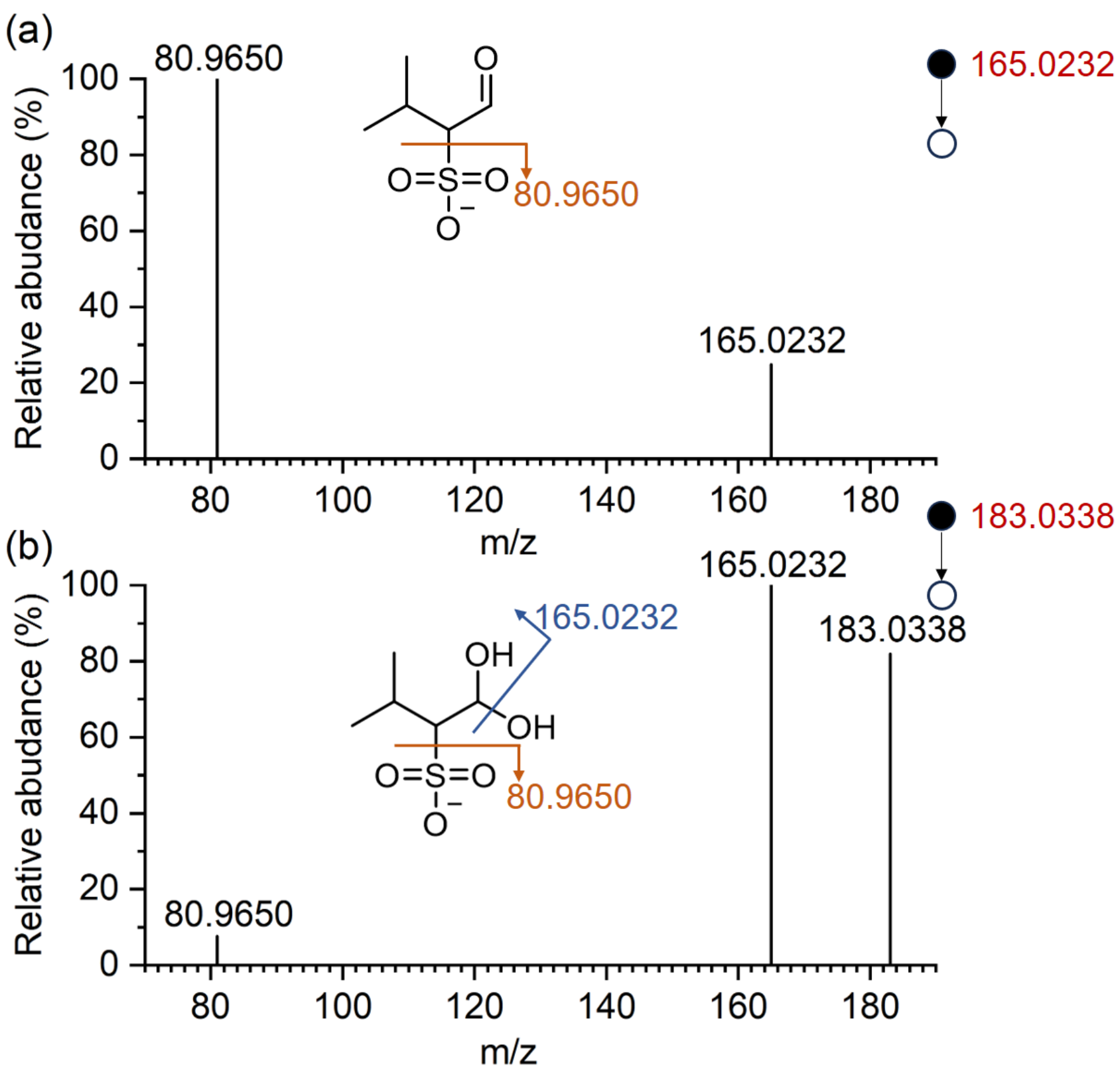

Figure S11. Tandem mass spectrometry (MS/MS) spectra when spraying Na_2SO_3 and 3-methyl-2-butenal. (a) Mass-to-charge ratio (m/z) 165.0232. (b) m/z 183.0338.

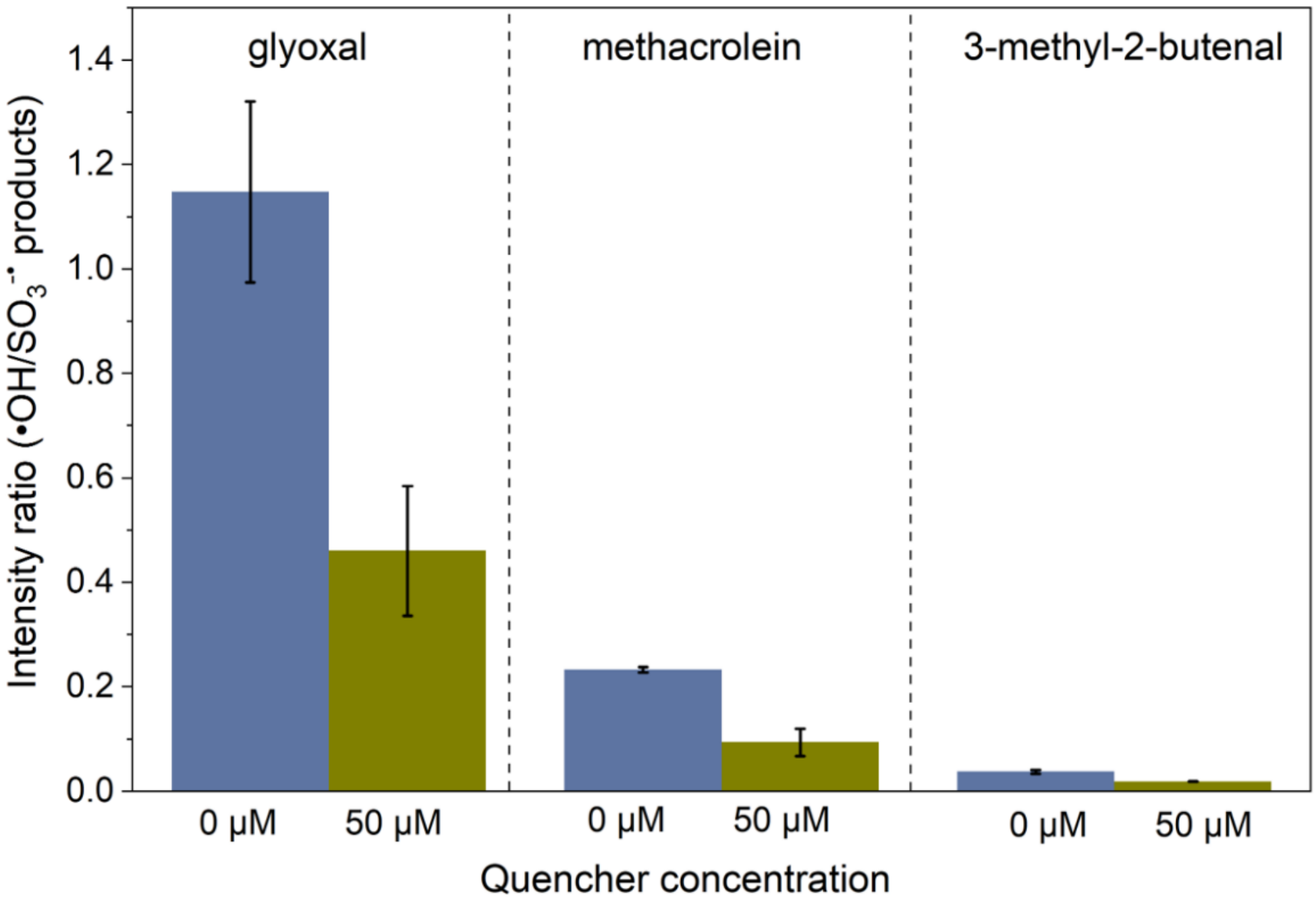

Figure S12. The intensity ratio of organosulfur products, $C_2H_5O_6S^-/C_2H_3O_5S^-$, $C_4H_9O_5S^-/C_4H_7O_4S^-$, and $C_5H_{11}O_5S^-/C_5H_9O_4S^-$, generated from the reactions of Na_2SO_3 with glyoxal, methacrolein, and 3-methyl-2-butenal, respectively, in the absence and presence of tert-butanol (TBA). Error bars (mean ± standard deviation (SD), n = 3).

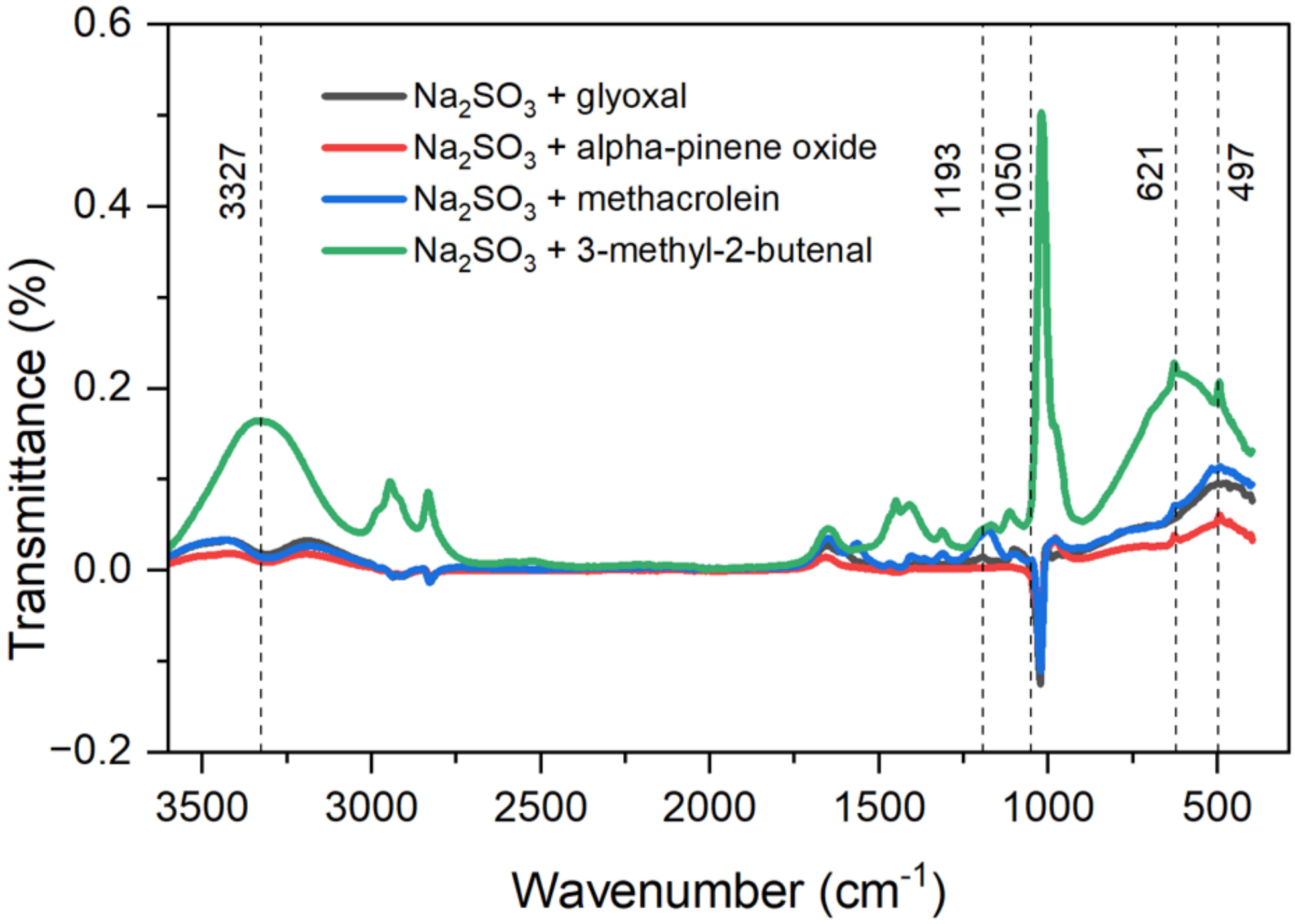

Figure S13. Attenuated total reflectance Fourier-transform infrared (ATR-FTIR) spectrum of the organosulfur products collected from microdroplet reactions of Na_2SO_3 with representative oxygenated volatile organic compounds (OVOCs).

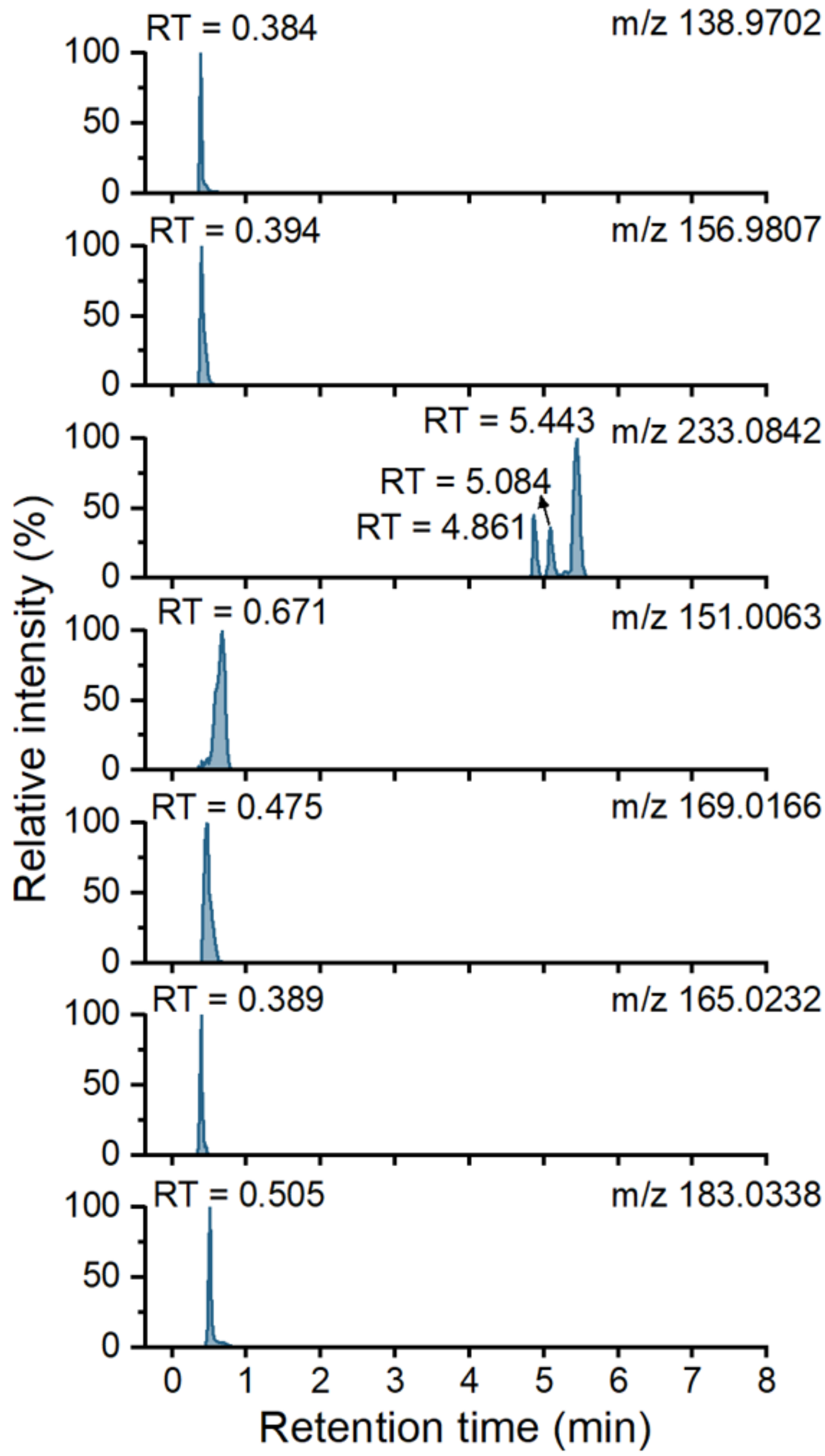

Figure S14. Extracted ion chromatograms (EICs) of the organosulfur products generated from reactions of Na_2SO_3 with glyoxal (mass-to-charge ratio (m/z) 138.9702, m/z 156.9807), alpha-pinene oxide (m/z 233.0842), methacrolein (m/z 151.0063, m/z 169.0166), and 3-methyl-2-butenal (m/z 165.0232, m/z 183.0338) in water microdroplets. Retention time (RT).

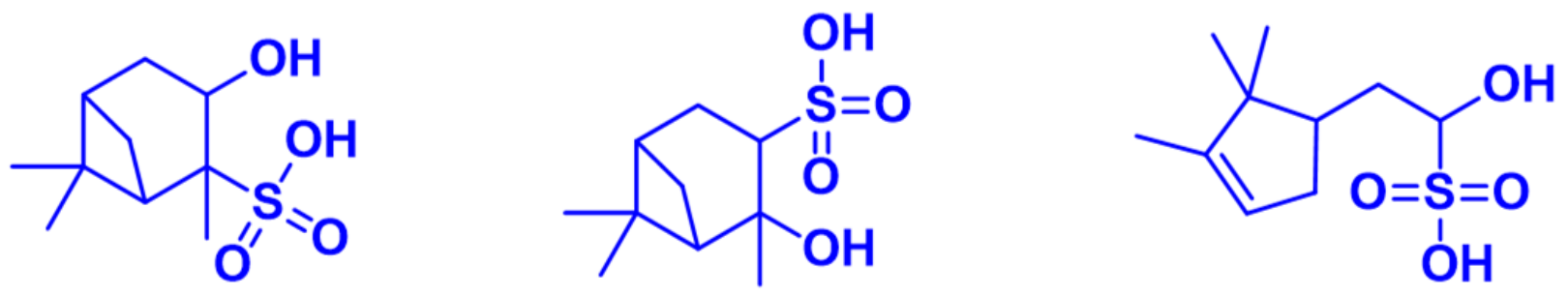

Compound 1 Compound 2 Compound 3

Figure S15. Proposed structures of mass-to-charge ratio (m/z) 233.0842 generated from the reaction of alpha-pinene oxide with Na_2SO_3 in water microdroplets.

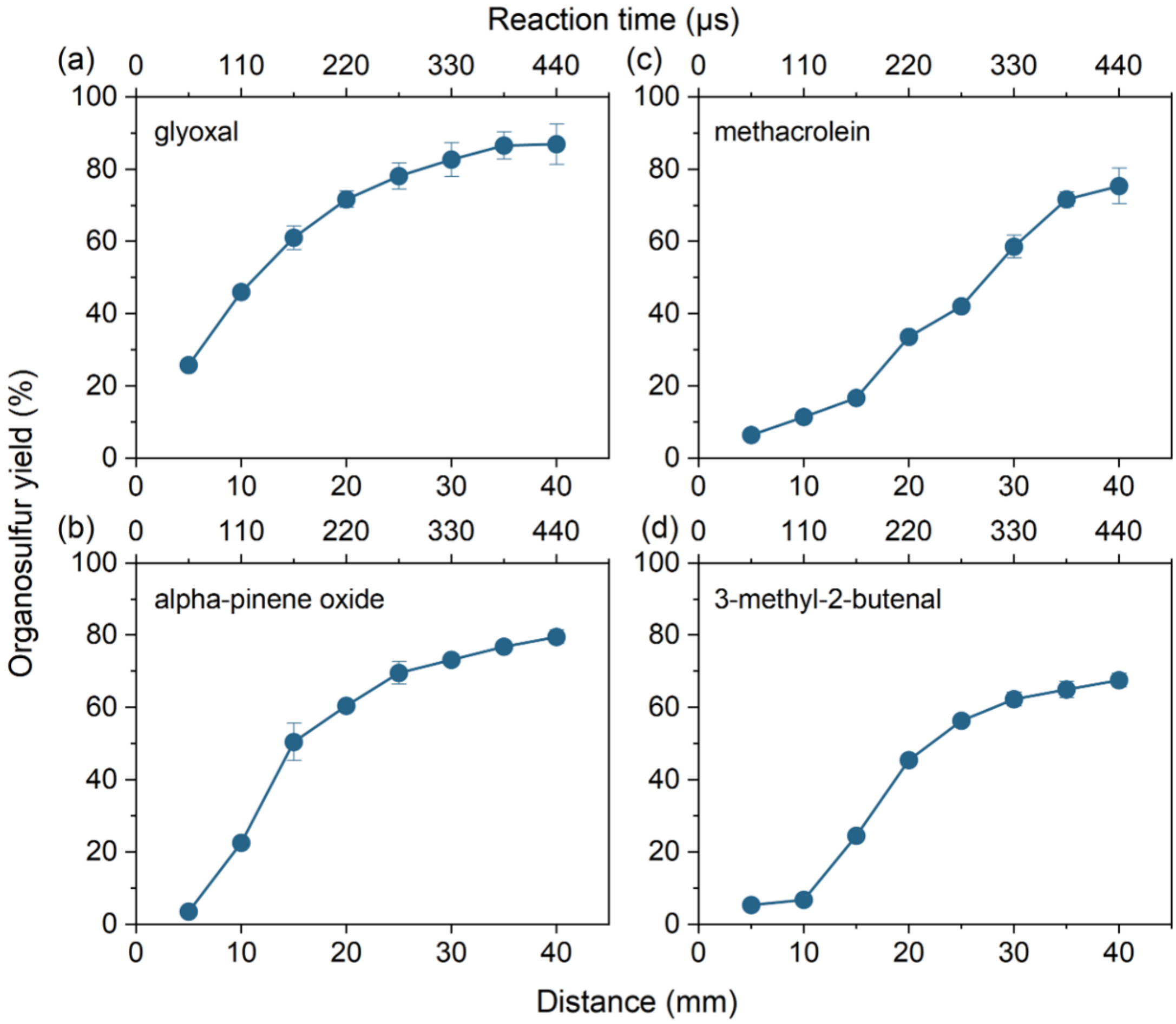

Figure S16. Organosulfur yield as a function of the reaction distance and reaction time. (a) Na_2SO_3 (100 μM) and glyoxal (300 μM). (b) Na_2SO_3 (100 μM) and alpha-pinene oxide (700 μM). (c) Na_2SO_3 (100 μM) and methacrolein (100 μM). (d) Na_2SO_3 (100 μM) and 3-methyl-2-butenal (30 μM). Error bars (mean ± standard deviation (SD), n = 3).

.

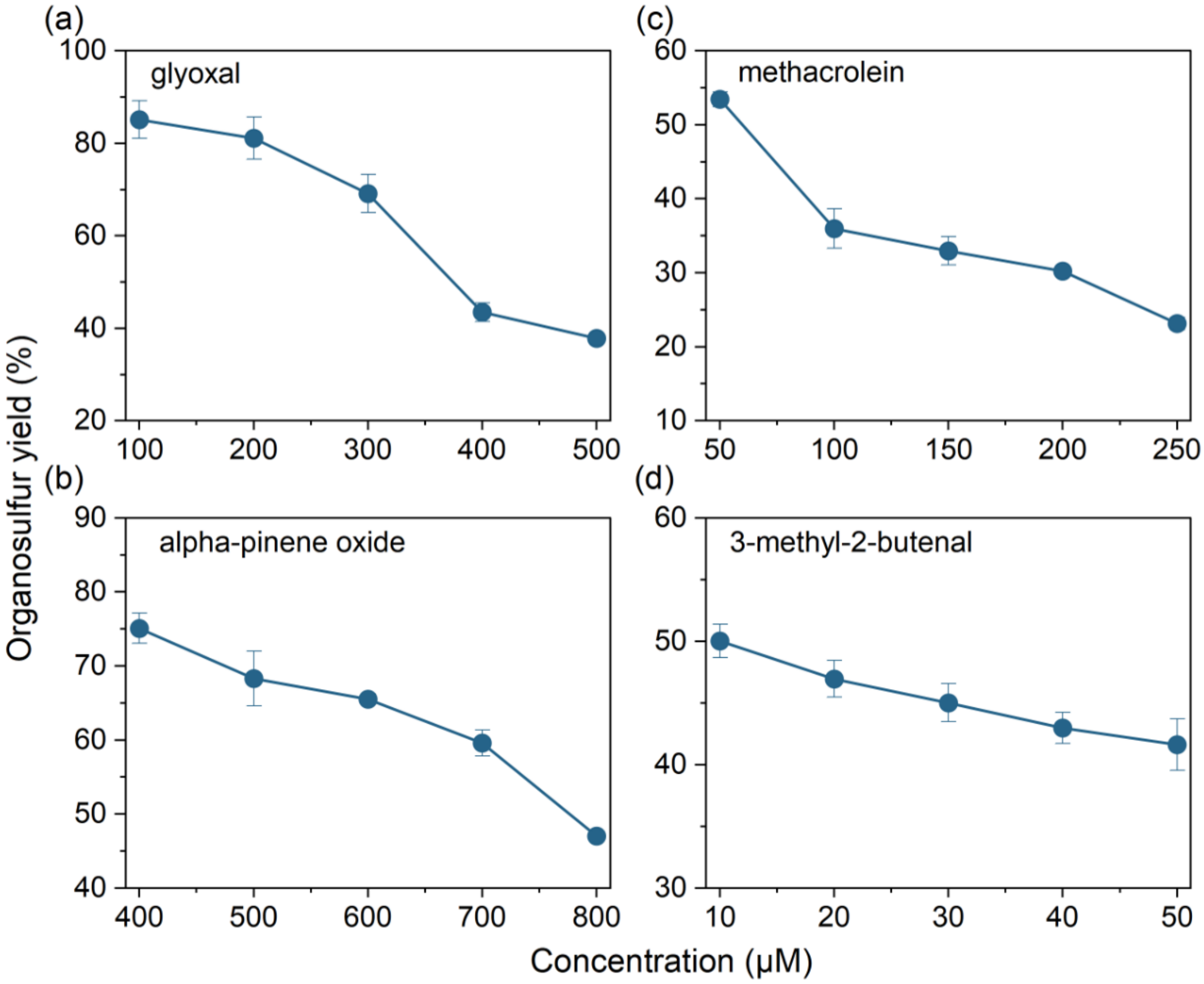

Figure S17. Organosulfur yield as a function of the oxygenated volatile organic compounds (OVOCs) concentration at a Na_2SO_3 concentration of 100 μM and a reaction distance of 20 mm. (a) Glyoxal. (b) Alpha-pinene oxide. (c) Methacrolein. (d) 3-methyl-2-butenal. Error bars (mean ± standard deviation (SD), n = 3).

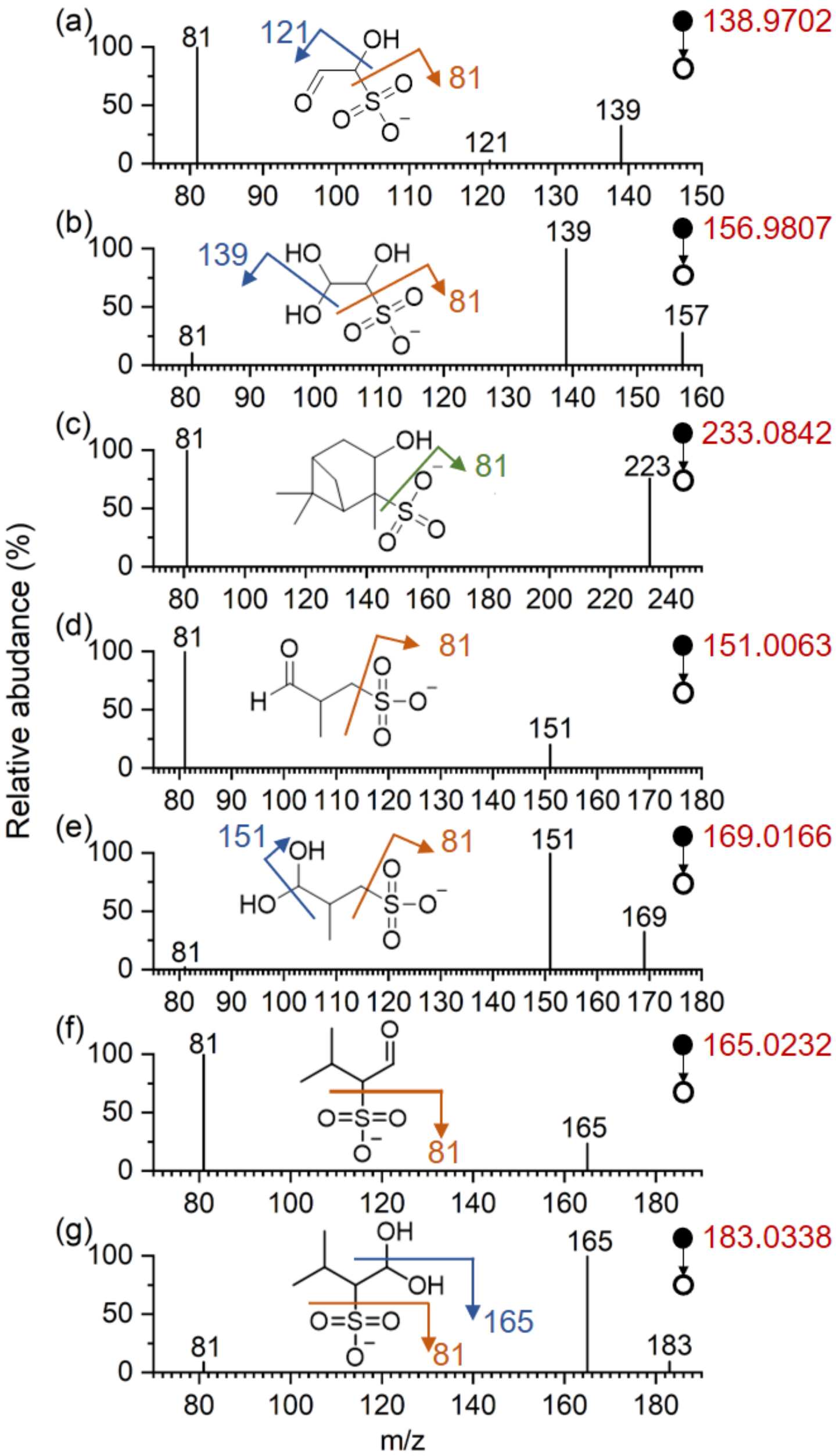

.

Figure S18. Tandem mass spectrometry (MS/MS) spectra of organosulfur compounds detected in ambient aerosol samples collected from Tianjin. (a) Mass-to-charge ratio (m/z) 138.9702. (b) m/z 156.9807. (c) m/z 233.0842. (d) m/z 151.0063. (e) m/z 169.0166. (f) m/z 165.0232. (g) m/z 183.0338.
