# Supplementary material for: Rapid spontaneous generation of organosulfur from inorganic sulfur in atmospheric microdroplets
Source: Nat Commun. 2026 Sep 10;17:10684. doi: 10.1038/s41467-026-77473-5 (PMC13655161; doi:10.1038/s41467-026-77473-5)
Supplement: Supplementary file 2 [file 41467_2026_77473_MOESM2_ESM.pdf]

nature portfolio

Peer Review File

Rapid Spontaneous Generation of Organosulfur from Inorganic Sulfur in Atmospheric Microdroplets

Corresponding Author: Professor Pingqing Fu

This file contains all reviewer reports in order by version, followed by all author rebuttals in order by version.

Version 0:

Reviewer comments:

Reviewer #1

(Remarks to the Author)
General comments:
This manuscript entitled "Rapid Spontaneous Generation of Organosulfur from Inorganic Sulfur in Atmospheric Microdroplets" reports a laboratory study on the ultrafast conversion of inorganic sulfur (S(IV)) into organosulfur compounds via reactions with OVOCs in microdroplets. Using a nebulization–mass spectrometry setup, the authors demonstrate that a variety of organosulfur species can form within microseconds without the need for catalysts, radiation, or external oxidants. The study further proposes that strong interfacial electric fields drive the spontaneous generation of reactive radicals (e.g., $SO_3^{\bullet-}$, •OH), enabling both nucleophilic and radical addition pathways. Importantly, several laboratory-identified products are also observed in ambient aerosol samples, suggesting potential atmospheric relevance.
The topic is timely and relevant, particularly given the growing interest in microdroplet chemistry and its implications for atmospheric processes. The proposed mechanism—linking interfacial electric field effects with rapid organosulfur formation—is intriguing and could represent a meaningful advance in understanding missing sources of organosulfur in atmospheric models. Overall, the manuscript is of high interest, and after minor revisions, it would be suitable for publication.

Specific comments:
1. Mechanistic interpretation and role of the electric field
The central claim that strong interfacial electric fields drive one-electron oxidation of SO32- to SO3•- requires further substantiation. In the current form, the electric field is not directly measured but inferred from literature, and alternative pathways (e.g., O2-mediated oxidation, trace impurities, or ionization-induced processes) are not fully excluded. A more critical discussion of competing mechanisms would strengthen the mechanistic interpretation.

2. Distinguishing microdroplet chemistry from MS/inlet effects
The assumption that reactions occur exclusively during the microdroplet flight (~220 μs) is not fully demonstrated. Given the high inlet temperature (350 °C) and ionization conditions, potential contributions from inlet-induced reactions or fragmentation cannot be ruled out. Additional evidence supporting in-flight reaction dominance would improve confidence in the conclusions.

3. Kinetics and quantitative constraints
Although the manuscript emphasizes ultrafast reaction timescales, the analysis remains largely qualitative. No rate constants, yields, or concentration-dependent trends are provided, making it difficult to assess the efficiency and atmospheric significance of the proposed pathway. Incorporating even semi-quantitative analysis would substantially enhance the rigor.

4. Product identification and structural certainty
Product assignments are primarily based on accurate mass and MS/MS fragmentation. However, these techniques provide molecular formulas rather than definitive structures, and potential isomeric contributions (especially for α-pinene oxide systems) remain unresolved. The level of structural certainty should be more clearly stated, and associated uncertainties discussed.

5. Role of oxidants and reaction pathways
The involvement of •OH is inferred through TEMPO trapping, but its relative contribution compared to SO3•- and other

oxidants is not quantified. Clarifying the relative importance of radical versus nucleophilic pathways under the experimental conditions would improve the mechanistic clarity.

6. Comparison between bulk and microdroplet systems
The comparison with bulk-phase experiments highlights strong differences in reactivity, but the conditions (e.g., concentration, oxidant availability, mixing) are not directly comparable. This limitation should be more explicitly acknowledged when drawing conclusions about rate enhancement.

7. Minor points
a) Descriptive terms such as “rapid”, “remarkable”, and “spontaneous” are used frequently and could be replaced or supported by quantitative metrics.
b) Figures, particularly mass spectra, would benefit from clearer annotation and discussion of uncertainty or reproducibility.

Reviewer #2

(Remarks to the Author)
In this manuscript, the authors report a novel finding that inorganic sulfur can spontaneously and rapidly convert into organosulfur compounds within atmospheric microdroplets at the air-water interface. The reaction proceeds within approximately 220 microseconds without any catalyst, external potential, or radiation, driven by the strong interfacial electric field of microdroplets. The authors verify the formation pathways via nucleophilic addition and radical addition, and further confirm the existence of these organosulfur products in ambient aerosols from urban Tianjin and high-altitude Shanghuang Station. However, lacking originality in the organosulfur formation pathway, the core reaction types, radical-driven logic, and product assignment belong to extensions of existing mechanisms for atmospheric organosulfur formation (Environ. Sci. Technol. 2025, 59, 16556–16566). No novel reaction mechanism, key intermediate, or driving factor is identified, resulting in limited scientific breakthrough. I suggest this article cannot be published on Nature Communications at present.

Questions:
P6 line 91 & P7 line 120: Laboratory concentrations of Na2SO3 (40–100 μM) are used. How do these values compare to ambient sulfite levels in atmospheric droplets, and how can the results be extrapolated to real atmospheric conditions?
P8–P11 (related to Figure 2): The description of Core MS/MS fragment evidence for key products is too simplistic, and the secondary mass spectrometry data is insufficient. The actual sample also requires corresponding secondary mass spectrometry data, and an explanation is needed as to why only sulfate fragments are present after the cleavage and no fragments from other parts are observed.
P12 line 207–211: Bulk control experiment only used methacrolein and Na2SO3 for 3 days. Were bulk experiments also performed for glyoxal, alpha pinene oxide, and 3 methyl 2 butenal to confirm no organosulfur formation?
P13 The reaction pathway is determined solely based on mass spectrometry data and speculation. At the very least, theoretical calculations are required to validate the speculation.
P15 line 260–261: Where is the glyoxal product at m/z 138.9702 confirmed in real samples?
P16 line 272–277: The absence of m/z 169.0176 and m/z 183.0333 in Shanghuang samples is attributed to larger droplets and weaker interfacial electric field. Is there field data (droplet size distribution) to quantitatively support this explanation? Why is it not the case that the corresponding volatile organic compound emissions from this observatory are low? This speculation requires more reasonable verification rather than mere speculation.

Reviewer #3

(Remarks to the Author)
The manuscript reports the rapid and spontaneous formation of organosulfur compounds from inorganic sulfite and representative oxygenated volatile organic compounds in aqueous microdroplets. This is an interesting and timely study that addresses an important question in atmospheric chemistry related to the air–water interface of aqueous aerosols. The experimental observations are potentially significant, and the combination of laboratory microdroplet experiments with ambient aerosol measurements strengthens the environmental relevance of the work. Overall, the manuscript is clearly organized, and the results generally support the proposed formation pathways based on the current experimental setup. These findings have significant implications for understanding organosulfur formation in atmospheric aqueous aerosols. I recommend minor revision with the following suggestions for the authors.

1. The order of the paragraphs in the Introduction could be adjusted to better highlight the importance of this study in the context of atmospheric chemistry.
2. For the statement, “In particular, the potential contributions of interfacial or microdroplet-mediated processes to organosulfur formation have rarely been explored,” relevant references should be added to better clarify how this work builds upon previous studies.
3. The reaction conditions, such as RH and temperature, as well as solution properties, such as solution pH, should be clearly specified.
4. The estimated reaction time of approximately 220 μs is central to the claim of ultrafast organosulfur formation. Since this value is based on previous studies, a brief discussion of the uncertainty and limitations of this estimate would be helpful. If available, reaction-distance-dependent data would further strengthen this claim.
5. In line 70, the authors propose that inorganic S(IV) can undergo spontaneous oxidation and subsequently react with OVOCs to produce organosulfur compounds. It would be helpful if the authors could present full-scan mass spectra,

especially including the low-m/z region, to more clearly demonstrate the formation of sulfate-related species.
6. The manuscript would benefit from a clearer description of the bulk-phase control experiments. In particular, the authors should specify whether similar bulk controls were performed for all OVOC systems. This clarification would strengthen the conclusion that the observed organosulfur formation is specific to the microdroplet environment.
7. For Figure 2, the mechanistic assignments are generally reasonable, but some proposed pathways would benefit from additional discussion or supporting evidence. For example, regarding the formation of $C_2H_5O_6S^-$, $C_4H_9O_5S^-$, and $C_5H_{11}O_5S^-$, it would be helpful to clarify whether the current evidence can distinguish •OH-initiated reactions at the air–water interface from possible hydrolysis-related pathways. If not, the proposed mechanisms should be described more cautiously.
8. The discussion of the differences between the Tianjin and Shanghuang aerosol samples is interesting. The authors attribute these differences to larger droplet sizes under typically higher relative humidity conditions. Relevant observational data from these two sites would further support this explanation. Otherwise, I suggest that the authors use more cautious wording.

Version 1:

Reviewer comments:

Reviewer #1

(Remarks to the Author)
no further comments

Reviewer #2

(Remarks to the Author)
The authors are commended for their extensive efforts in addressing the prior reviewer comments, including the addition of substantial experimental data, theoretical calculations, and targeted textual revisions. The newly added bulk-phase control experiments covering all OVOC systems, complementary ATR-FTIR and UPLC-MS characterizations, quantum chemical calculations and metadynamics simulations, as well as MS/MS verification of ambient aerosol samples, have markedly improved the comprehensiveness and evidentiary strength of the work. However, several issues remain to be clarified.

1.The supplementary sheath gas pressure-dependent experiment only establishes a positive correlation between decreased droplet size and elevated $SO3^{•-}$ signal intensity. Critically, droplet size reduction is inherently accompanied by concentration effects driven by solvent evaporation, as well as enrichment of reactants at the air-water interface. The proposed mechanism requires more targeted verification, or discussion.

2.From a fundamental chemical perspective, free radical reactions are intrinsically fast processes. There is no need to explicitly emphasize that the reaction occurs on the microsecond timescale in certain statements, and the wording can be adjusted accordingly (e.g., Page 11, Line 208). Meanwhile, the authors should discuss the half-life of the relevant radical in both the bulk phase and gas phase. If the radical has an extremely short half-life, the reaction may indeed proceed within microseconds, which is consistent with the low ion intensity of the radical detected by mass spectrometry. Additional discussion on this point is needed to ensure the chemical plausibility of the proposed reaction mechanism.

Overall, the authors have completed a substantial body of supplementary work in this round of revision, which has significantly strengthened the foundational evidence of the study. I recommend acceptance of the manuscript following minor revisions.

Reviewer #3

(Remarks to the Author)
The authors had revised their manuscript carefully, now it is acceptable as it is.

Open Access This Peer Review File is licensed under a Creative Commons Attribution 4.0 International License, which permits use, sharing, adaptation, distribution and reproduction in any medium or format, as long as you give appropriate credit to the original author(s) and the source, provide a link to the Creative Commons license, and indicate if changes were made.
In cases where reviewers are anonymous, credit should be given to 'Anonymous Referee' and the source.
The images or other third party material in this Peer Review File are included in the article's Creative Commons license, unless indicated otherwise in a credit line to the material. If material is not included in the article's Creative Commons license and your intended use is not permitted by statutory regulation or exceeds the permitted use, you will need to obtain permission directly from the copyright holder.
To view a copy of this license, visit https://creativecommons.org/licenses/by/4.0/

Responses to Reviewers

We greatly appreciate the positive comments and the insightful suggestions/comments of the referees about our work. We tried our best to revise and improve the manuscript and made changes in the manuscript accordingly. Our point-by-point responses to the comments are presented below, with the reviewers' comments in italics, our responses in regular text, and the corresponding modification made in the manuscript and Supplementary Information are highlighted in blue.

Response to Reviewer #1

General comments:
This manuscript entitled "Rapid Spontaneous Generation of Organosulfur from Inorganic Sulfur in Atmospheric Microdroplets" reports a laboratory study on the ultrafast conversion of inorganic sulfur (S(IV)) into organosulfur compounds via reactions with OVOCs in microdroplets. Using a nebulization-mass spectrometry setup, the authors demonstrate that a variety of organosulfur species can form within microseconds without the need for catalysts, radiation, or external oxidants. The study further proposes that strong interfacial electric fields drive the spontaneous generation of reactive radicals (e.g., $SO_3^{\bullet -}$*, •OH), enabling both nucleophilic and radical addition pathways. Importantly, several laboratory-identified products are also observed in ambient aerosol samples, suggesting potential atmospheric relevance.*
The topic is timely and relevant, particularly given the growing interest in microdroplet chemistry and its implications for atmospheric processes. The proposed mechanism—linking interfacial electric field effects with rapid organosulfur formation—is intriguing and could represent a meaningful advance in understanding missing sources of organosulfur in atmospheric models. Overall, the manuscript is of high interest, and after minor revisions, it would be suitable for publication.

> **RE:** We are grateful to this reviewer's positive comments. We have carefully addressed all your constructive suggestions to further improve the manuscript. The information has been presented in the revised manuscript based on the reviewer's suggestions.

Specific comments:
1. Mechanistic interpretation and role of the electric field
The central claim that strong interfacial electric fields drive one-electron oxidation of SO_3^{2-} *to* $SO_3^{-\bullet}$ *requires further substantiation. In the current form, the electric field is*

not directly measured but inferred from literature, and alternative pathways (e.g., O_2-mediated oxidation, trace impurities, or ionization-induced processes) are not fully excluded. A more critical discussion of competing mechanisms would strengthen the mechanistic interpretation.

RE: We thank the reviewer for this insightful comment. The oxidation of SO_3^{2-} to $SO_3^{-\bullet}$ could primarily arise from several factors, including strong interfacial electric fields, dissolved O_2, trace transition metals in solution, or ionization-induced processes during MS analysis. To identify the dominant mechanism, we conducted additional control experiments. As detailed in the revised manuscript (page 6-7, line 104-123), our results exclude dissolved O_2, trace transition metals, and ionization-induced processes as dominant pathways. In addition, pressure-dependent experiments indicate that $SO_3^{-\bullet}$ formation is promoted by interfacial electric fields in water microdroplets. Although the interfacial electric field was not directly measured in this work, it can facilitate single electron transfer have been extensively reported (Lee et al., *Proc. Natl. Acad. Sci. U. S. A.* 2019, 116, 19294-19298.; Gong et al., *J. Am. Chem. Soc.* 2022, 144, 3510-3516.).

Page 6-7, Line 104-123, We have added the following discussion:

"The oxidation of SO_3^{2-} to $SO_3^{-\bullet}$ could potentially arise from several other factors, including dissolved oxygen, trace transition metals in solution, or ionization-induced processes in the mass spectrometer. To evaluate these possibilities, a series of control experiments were conducted. Reducing the dissolved oxygen concentration from 7.07 to 2.43 mg L^{-1} by boiling ultrapure water had no significant effect on the $SO_3^{-\bullet}$ signal intensity. Additionally, inductively coupled plasma tandem mass spectrometry (ICP-MS) analysis showed that trace metal concentrations in ultrapure, deionized, and distilled water were all below 0.1 ppb (Table S1), and no significant differences in $SO_3^{-\bullet}$ production were observed among microdroplet experiments performed with these different water matrices. Furthermore, $SO_3^{-\bullet}$ was not detected when a Na_2SO_3 solution was analyzed using the same mass spectrometer operated with a conventional electrospray ionization (ESI) source, and no external voltage was applied during the microdroplet experiments. Taken together, these results rule out dissolved oxygen, trace transition metals, and ionization-induced processes as major contributors to $SO_3^{-\bullet}$ formation.

To further investigate the role of the interfacial electric field in $SO_3^{-\bullet}$ formation, pressure-dependent experiments were conducted. Previous studies have shown that increasing the sheath gas pressure from 40 to 120 psi reduces droplet diameter from approximately 20 to 4 μm, thereby enhancing the interfacial electric field due to increased surface curvature.[32,39] As shown in Figure S2, the $SO_3^{-\bullet}$ signal intensity increased with increasing sheath gas pressure from 40 to 120 psi. This trend supports the hypothesis that interfacial electric fields in microdroplets play a key role in promoting $SO_3^{-\bullet}$ formation."

Table S1. Concentrations of trace metals in different water matrices determined by ICP-MS.

Sample	Ti	V	Cr	Mn	Fe	Co	Ni	Cu	Y	Mo
Ultrapure water	0.016	<0.000	0.006	<0.000	0.089	0.007	<0.000	0.009	<0.000	<0.000
Deionized water	<0.000	<0.000	<0.000	<0.000	<0.000	0.0	0.0	<0.000	<0.000	<0.000
Distilled water	<0.000	<0.000	0.0	<0.000	<0.000	<0.000	0.001	0.001	<0.000	<0.000

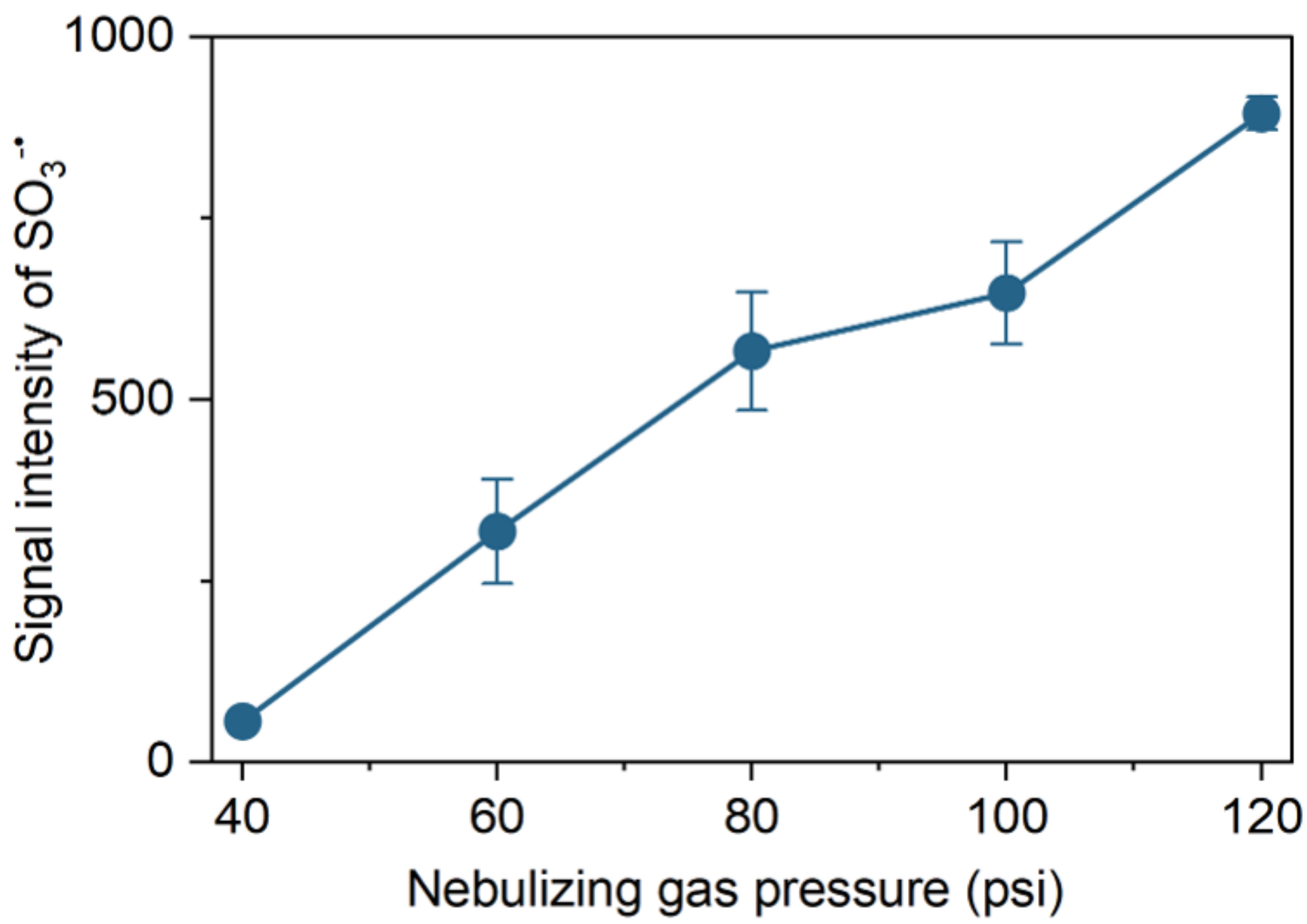

Figure S2. Effect of sheath gas pressure on $SO_3^{-\bullet}$ signal intensity in microdroplets generated from a 40 μM Na_2SO_3 solution at a reaction distance of 20 mm.

2. Distinguishing microdroplet chemistry from MS/inlet effects
The assumption that reactions occur exclusively during the microdroplet flight (~220 μs) is not fully demonstrated. Given the high inlet temperature (350 ℃) and ionization conditions, potential contributions from inlet-induced reactions or fragmentation cannot be ruled out. Additional evidence supporting in-flight reaction dominance would improve confidence in the conclusions.

RE: We thank the reviewer for raising this important point. We performed distance-dependent measurements and found that yields of products increase with

reaction distance (see revised manuscript, Line 254-260), consistently supporting that product formation occurs during microdroplet flight.
Furthermore, we found that lowering the mass spectrometer inlet temperature from 350 °C to 40 °C had no significant effect on product yields. Moreover, ATR-FTIR spectra showed characteristic functional groups of organosulfur products (Line 229-237). These observations collectively rule out the possibility of inlet-induced reactions or thermal fragmentation and further support that the reaction occurs within the flying microdroplets.

Page 13, Line 254-260, We have added the following discussion:

> "By adjusting the reaction distance from 5 to 40 mm, the corresponding reaction time was estimated to range from 55 to 495 μs. A clear increase in product yield was observed with increasing distance (Figure S16). The average yield of these products reached up to 50% when the reaction distance is 20 mm. These results indicate that organosulfur formation occurs during the microsecond timescale flight of microdroplets rather than within the mass spectrometer inlet region, which is consistent with previous studies.[45,65]"

Page 12, Line 229-237, We have added the following discussion:

> "For structural identification, microdroplets were collected for 1 h and subsequently characterized by attenuated total reflectance Fourier-transform infrared (ATR-FTIR) spectroscopy. As shown in Figure S13, the characteristic strong absorption bands at 1193 and 1050 cm^{-1} are assigned to the antisymmetric and symmetric stretching vibrations of the sulfonate group, respectively.[63] In addition, the signal observed near 621 cm^{-1} is attributed to C-S-O stretching vibrations, and the broad absorption centered at approximately 3327 cm^{-1} is assigned to O-H stretching vibrations.[64] These spectral features are consistent with previous reports and provide complementary spectroscopic evidence for the formation of organosulfur compounds in microdroplets."

Page 25, Line 464-470, we have added the analysis method of ATR-FTIR in the revised manuscript in Method Section as follows:

> "**ATR-FTIR and UPLC-MS analysis.** For structural characterization, microdroplets were collected into a 2 mL brown glass vials (recombusted at 450°C, 6 h) over a period of 1 h. The organosulfur products formed in the microdroplets were analyzed using attenuated total reflectance Fourier-transform infrared spectroscopy (ATR-FTIR; Bruker Optics, Ettlingen, Germany). Spectra were recorded over the range of 4000-400 cm^{-1} with a resolution of 4 cm^{-1} accumulating 32 scans per sample. The background absorption of methanol was subtracted to obtain the final infrared spectra."

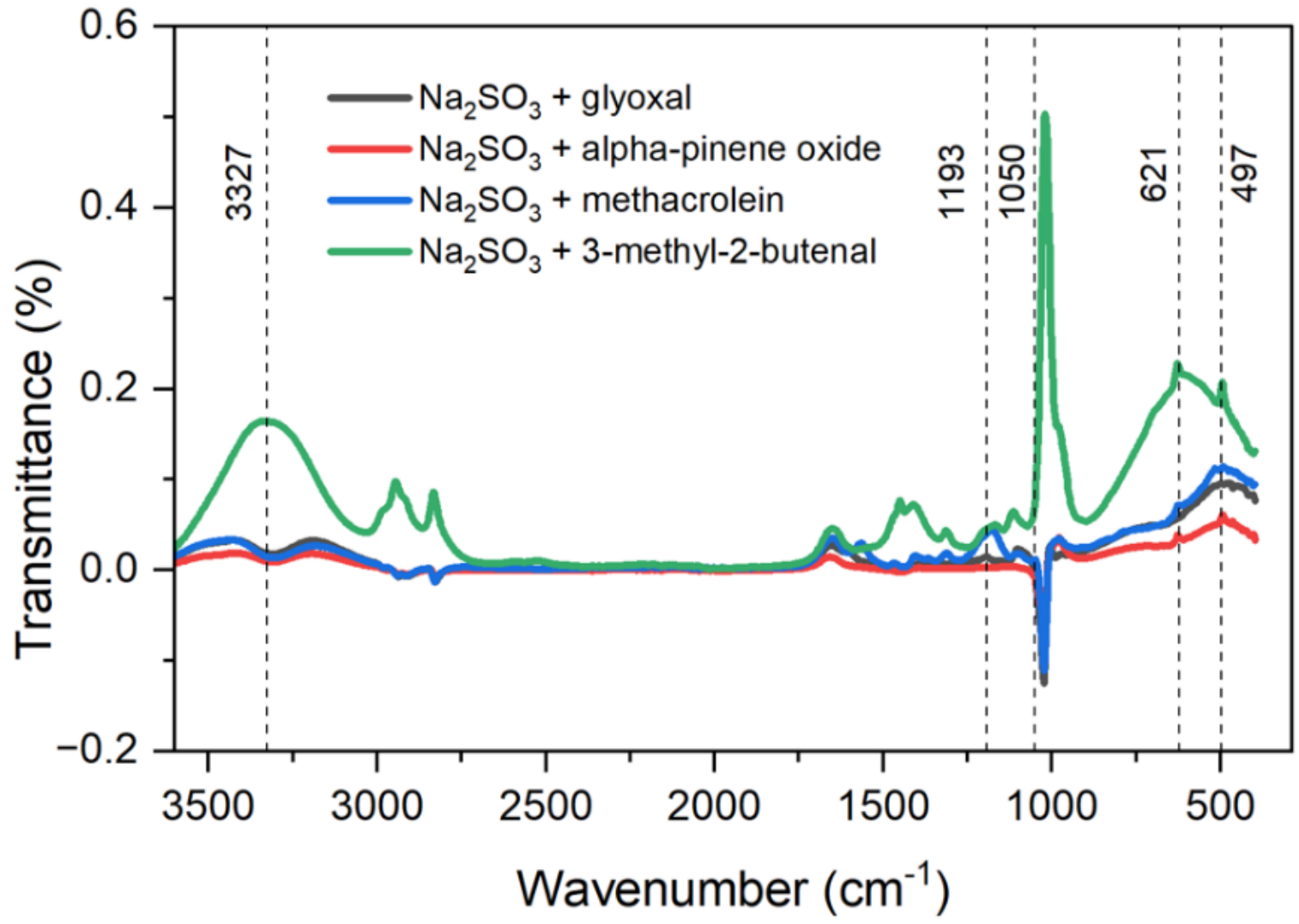

Figure S13. ATR-FTIR spectrum of the organosulfur products collected from microdroplet reactions of Na_2SO_3 with representative OVOCs.

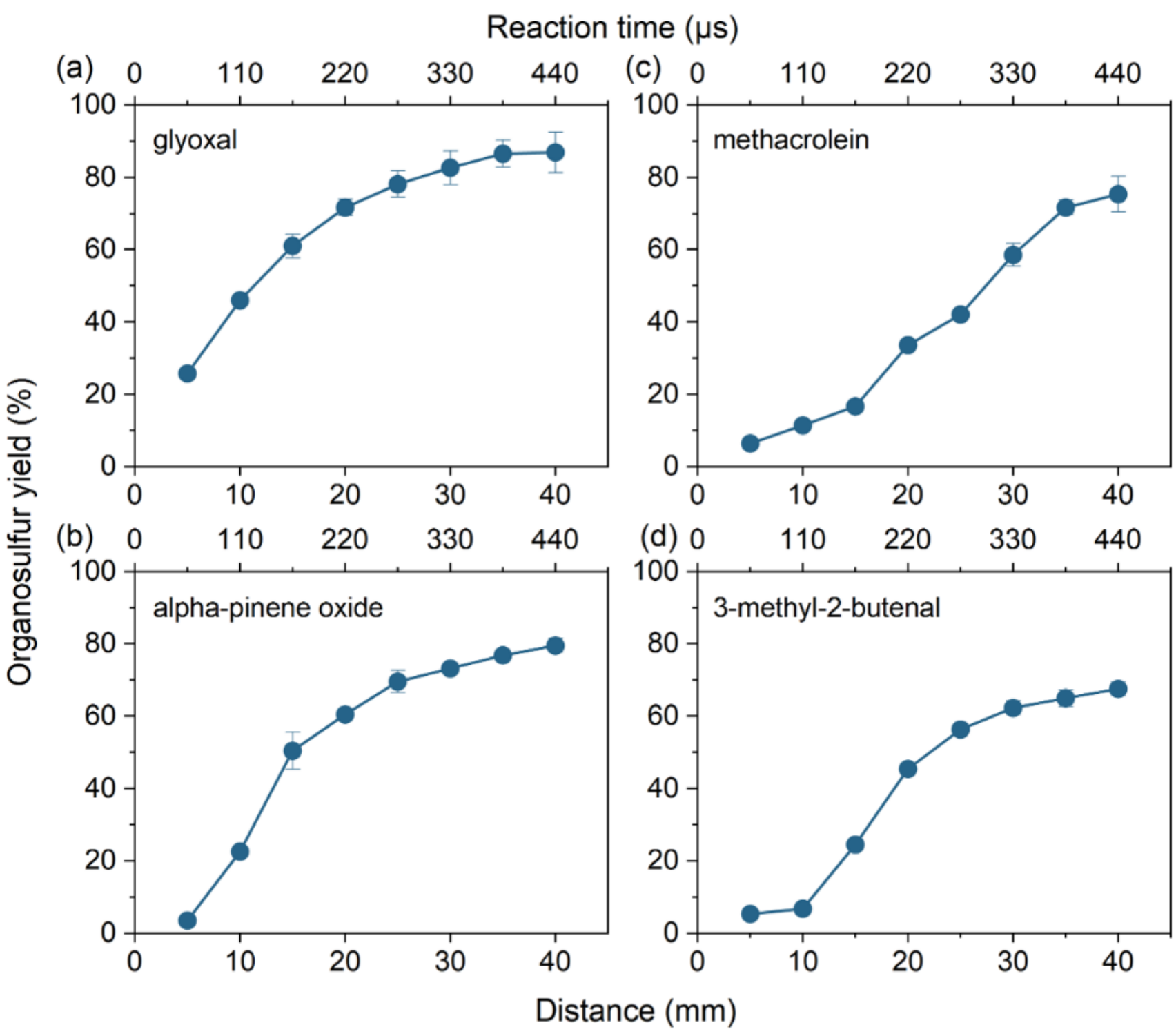

Figure S16. Organosulfur yield as a function of the reaction distance and reaction time.

3. Kinetics and quantitative constraints

Although the manuscript emphasizes ultrafast reaction timescales, the analysis remains largely qualitative. No rate constants, yields, or concentration-dependent trends are provided, making it difficult to assess the efficiency and atmospheric significance of the proposed pathway. Incorporating even would substantially enhance the rigor.

RE: We thank the reviewer for this insightful comment. Semi-quantitative analysis was performed using hydroxymethanesulfonate (HMS) as an internal standard. We conducted concentration-dependent experiments and found that product yields decreased with increasing OVOC concentrations. This trend may be attributed to the greater fraction of molecules residing at the microdroplet surface at lower concentrations, further supporting the key role of the air-water interface in promoting these reactions (see revised manuscript, Line 260-263). Moreover, the high yields (up to 80%) observed in these microdroplet systems highlight the efficiency of interfacial organosulfur formation and support its potential importance as an atmospheric source of organosulfur compounds.

Page 14, Line 260-263, We have added corresponding discussion as follows:

"In addition, concentration-dependent experiments revealed that organosulfur product yields decreased with increasing OVOC concentrations (Figure S17). This trend may be attributed to interfacial effects, as lower bulk concentrations increase the relative fraction of reactants partitioned to the air-water interface of microdroplets."

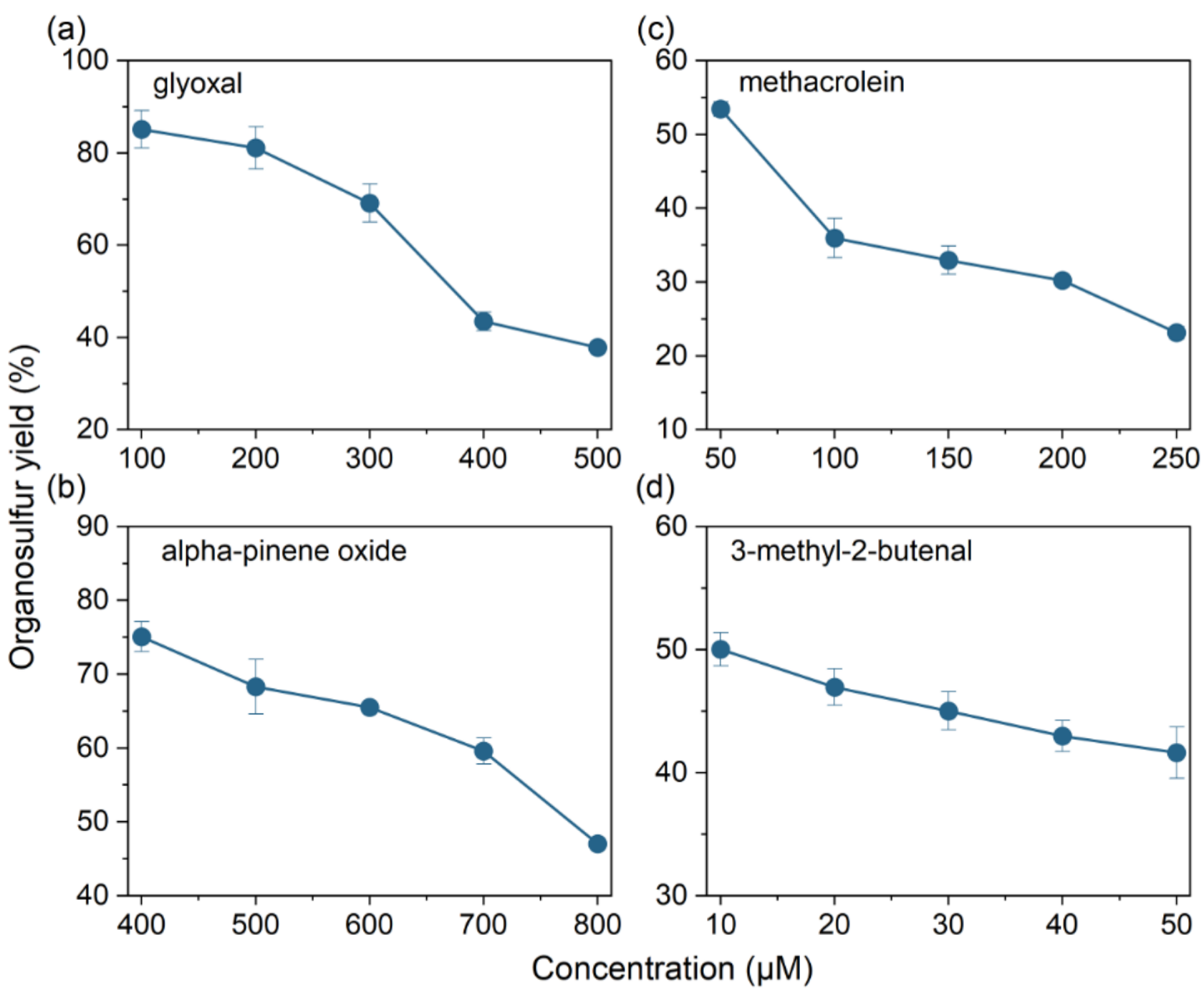

Figure S17. Organosulfur yield as a function of the OVOCs concentration when the reaction distance is 20 mm.

4. Product identification and structural certainty
Product assignments are primarily based on accurate mass and MS/MS fragmentation. However, these techniques provide molecular formulas rather than definitive structures, and potential isomeric contributions (especially for α-pinene oxide systems) remain

unresolved. The level of structural certainty should be more clearly stated, and associated uncertainties discussed.

RE: We thank the reviewer for this insightful comment. To strengthen structural assignments, additional ATR-FTIR and UPLC-MS analyses were conducted. ATR-FTIR spectra showed characteristic -SO_3 and S-O/C-S-O vibrational bands, providing independent evidence for the formation of C-S bond in organosulfur products. The UPLC-MS results indicate that the alpha-pinene oxide system may contain multiple isomeric products, as the extracted ion chromatogram of *m/z* 233.0842 exhibited three distinct chromatographic peaks. In contrast, the remaining products each exhibited a single chromatographic peak, suggesting that no major isomeric contributions were resolved under the present chromatographic conditions. Together with the accurate-mass and MS/MS data, these complementary analyses provide additional support for the proposed product structures.

However, UPLC-MS can only distinguish constitutional and positional isomers. In the absence of commercially available pure standards for these organosulfur products, assigning the exact stereochemical configuration to each chromatographic peak remains challenging. More precise structural elucidation of these organosulfur isomers remains to be investigated in future work.

Page 12-13, Line 229-252, We have added corresponding discussion as follows:

"For structural identification, microdroplets were collected for 1 h and subsequently characterized by attenuated total reflectance Fourier-transform infrared (ATR-FTIR) spectroscopy. As shown in Figure S13, the characteristic strong absorption bands at 1193 and 1050 cm^{-1} are assigned to the antisymmetric and symmetric stretching vibrations of the sulfonate group, respectively.[63] In addition, the signal observed near 621 cm^{-1} is attributed to C-S-O stretching vibrations, and the broad absorption centered at approximately 3327 cm^{-1} is assigned to O-H stretching vibrations.[64] These spectral features are consistent with previous reports and provide complementary spectroscopic evidence for the formation of organosulfur compounds in microdroplets.

Furthermore, ultra-high performance liquid chromatography-mass spectrometry (UPLC-MS) was utilized to evaluate potential constitutional and positional isomers. As shown in the extracted ion chromatograms (EICs) (Figure S14), *m/z* 138.9702, *m/z* 156.9807, *m/z* 151.0063, *m/z* 169.0166, *m/z* 165.0232, and *m/z* 183.0338 each exhibited a single chromatographic peak, indicating that no significant isomeric contributions were detected under the present chromatographic conditions. In contrast, the EIC of *m/z* 233.0842, generated from the reaction of Na_2SO_3 and alpha-pinene oxide, displayed three distinct chromatographic peaks at retention times of 4.861, 5.084, and 5.443 min, suggesting the possible presence of multiple constitutional and/or positional isomers. The proposed structures of *m/z* 233.0842 are shown in Figure S15. Compounds 1 and 2

are tentatively assigned to sulfonate adducts formed through nucleophilic ring-opening of alpha-pinene oxide by HSO_3^-, whereas compound 3 is proposed to originate from nucleophilic addition of HSO_3^- to C=O of campholenic aldehyde, a rearrangement product generated through acid-catalyzed isomerization of alpha-pinene oxide in microdroplets. However, without commercially available standards for these organosulfur products, assigning the exact stereochemical configuration to each specific chromatogram peak remains a limitation."

Page 25, Line 464-479, we have added the analysis method of ATR-FTIR and HPLC-Q-TOF MS in the revised manuscript in Method Section as follows:

"**ATR-FTIR and UPLC-MS analysis.** For structural characterization, microdroplets were collected into a 2 mL brown glass vials (recombusted at 450°C, 6 h) over a period of 1 h. The organosulfur products formed in the microdroplets were analyzed using attenuated total reflectance Fourier-transform infrared spectroscopy (ATR-FTIR; Bruker Optics, Ettlingen, Germany). Spectra were recorded over the range of 4000-400 cm^{-1} with a resolution of 4 cm^{-1} accumulating 32 scans per sample. The background absorption of methanol was subtracted to obtain the final infrared spectra.
Chromatographic separation was performed using a Shimadzu LC-30A ultra-high performance liquid chromatography (UPLC) system coupled to a Shimadzu Q-TOF mass spectrometer (9030, Kyoto, Japan) equipped with an electrospray ionization (ESI) source in negative ion mode. Separation was performed on an Acquity HSS T3 column (1.8 µm, 2.1 × 100 mm, Waters) maintained at 40 °C. The gradient elution started with 100% mobile phase A (10 mM ammonium acetate) at a flow rate of 0.35 mL/min for 1.7 min, then changed to 100% of mobile phase B (methanol) with a flow rate 0.25 mL/min in 4.7 min and maintained constant for 1 min. The mass spectrometer scanned over *m/z* 50-350 with a scan time of 0.1 s. Data acquisition and processing were performed using LabSolution software."

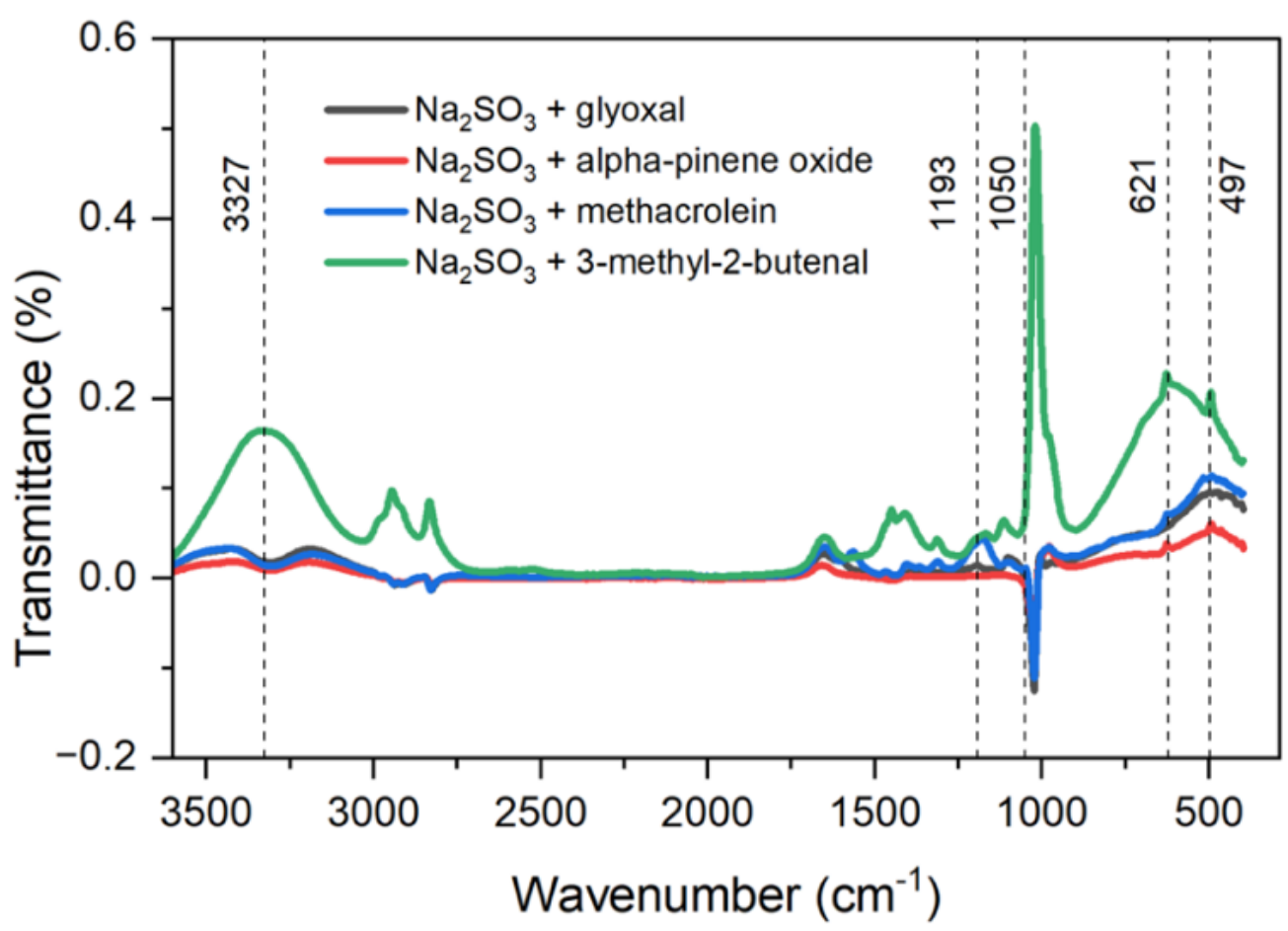

Figure S13. ATR-FTIR spectrum of the organosulfur products collected from microdroplet reactions of Na_2SO_3 with representative OVOCs.

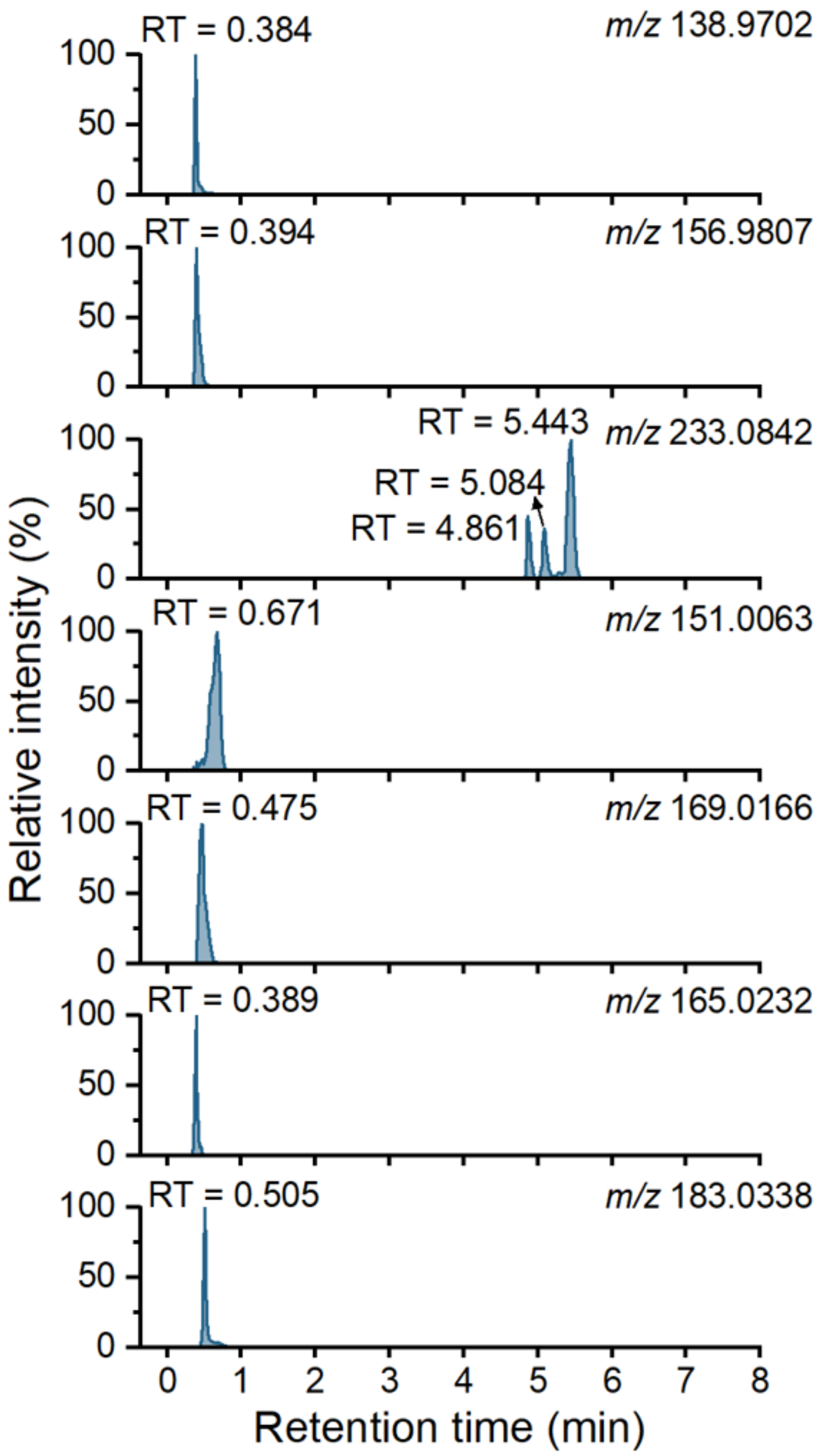

Figure S14. EICs of the organosulfur products generated from reactions of Na_2SO_3 with glyoxal (*m/z* 138.9702, *m/z* 156.9807), alpha-pinene oxide (*m/z* 233.0842), methacrolein (*m/z* 151.0063, *m/z* 169.0166), and 3-methyl-2-butenal (*m/z* 165.0232, *m/z* 183.0338) in water microdroplets.

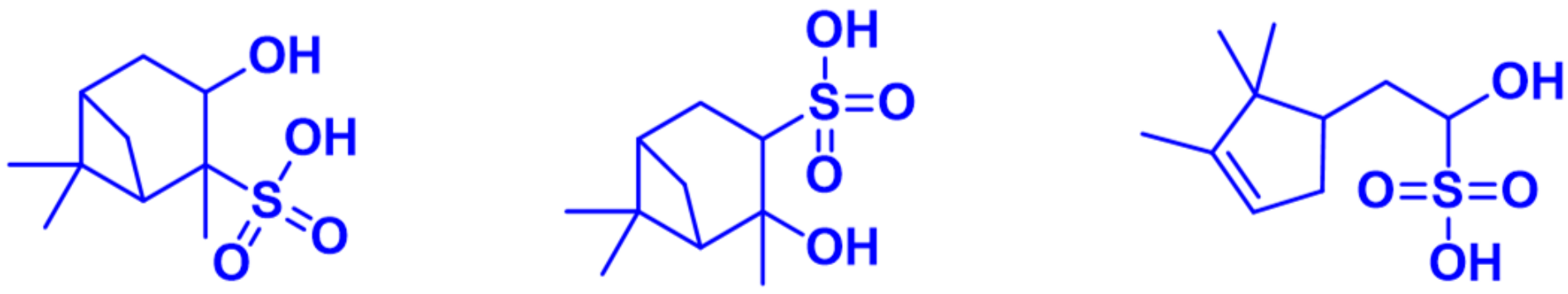

Figure S15. Proposed structures of *m/z* 233.0842 generated from the reaction of alpha-pinene oxide with Na_2SO_3 in water microdroplets.

5. Role of oxidants and reaction pathways

The involvement of •OH is inferred through TEMPO trapping, but its relative contribution compared to $SO_3^{-•}$ and other oxidants is not quantified. Clarifying the relative importance of radical versus nucleophilic pathways under the experimental conditions would improve the mechanistic clarity.

RE: We sincerely thank the reviewer for this insightful suggestion. We agree that the current dataset does not allow quantitative determination of the relative contributions of •OH, $SO_3^{-•}$, and other reactive intermediates. Experimental results from TEMPO and tert-butanol (TBA) scavenging support the involvement of •OH. Meanwhile, $SO_3^{-•}$ was directly detected, and the formation of organosulfur products further supports its participation in subsequent reaction pathways.

Due to the coexistence of multiple reactive intermediates, the individual contributions of radical and nucleophilic pathways cannot be quantitatively distinguished. To further investigate the reaction mechanisms, theoretical calculations were performed, which indicate that both radical and nucleophilic addition pathways are feasible.

Page 12, Line 222-228, We have added corresponding discussion as follows:

"To further evaluate the role of •OH in organosulfur formation, tert-butanol (TBA) was used as a •OH scavenger.[62] As shown in Figure S12, the addition of 50 μM TBA led to substantial decreases in the intensity ratios of $C_2H_5O_6S^-/C_2H_3O_5S^-$, $C_4H_9O_5S^-/C_4H_7O_4S^-$, and $C_5H_{11}O_5S^-/C_5H_9O_4S^-$, which are derived from the reactions of Na_2SO_3 with glyoxal, methacrolein, and 3-methyl-2-butenal, respectively. These results indicate that •OH contributes to the formation of organosulfur products in water microdroplets. Notably, $C_2H_5O_6S^-$, $C_4H_9O_5S^-$, and $C_5H_{11}O_5S^-$ may also be produced through hydrolysis-related pathways."

Page 17-18, Line 313-336, We have added theoretical calculations as follows:

"To further elucidate these mechanisms, quantum chemical calculations and metadynamics simulations based on Born-Oppenheimer molecular dynamics (BOMD)

were performed. Considering that the epoxide moiety of alpha-pinene oxide readily undergoes acid-catalyzed ring opening to form carbonyl-containing intermediates, the theoretical investigations focused on the reactions of HSO_3^- with carbonyl compounds and $SO_3^{-\bullet}$ with unsaturated C=C bonds. The calculations indicate that HSO_3^- reacts with carbonyl compounds through a nucleophilic addition mechanism. The reaction is initiated by C-S bond formation, followed by proton transfer from HSO_3^- to the carbonyl oxygen, mediated by a hydrogen-bonded bridge formed by two interfacial water molecules. The activation free energy at the air-water interface is 11.47 kcal/mol (Figure 4b), which is 7.45 kcal/mol lower than that in bulk solution (Figure 4a), indicating that carbonyl sulfonation is kinetically enhanced in microdroplets. In addition, $SO_3^{-\bullet}$ preferentially adds to the less substituted carbon of the unsaturated C=C bond, with an activation free energy of 4.43 kcal/mol at the air-water interface (Figure 5b), which is 5.47 kcal/mol lower than that in bulk solution (Figure 5a). The reduced reaction energy barrier at the air–water interface is widely attributed to the strong interfacial electric fields in microdroplets.[22,69] In addition, previous studies have demonstrated that external electric fields can further lower reaction barriers and thereby facilitate chemical reactions, highlighting the potential role of interfacial electric fields in modulating microdroplet reactivity.[71,72] In contrast, direct addition of HSO_3^- to the C=C bond exhibits a substantially higher activation barrier (15.13 kcal/ mol) (Figure 5d). These results indicate that alkene sulfonation at the air-water interface proceeds predominantly via an $SO_3^{-\bullet}$-mediated radical pathway rather than direct nucleophilic addition by HSO_3^-. Overall, the theoretical results suggest that interfacial electric fields and interfacial water molecules facilitate the reactions. The combined experimental and computational evidence supports the proposed reaction mechanisms."

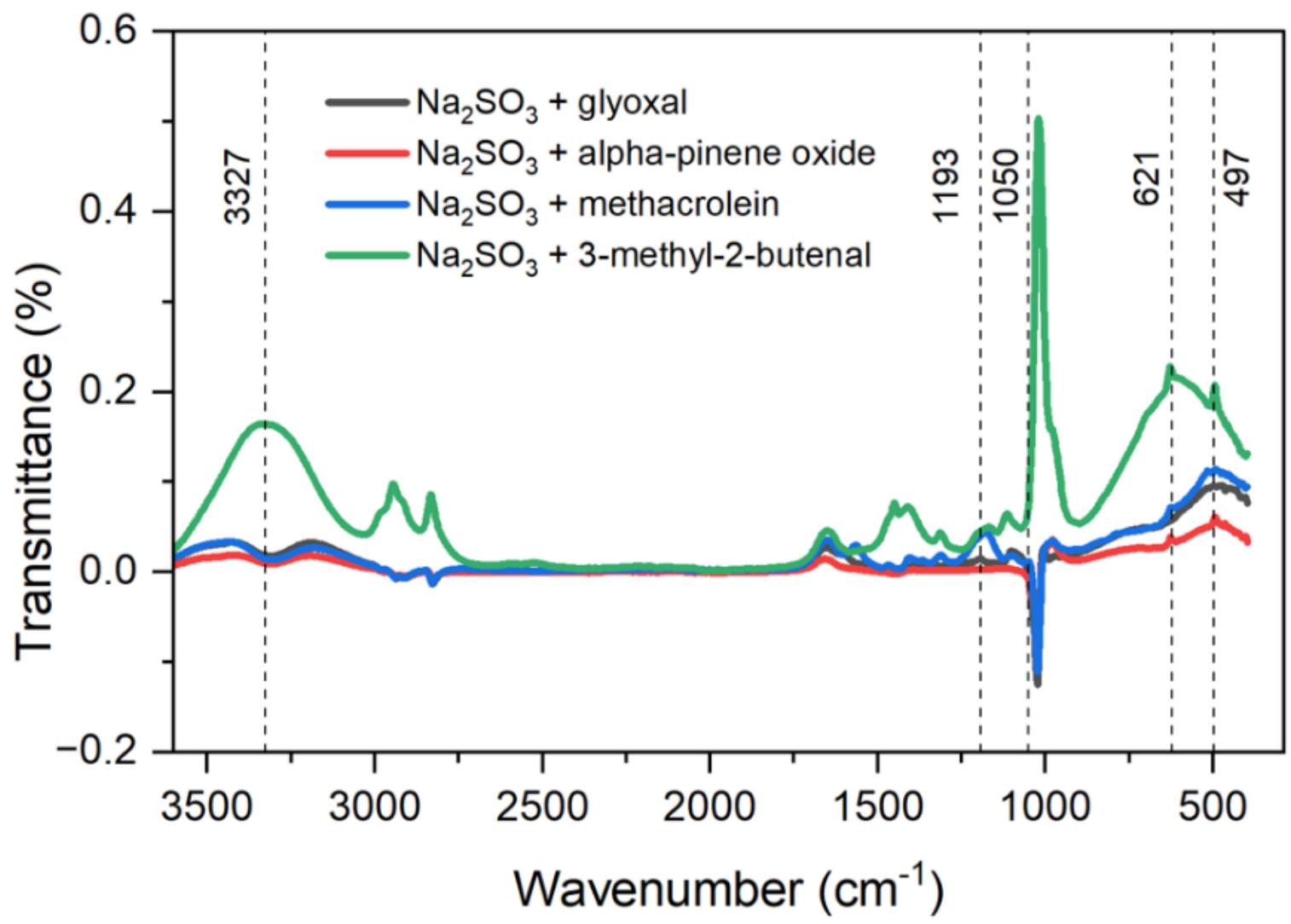

Figure S13. ATR-FTIR spectrum of the organosulfur products collected from microdroplet reactions of Na_2SO_3 with representative OVOCs.

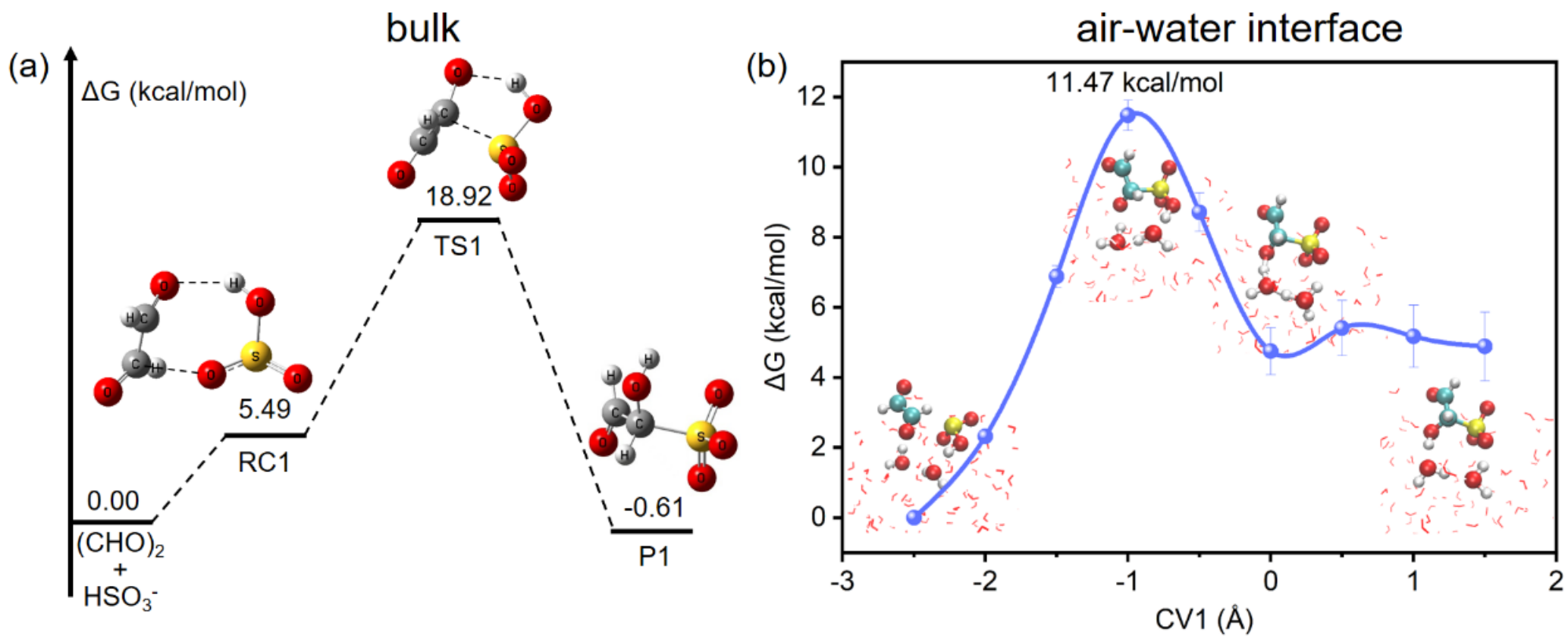

Figure 4. Free energy profiles (ΔG) for the reaction of HSO_3^- with $(CHO)_2$ in the bulk phase (a) and at the air-water interface of microdroplets (b).

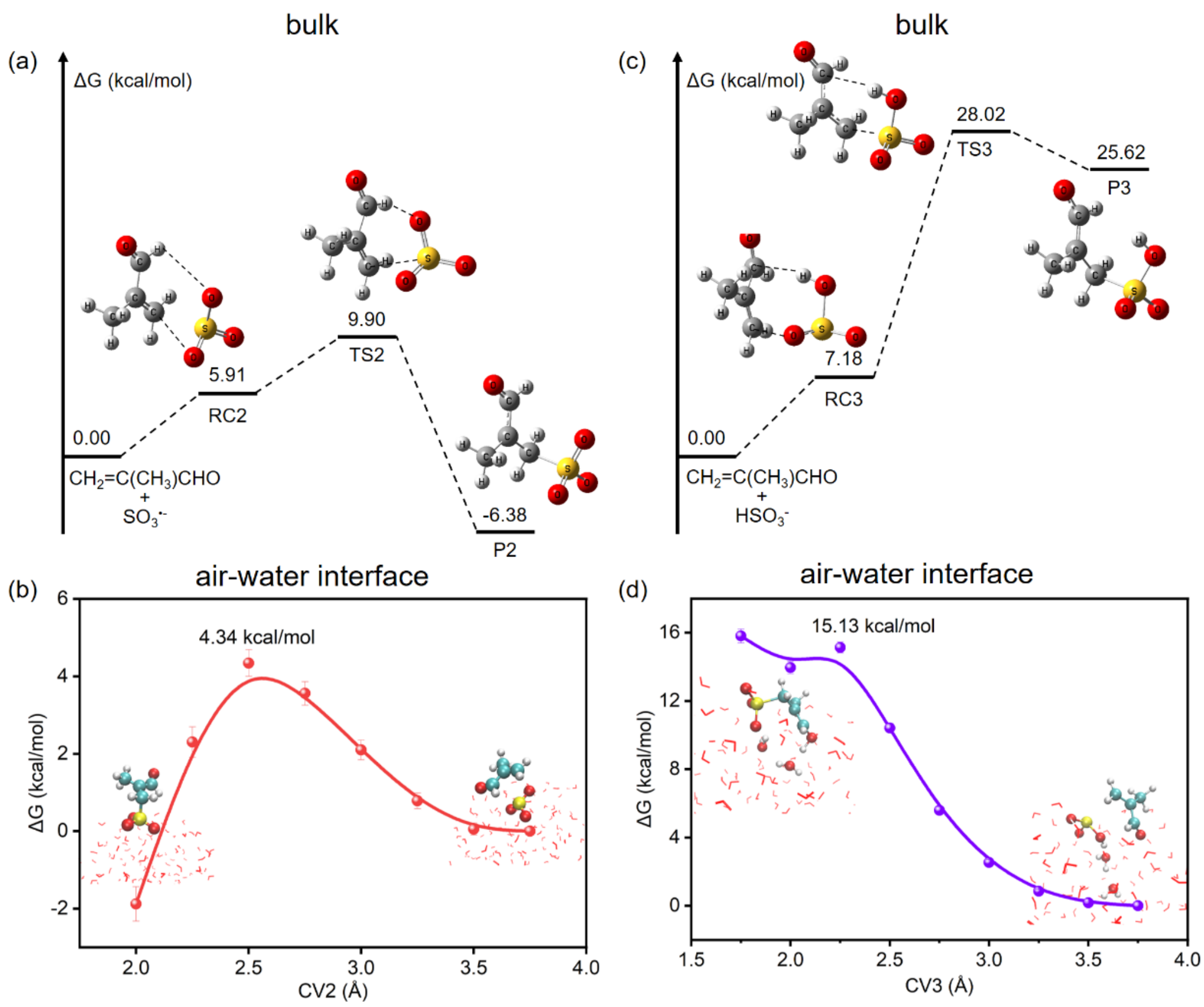

Figure 5. Free energy profiles for the reactions of $SO_3^{\bullet-}$ with $CH_2{=}C(CH_3)CHO$ in the bulk phase (a) and at the air-water interface of microdroplets (b), HSO_3^- with $CH_2{=}C(CH_3)CHO$ in the bulk phase (c) and at the air-water interface of microdroplets (d).

6. Comparison between bulk and microdroplet systems
The comparison with bulk-phase experiments highlights strong differences in reactivity, but the conditions (e.g., concentration, oxidant availability, mixing) are not directly comparable. This limitation should be more explicitly acknowledged when drawing conclusions about rate enhancement.

RE: We thank the reviewer for this important comment. We performed bulk phase control experiments under the same reactant concentrations in the microdroplet experiments and compared with previous study (Huang et al., *Environ. Sci. Technol.* 2020, 54, 11857-11864). Nevertheless, the microdroplet system consistently exhibited higher product yields within shorter reaction times, despite operating at lower reactant concentrations and without the need for transition-metal catalysts. It is worth noting that continuous solvent evaporation and interfacial confinement in microdroplets may dynamically alter local reactant concentrations during flight as reported in previous study (Lee et al., *Proc. Natl. Acad. Sci. U. S. A.* 2015, 112, 3898-3903). Therefore, our comparison with bulk-phase experiments is intended primarily to demonstrate the markedly enhanced reactivity and altered chemistry occurring in microdroplets, rather than to establish a directly comparable intrinsic rate constant. We have clarified this limitation in the revised manuscript.

Page 14, Line 273-281, We have made corresponding modification as follows:

"Bulk phase experiments were conducted for all OVOC systems using the same reactant concentrations as those employed in the microdroplet experiments. After 72 h, trace amounts of organosulfur products were observed only in the Na_2SO_3 and 3-methyl-2-butenal system, whereas no detectable products were observed in the other OVOC systems. Previous studies further show that organosulfur formation in bulk requires substantially higher concentrations (2 mM) and the presence of Fe^{3+}, with reaction times of 12 hours.[10,67] These comparisons suggest that organosulfur formation is significantly enhanced in microdroplets relative to bulk experiments, despite differences in experimental conditions, such as concentration, oxidant availability, and mixing environments."

7. Minor points
a) Descriptive terms such as "rapid", "remarkable", and "spontaneous" are used frequently and could be replaced or supported by quantitative metrics.

RE: We thank this reviewer for pointing this out. We have made corresponding modification in the revised manuscript.

Page 9, Line 162-163, We have revised "rapidly" to "readily" as follows:

"These results demonstrate that HSO_3^- and carbonyl compounds can readily react to form sulfonate in water microdroplets."

Page 11, Line 206-209, We have revised "rapidly" to "on the microsecond timescale" as follows:

"These findings suggest that the C=C bond can serve as a highly reactive site for $SO_3^{-\bullet}$ addition, enabling the spontaneous formation of stable organosulfur products at the air-water interface of microdroplets on the microsecond timescale."

b) Figures, particularly mass spectra, would benefit from clearer annotation and discussion of uncertainty or reproducibility.

RE: We thank the reviewer for this valuable comment. Exact *m/z* values and corresponding species assignments were clearly labeled in all mass spectra. In addition, error bars (mean ± SD, n = 3) in the quantitative plots (Figures S16 and S17) are small, indicating good reproducibility and low measurement uncertainty.

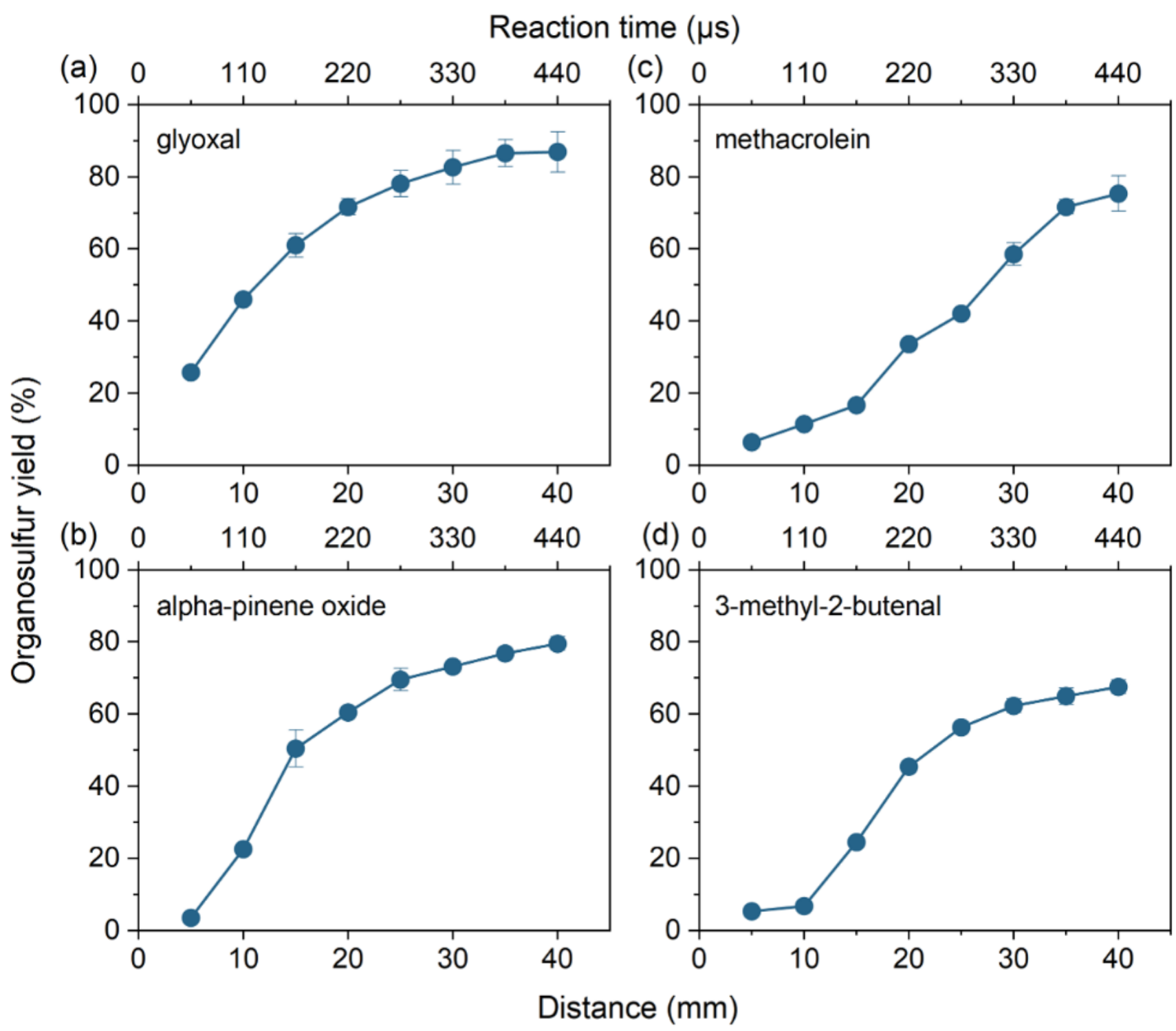

Figure S16. Organosulfur yield as a function of the reaction distance and reaction time.

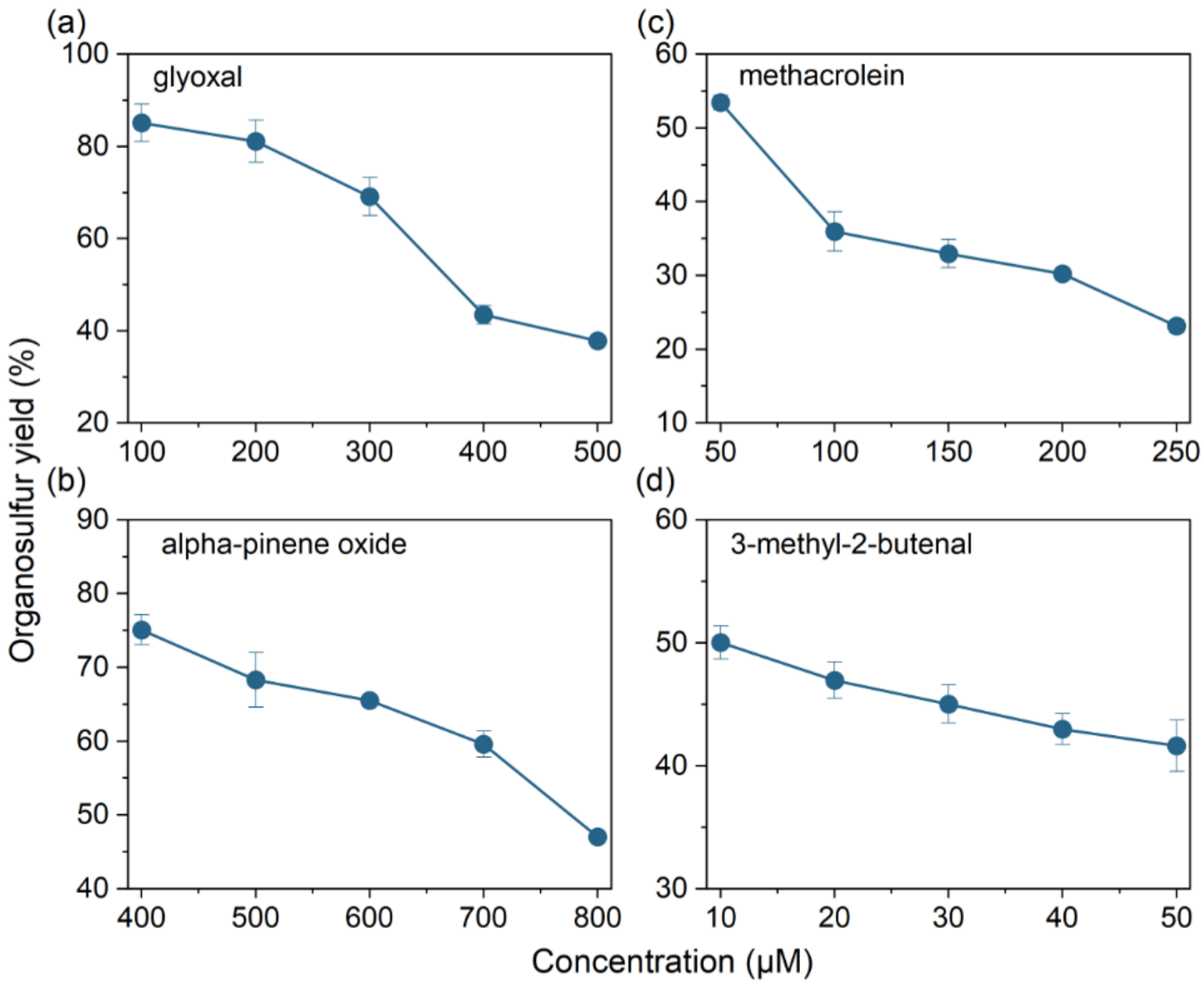

Figure S17. Organosulfur yield as a function of the OVOCs concentration when the reaction distance is 20 mm.

Response to Reviewer #2

In this manuscript, the authors report a novel finding that inorganic sulfur can spontaneously and rapidly convert into organosulfur compounds within atmospheric microdroplets at the air-water interface. The reaction proceeds within approximately 220 microseconds without any catalyst, external potential, or radiation, driven by the strong interfacial electric field of microdroplets. The authors verify the formation pathways via nucleophilic addition and radical addition, and further confirm the existence of these organosulfur products in ambient aerosols from urban Tianjin and high-altitude Shanghuang Station. However, lacking originality in the organosulfur formation pathway, the core reaction types, radical-driven logic, and product assignment belong to extensions of existing mechanisms for atmospheric organosulfur formation (Environ. Sci. Technol. 2025, 59, 16556−16566). No novel reaction mechanism, key intermediate, or driving factor is identified, resulting in limited scientific breakthrough. I suggest this article cannot be published on Nature Communications at present.

RE: We thank the reviewer for the insightful comment. We acknowledge that the elementary reactions involved in this study, such as nucleophilic and radical addition reactions, have been reported previously. However, prior studies have mainly focused on bulk-phase systems, and recent work (*Environ. Sci. Technol*. 2025, 59, 16556-16566) has identified an unexpected gas-phase pathway for organosulfur formation.

In contrast, the novelty of the present work lies in the discovery of a previously unrecognized atmospheric pathway in which inorganic sulfur is rapidly transformed into organosulfur compounds at the air-water interface of microdroplets. This process is driven by interfacial electric fields and occurs on a microsecond timescale without catalysts or external energy input.

Furthermore, theoretical calculations indicate that interfacial electric fields lower the activation barriers and facilitate both radical and nucleophilic reaction pathways, a factor not considered in conventional bulk-phase chemistry. These results highlight the critical role of air-water interface in enabling rapid sulfur conversion and expand the understanding of atmospheric sulfur chemistry at microdroplet interfaces.

Questions:

P6 line 91 & P7 line 120: Laboratory concentrations of Na_2SO_3 (40-100 µM) are used. How do these values compare to ambient sulfite levels in atmospheric droplets, and how can the results be extrapolated to real atmospheric conditions?

RE: We thank the reviewer for this important comment. The concentrations of

Na_2SO_3 used in this study (40-100 μM) fall within the range of S(IV) levels reported in atmospheric aqueous phases, including cloud, fog, and aerosol liquid water (Cheng et al., *Sci. Adv.* 2016, 2, e1601530). Field measurements and thermodynamic calculations have shown that in cloud and fog water, particularly in polluted regions, S(IV) concentrations typically range from tens to hundreds of μM. In aerosol liquid water during haze events, elevated SO_2 levels and high liquid water content can further increase S(IV) concentrations to the sub-mM range. Therefore, the concentration range adopted in this work is atmospherically relevant and suitable for investigating interfacial organosulfur formation pathways under environmentally realistic conditions.

Page 5, Line 89-91, We have made corresponding modification as follows:

"To probe the behavior of SO_3^{2-} at the microdroplet interface, an aqueous solution of Na_2SO_3 (40 μM) was prepared, representative of atmospherically relevant aqueous-phase S(IV) concentrations[19]."

P8–P11 (related to Figure 2): The description of Core MS/MS fragment evidence for key products is too simplistic, and the secondary mass spectrometry data is insufficient. The actual sample also requires corresponding secondary mass spectrometry data, and an explanation is needed as to why only sulfate fragments are present after the cleavage and no fragments from other parts are observed.

RE: We thank the reviewer for this valuable comment. In addition to the sulfur-containing fragment ions, several MS/MS spectra exhibited characteristic neutral losses of 18 Da and 44 Da, which are commonly associated with the loss of H_2O and CO_2, respectively. These diagnostic fragmentation features provide independent structural evidence for the presence of hydroxyl and carboxyl functionalities and further increase confidence in the proposed molecular assignments.

Furthermore, the MS/MS spectra of corresponding ambient aerosol samples have been added (Figure S18). The fragmentation patterns closely match those of the microdroplet generated products, indicating that the organosulfur compounds in atmospheric aerosols are structurally analogous to those formed in microdroplets. The major fragment ion at *m/z* 80.9650 was assigned to HSO_3^-. In negative ion mode, the negative charge is preferentially retained on the sulfonate group, while the organic moiety is readily eliminated as neutral fragments. Consequently, sulfur-containing fragments dominate the MS/MS spectra. This behavior is consistent with previously reported fragmentation pathways of sulfate-containing compounds (Huang et al., *Environ. Sci. Technol. Lett.* 2018, 5, 315-321; Jariwala et al., *J. Mass. Spectrom.* 2012, 47, 529-538).

Page 21, Line 385-395, We have added corresponding discussion as follows:

"To further confirm the structure of the organosulfur compounds detected in ambient aerosols, MS/MS analyses were performed using an LTQ Orbitrap Velos on aerosol sample collected from Tianjin. The MS/MS measurements were carried out in LTQ mode with low resolution because of weak and unstable the product ion signals. As shown in Figure S18, the characteristic fragment ions from the ambient aerosol were consistent with those generated in the microdroplet experiments. In particular, the fragment ion at *m/z* 81 (HSO_3^-) was observed in all organosulfur products generated in microdroplet experiments and ambient samples, indicating the presence of sulfonate functional groups. Together with the agreement in accurate mass measurements from FT-ICR MS, the MS/MS fragmentation patterns provide evidence that the organosulfur compounds formed in microdroplets are structurally analogous to those present in atmospheric aerosols, supporting the atmospheric relevance of the proposed microdroplet formation pathway."

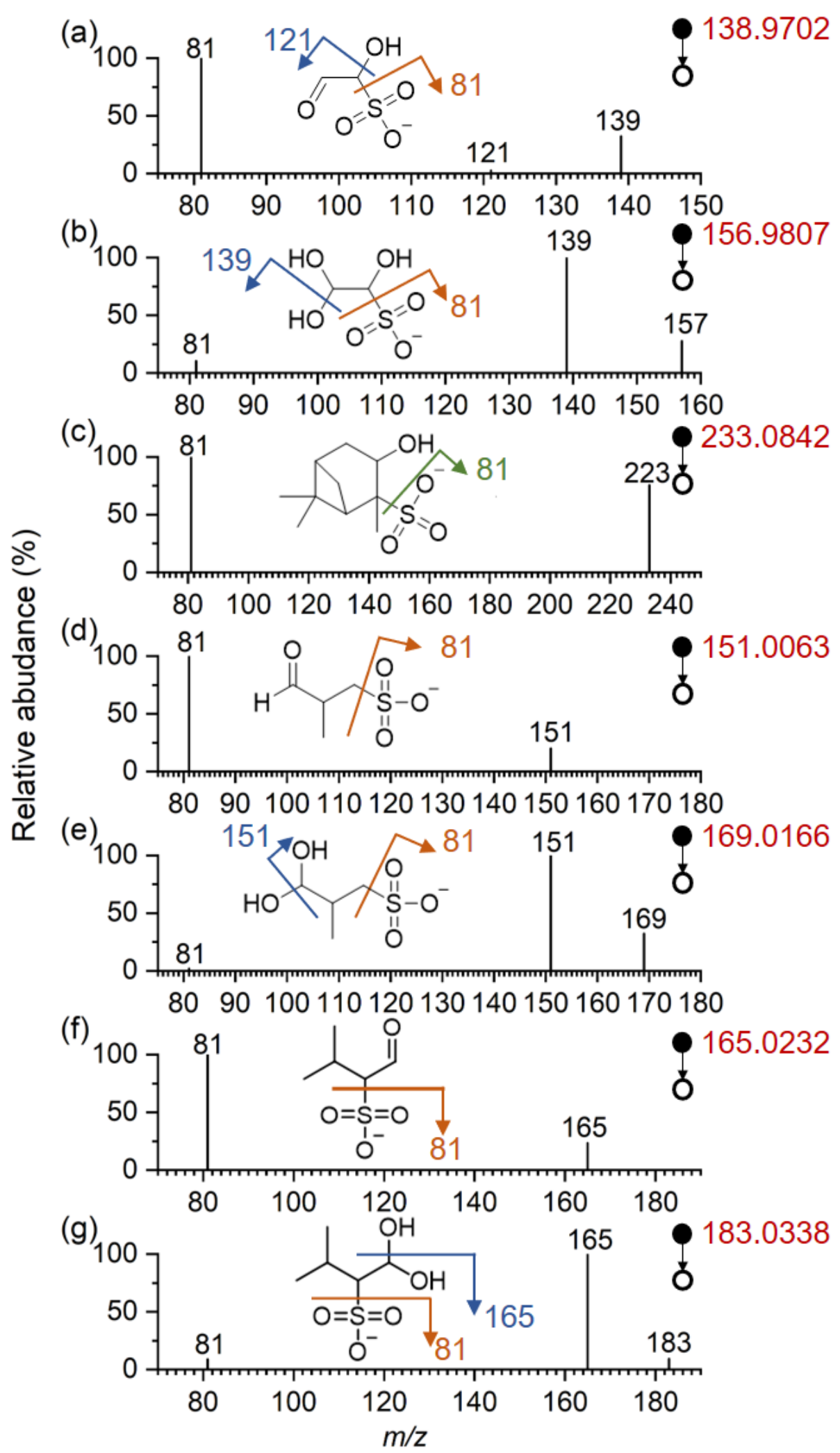

Figure S18. MS/MS spectra of organosulfur compounds detected in ambient aerosol samples collected from Tianjin.

P12 line 207–211: Bulk control experiment only used methacrolein and Na_2SO_3 for 3 days. Were bulk experiments also performed for glyoxal, alpha pinene oxide, and 3 methyl 2 butenal to confirm no organosulfur formation?

RE: We thank the reviewer for this valuable suggestion. Additional bulk experiments were conducted for all OVOC systems using the same reactant concentrations as those employed in microdroplet experiments. After 72 h, only the Na_2SO_3 and 3-methyl-2-butenal system showed trace product, while no detectable products were observed for the other OVOC systems. These results demonstrate that organosulfur formation in bulk solution is either extremely slow or negligible under comparable conditions.

Page 14, Line 273-281, We have made corresponding modification as follows:

"Bulk phase experiments were conducted for all OVOC systems using the same reactant concentrations as those employed in the microdroplet experiments. After 72 h, trace amounts of organosulfur products were observed only in the Na_2SO_3 and 3-methyl-2-butenal system, whereas no detectable products were observed in the other OVOC systems. Previous studies further show that organosulfur formation in bulk requires substantially higher concentrations (2 mM) and the presence of Fe^{3+}, with reaction times of 12 hours.[10,67] These comparisons suggest that organosulfur formation is significantly enhanced in microdroplets relative to bulk experiments, despite differences in experimental conditions, such as concentration, oxidant availability, and mixing environments."

P13 The reaction pathway is determined solely based on mass spectrometry data and speculation. At the very least, theoretical calculations are required to validate the speculation.

RE: We thank the reviewer for this important comment. In revised manuscript, we have performed quantum chemical calculations and metadynamics simulations based on Born-Oppenheimer molecular dynamics (BOMD) to further evaluate the proposed reaction mechanisms. The theoretical results are in good agreement with the experimental observations and provide consistent support for the proposed mechanistic pathways.

Page 17-18, Line 313-336, We have added corresponding discussion as follows:

"To further elucidate these mechanisms, quantum chemical calculations and metadynamics simulations based on Born-Oppenheimer molecular dynamics (BOMD) were performed. Considering that the epoxide moiety of alpha-pinene oxide readily undergoes acid-catalyzed ring opening to form carbonyl-containing intermediates, the theoretical investigations focused on the reactions of HSO_3^- with carbonyl compounds and $SO_3^{-\bullet}$ with unsaturated C=C bonds. The calculations indicate that HSO_3^- reacts with carbonyl compounds through a nucleophilic addition mechanism. The reaction is initiated by C-S bond formation, followed by proton transfer from HSO_3^- to the carbonyl oxygen, mediated by a hydrogen-bonded bridge formed by two interfacial water molecules. The activation free energy at the air-water interface is 11.47 kcal/mol (Figure 4b), which is 7.45 kcal/mol lower than that in bulk solution (Figure 4a), indicating that carbonyl sulfonation is kinetically enhanced in microdroplets. In addition, $SO_3^{-\bullet}$ preferentially adds to the less substituted carbon of the unsaturated C=C bond, with an activation free energy of 4.43 kcal/mol at the air-water interface (Figure 5b), which is 5.47 kcal/mol lower than that in bulk solution (Figure 5a). The reduced reaction energy barrier at the air-water interface is widely attributed to the strong interfacial electric fields in microdroplets.[22,69] In addition, previous studies have demonstrated that external electric fields can further lower reaction barriers and thereby facilitate chemical reactions, highlighting the potential role of interfacial electric fields in modulating microdroplet reactivity.[71,72] In contrast, direct addition of HSO_3^- to the C=C bond exhibits a substantially higher activation barrier (15.13 kcal/ mol) (Figure 5d). These results indicate that alkene sulfonation at the air-water interface proceeds predominantly via an $SO_3^{-\bullet}$-mediated radical pathway rather than direct nucleophilic addition by HSO_3^-. Overall, the theoretical results suggest that interfacial electric fields and interfacial water molecules facilitate the reactions. The combined experimental and computational evidence supports the proposed reaction mechanisms."

Page 25-26, Line 480-504, we have added corresponding method in the revised manuscript in Method Section as follows:

"**Theoretical Methods.** All quantum chemical calculations were performed using the Gaussian 16 software package.[76] Geometry optimizations and frequency calculations were carried out at the M06-2X[77]/6–311++G(d,p) level. Solvent effects were incorporated in all calculations via the SMD continuum solvation model.[78] To confirm the validity of the transition state structures, intrinsic reaction coordinate (IRC) analyses were conducted,[79] verifying that each transition state connects the two designated minima.

Metadynamics simulations based on Born-Oppenheimer molecular dynamics (BOMD) were performed using the CP2K software to investigate the reaction mechanism at the air-water interface.[80] The interfacial model was constructed with a 35 Å cubic box containing 191 water molecules; this box size reproduces the correct water density and prevents spurious long-range interactions between periodic images.[81,82] Prior to the

BOMD simulations, the system was fully relaxed using the Becke-Lee-Yang-Parr functional[83,84] with Grimme's dispersion correction[85,86] to accurately describe weak dispersion interactions. The electronic structure was treated with a double-ζ basis set in conjunction with Goedecker-Teter-Hutter norm-conserving pseudopotentials and the corresponding auxiliary basis set;[87-89] the plane-wave cutoff and the cutoff for the Gaussian basis set were set to 280 and 40 Ry, respectively. All dynamics were propagated in the NVT ensemble at 300 K using a Nosé-Hoover chain thermostat with an integration time step of 1 fs.[90,91]

The free energy of the reaction was computed using the two-step metadynamics coupled with thermodynamic integration (TMTI) strategy.[92] This approach combines metadynamics, multiple-walker metadynamics,[93] and thermodynamic integration[94] in a two-step "pathway + energy" scheme. The reaction path is identified by deploying high and wide Gaussian hills along a one-dimensional collective variable, and the free energy is subsequently refined along the obtained path through parallel multiple-walker sampling or point-by-point thermodynamic integration, enabling efficient and high-fidelity reconstruction of the free energy surface for complex reaction systems."

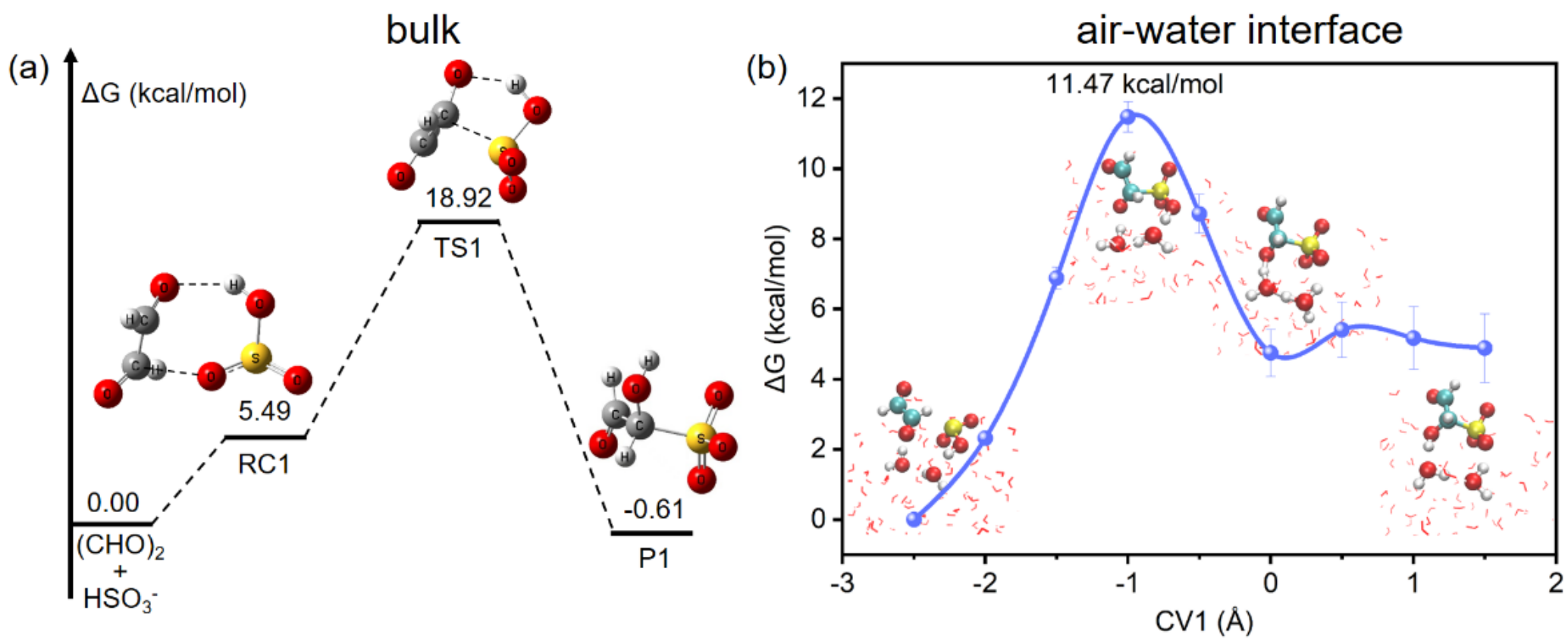

Figure 4. Free energy profiles (ΔG) for the reaction of HSO_3^- with $(CHO)_2$ in the bulk phase (a) and at the air-water interface of microdroplets (b).

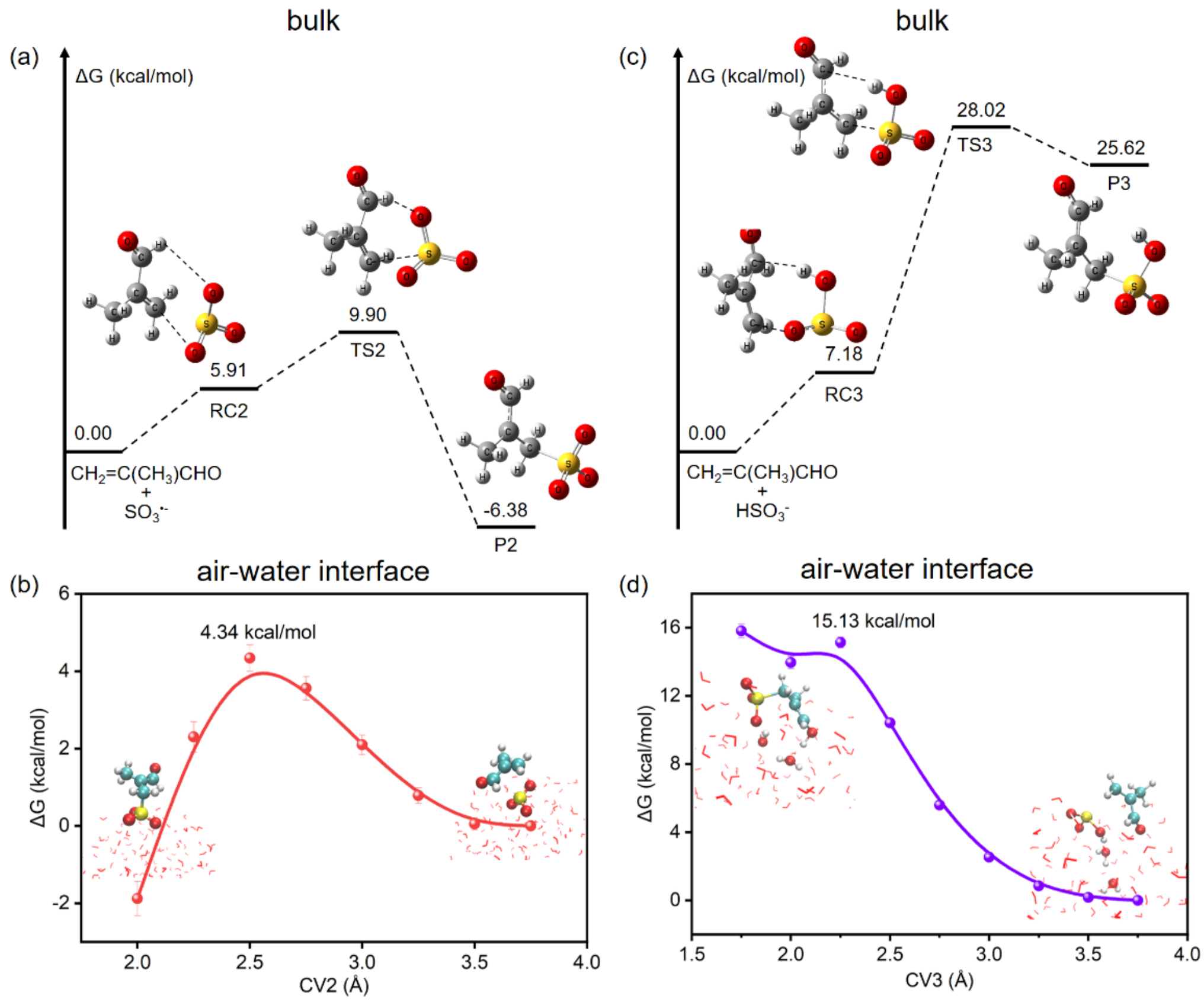

Figure 5. Free energy profiles for the reactions of $SO_3^{\bullet-}$ with $CH_2=C(CH_3)CHO$ in the bulk phase (a) and at the air-water interface of microdroplets (b), HSO_3^- with $CH_2=C(CH_3)CHO$ in the bulk phase (c) and at the air-water interface of microdroplets (d).

P15 line 260–261: Where is the glyoxal product at m/z 138.9702 confirmed in real samples?

RE: We thank the reviewer for this careful observation. The *m/z* 138.9702 was not evaluated in the ambient aerosol samples because it falls below the lower mass limit of our Fourier transform ion cyclotron resonance mass spectrometry (FT-ICR MS) measurements. In this study, FT-ICR MS data were acquired over the mass range of *m/z* 150-1000, which is commonly used for dissolved organic matter (DOM) analyses to ensure high mass accuracy and reliable molecular formula assignments. Consequently, *m/z* 138.9702 could not be assessed in the ambient samples by FT-ICR MS.

P16 line 272–277: The absence of m/z 169.0176 and m/z 183.0333 in Shanghuang samples is attributed to larger droplets and weaker interfacial electric field. Is there field data (droplet size distribution) to quantitatively support this explanation? Why is it not the case that the corresponding volatile organic compound emissions from this

observatory are low? This speculation requires more reasonable verification rather than mere speculation.

RE: We thank the reviewer for this helpful comment. We acknowledge that aerosol size distribution data were not available during the sampling period. Nevertheless, κ-Köhler theory and previous studies in comparable Chinese urban and coastal environments (Wu et al., *Environ. Sci. Technol. Lett.* 2018, 5, 160-166; Chen et al., *Atmos. Chem. Phys.*, 2019, 19, 1327-1342; Li et al., *Atmos. Chem. Phys.* 2025, 25, 3669-3685) have shown that aerosol liquid water content and particle hygroscopic growth increase with relative humidity. During the sampling period, the relative humidity at Shanghuang Station (67-69%) was higher than that at Tianjin (53-59%) (https://www.ncei.noaa.gov/maps/lcd), suggesting the possibility of enhanced aerosol hygroscopic growth and a relatively larger aerosol size distribution at Shanghuang Station. In addition, proton-transfer-reaction mass spectrometry (PTR-MS) measurements from previous studies (Zhang et al., *Sci. Total Environ.* 2024, 919, 170633; Huangfu et al., *Atmos. Chem. Phys.* 2025, 25, 17613-17628) indicate that oxygenated VOCs (OVOCs) concentrations at Shanghuang are lower than in Tianjin.

Therefore, the higher relative humidity at Shanghuang may promote aerosol growth and influence interfacial chemistry, and the lower concentrations of precursor VOCs at this background site may also partly account for the absence of signals at *m/z* 169.0176 and *m/z* 183.0333. Accordingly, we have provided a detailed discussion in revised manuscript (Page 22, Line 372-383).

Page 21, Line 371-383, We have made corresponding modification as follows:

> "Notably, the absence of the peak at *m/z* 169.0176 ($C_4H_9O_5S^-$) and *m/z* 183.0333 ($C_5H_{11}O_5S^-$) in samples collected from Shanghuang Station may be associated with the higher relative humidity (67-69%) at this background site compared with that in Tianjin (53-59%) (https://www.ncei.noaa.gov/maps/lcd). The increased aerosol liquid water content under higher relative humidity could promote particle growth, potentially weakening the interfacial electric field strength of microdroplets due to the decreased curvature,[32,39] thereby reducing the generation of •OH and subsequently suppressing the further oxidation of the *m/z* 151.0070 ($C_4H_7O_4S^-$) and *m/z* 165.0227 ($C_5H_9O_4S^-$) product.[73] In addition, lower OVOC precursor abundance at Shanghuang station[74,75] may contribute to the absence of these products. Moreover, the signal intensity of *m/z* 233.0853 ($C_{10}H_{17}O_4S^-$) is higher in samples from Tianjin than in those from Shanghuang, which may be due to higher concentrations of anthropogenic OVOCs and more favorable interfacial oxidation conditions."

Response to Reviewer #3

The manuscript reports the rapid and spontaneous formation of organosulfur compounds from inorganic sulfite and representative oxygenated volatile organic compounds in aqueous microdroplets. This is an interesting and timely study that addresses an important question in atmospheric chemistry related to the air–water interface of aqueous aerosols. The experimental observations are potentially significant, and the combination of laboratory microdroplet experiments with ambient aerosol measurements strengthens the environmental relevance of the work. Overall, the manuscript is clearly organized, and the results generally support the proposed formation pathways based on the current experimental setup. These findings have significant implications for understanding organosulfur formation in atmospheric aqueous aerosols. I recommend minor revision with the following suggestions for the authors.

RE: We sincerely thank the reviewer for the positive evaluation of our work. We have carefully addressed all your constructive suggestions to further improve the manuscript. The information has been presented in the revised manuscript based on the reviewer's suggestions.

1. The order of the paragraphs in the Introduction could be adjusted to better highlight the importance of this study in the context of atmospheric chemistry.

RE: We thank the reviewer for this insightful suggestion. In revised manuscript, we have reorganized the Introduction to improve its logical flow and better emphasize the atmospheric significance of this study.

Page 3-5, Line 29-86, in the Introduction section, we have made corresponding modification as follows:

"Organosulfur compounds play a crucial role in radiative forcing, global climate regulation and human health owing to their hygroscopicity, optical properties, and toxicity.[1-3] Traditionally known organosulfur formation mechanisms include the reactive uptake of epoxides,[4,5] sulfate esterification reactions,[6,7] heterogeneous reactions of SO_2,[8,9] and radical-initiated processes that typically require transition metal ions (TMI) catalysis (e.g., Fe^{3+}, Mn^{2+}) or additional radiation in bulk aqueous solutions.[10-12] However, estimates of organosulfur concentrations remain subject to large uncertainty.[13] These discrepancies highlight a critical gap in understanding the formation mechanisms of organosulfur under atmospheric conditions.

Field observations consistently show strong correlations between organosulfur and inorganic sulfur species,[2] implying that their transformation is likely coupled in the

atmosphere. In inorganic sulfur chemistry, the oxidation of S(IV) (SO_3^{2-}, HSO_3^-, and/or SO_2•H_2O) to S(VI) (SO_4^{2-}) is a fundamental atmospheric process that underpins sulfate aerosol formation, haze evolution, and acid rain.[14,15] Conventionally, sulfate formation has been primarily attributed to homogeneous oxidation of S(IV), either in the gas phase or in the bulk aqueous phase, through reactions with oxidants such as •OH, H_2O_2, O_3, HONO, or NO_2.[16] Despite extensive studies on the formation mechanisms of sulfate, atmospheric models consistently underestimate sulfate concentrations during haze events, revealing substantial gaps in our understanding of S(IV) oxidation pathways.[17-19] The observed sulfate deficit correlates strongly with aerosol liquid water content,[19] suggesting that heterogeneous or interfacial S(IV) oxidation processes may play a crucial role in polluted atmospheric environments. However, current studies on sulfur oxidation occurring on the interface remain largely focus on inorganic sulfur species.[20-22] In contrast, organosulfur formation has received limited attention and has mainly been investigated in a few specific organosulfur systems, such as dimethyl sulfide, thiols, and thioethers.[23-25] Consequently, the broader conversion of inorganic sulfur into organosulfur compounds remains largely unexplored.

Recently, microdroplet chemistry has gained prominence as a transformative approach due to the fact that chemical reactions within microdroplets may be accelerated by up to 6 orders of magnitude relative to those in bulk water, which can even trigger some reactions that cannot occur in bulk solution.[26-28] This remarkable acceleration has been ascribed to the distinct physicochemical environment of microdroplets, including extreme pH, partial solvation, enrichment and orientation of the reagents, and ultrahigh electric field on the order of 10^9 V m^{-1}.[29-34] The origin of such intense electric field has been attributed to electric double layers, alignment of the interfacial O-H bond, or the partial charge separation yielding H_2O^+-H_2O^- pairs.[32-35] The interfacial electric field strength is sufficiently strong to extract electrons from hydroxide ions (OH^-), generating hydroxyl radicals (•OH) and solvated electrons,[36,37] which can subsequently initiate oxidation and reduction reactions.[38-41] These findings highlight that microdroplets can serve as active microreactors in which unique interfacial physicochemical properties govern reaction dynamics, offering valuable insights into chemical transformations occurring within the ubiquitous atmospheric micrometer-sized aqueous droplets, such as cloud (10 - 20 μm), fog (~ 10 μm), and wet aerosols (0.002 - 10 μm).[42-44]

Given the variable valence states of sulfur and the spontaneous redox capability of microdroplets, it is plausible that inorganic S(IV) could generate reactive sulfur species at the air-water interface of microdroplets and subsequently participate in the formation of organosulfur compounds. In this study, we used sulfite (SO_3^{2-}) and several representative OVOCs (glyoxal, alpha-pinene oxide, methacrolein, and 3-methyl-2-butenal) as substrates to elucidate potential organosulfur compounds formation pathways in water microdroplets. Notably, a variety of organosulfur compounds rapidly formed within hundreds of microseconds and subsequently detected using high-resolution mass spectrometry. Mechanistically, the strong interfacial electric field of water microdroplets drives the oxidation of SO_3^{2-} to $SO_3^{-\bullet}$, which then adds to

unsaturated C=C bonds. Meanwhile, HSO_3^- undergoes nucleophilic addition to carbonyl and epoxide groups. Collectively, these processes enable the interfacial conversion of inorganic sulfur into organosulfur compounds. Quantum chemical calculations and metadynamics simulations were further employed to elucidate the mechanisms of organosulfur formation at the air-water interface of microdroplets. Furthermore, organosulfur products identified in these microdroplet experiments were detected in ambient aerosol samples collected from urban Tianjin and a high-mountain site at the Shanghuang Station in East China using Fourier transform ion cyclotron resonance mass spectrometry (FT-ICR MS). This work provides a new perspective on organosulfur formation in the atmosphere and underscores the importance of aerosol microdroplet interfaces in shaping secondary organic aerosol (SOA) chemistry."

2. For the statement, "In particular, the potential contributions of interfacial or microdroplet-mediated processes to organosulfur formation have rarely been explored," relevant references should be added to better clarify how this work builds upon previous studies.

RE: We thank the reviewer for this suggestion. In revised manuscript, we have added relevant references and expanded the Introduction to better clarify the research background about the potential contributions of interfacial or microdroplet-mediated processes to organosulfur formation.

Page 3, Line 48-52, We have added references and made corresponding modification as follows:

"However, current studies on sulfur oxidation occurring on the interface remain largely focus on inorganic sulfur species.[20-22] In contrast, organosulfur formation has received limited attention and has mainly been investigated in a few specific organosulfur systems, such as dimethyl sulfide, thiols, and thioethers.[23-25] Consequently, the broader conversion of inorganic sulfur into organosulfur compounds remains largely unexplored."

20 Wang, W. et al. Significantly accelerated photosensitized formation of atmospheric sulfate at the air-water interface of microdroplets. J. Am. Chem. Soc. 146, 6580-6590 (2024).
21 Liu, Y. et al. Strong electric field force at the air/water interface drives fast sulfate production in the atmosphere. Chem 10, 330-351 (2024).
22 Li, Y. et al. Epoxidation selectivity of cyclohexene catalyzed by electric field at the interface of microdroplets without catalyst. J. Am. Chem. Soc. 147, 33460-33471 (2025).
23 Du, L. et al. Droplet surface spontaneous oxidation as a dominant formation pathway of organosulfates in the marine atmosphere. Nat. Commun. 16, 10146 (2025).

24 Rao, Z. et al. Spontaneous oxidation of thiols and thioether at the air–water interface of a sea spray microdroplet. J. Am. Chem. Soc. 145, 10839-10846 (2023).
25 Tang, H. et al. Oxidation of organic sulfur at the air-water interface of microdroplets: a new way of atmospheric particle precursor formation. ACS Environ. Au 6, 74-81 (2026).

3. The reaction conditions, such as RH and temperature, as well as solution properties, such as solution pH, should be clearly specified.

RE: We thank the reviewer for this suggestion. We have revised the manuscript to explicitly report the experimental parameters in the Methods section (Page 24, Line 449-452). Specifically, the microdroplet reactions were performed at an ambient temperature of approximately 26 °C and a relative humidity of 47%. Furthermore, the initial pH of all freshly prepared reaction solutions was measured prior to spraying, falling within a near neutral range (pH 6.8-7.6).

Page 24, Line 449-452, in the Methods section (Generation of microdroplets and mass spectrometric analysis), We have made corresponding modification as follows:

"The nebulization and subsequent microdroplet reactions were carried out under ambient laboratory conditions (temperature: ~ 26 °C, relative humidity: ~ 47 %). The freshly prepared mixed solutions of Na_2SO_3 and OVOCs exhibited near-neutral pH values ranging from 6.81 to 7.57 prior to spraying, as measured using a pH meter (PHSJ-6L)."

4. The estimated reaction time of approximately 220 μs is central to the claim of ultrafast organosulfur formation. Since this value is based on previous studies, a brief discussion of the uncertainty and limitations of this estimate would be helpful. If available, reaction-distance-dependent data would further strengthen this claim.

RE: We thank the reviewer for these insightful comments. The estimated reaction time of approximately 220 μs was estimated from the flight distance (20 mm) and droplet velocity (~90 m s^{-1}). The flight distance, defined as the path from the nebulizer outlet to the mass spectrometer inlet, was manually measured and may introduce minor uncertainty. The droplet velocity was adopted from previous micro-PIV studies (Lai et al., *J. Am. Soc. Mass Spectrom.* 2018, 29, 1036-1043) under comparable experimental conditions, as it was not directly measured in this work. We have added a brief discussion in revised manuscript.
In addition, reaction-distance-dependent experiments (5-40 mm, corresponding to 55-495 μs) showed a clear increase in product yield with increasing reaction distance (Figure S16). These results provide experimental support for organosulfur formation during microdroplet flight on the microsecond timescale.

Page 8, Line 138-139, We have added the following discussion:

"These parameters were not directly measured in this work and should therefore be considered approximate."

Page 13, Line 253-260, We have added the following discussion:

"Moreover, semi-quantitative analysis was performed using hydroxymethanesulfonate (HMS) as an internal standard at different reaction distances and OVOC concentrations. By adjusting the reaction distance from 5 to 40 mm, the corresponding reaction time was estimated to range from 55 to 495 μs. A clear increase in product yield was observed with increasing distance (Figure S16). The average yield of these products reached up to 50% when the reaction distance is 20 mm. These results indicate that organosulfur formation occurs during the microsecond timescale flight of microdroplets rather than within the mass spectrometer inlet region, which is consistent with previous studies.[45,65]"

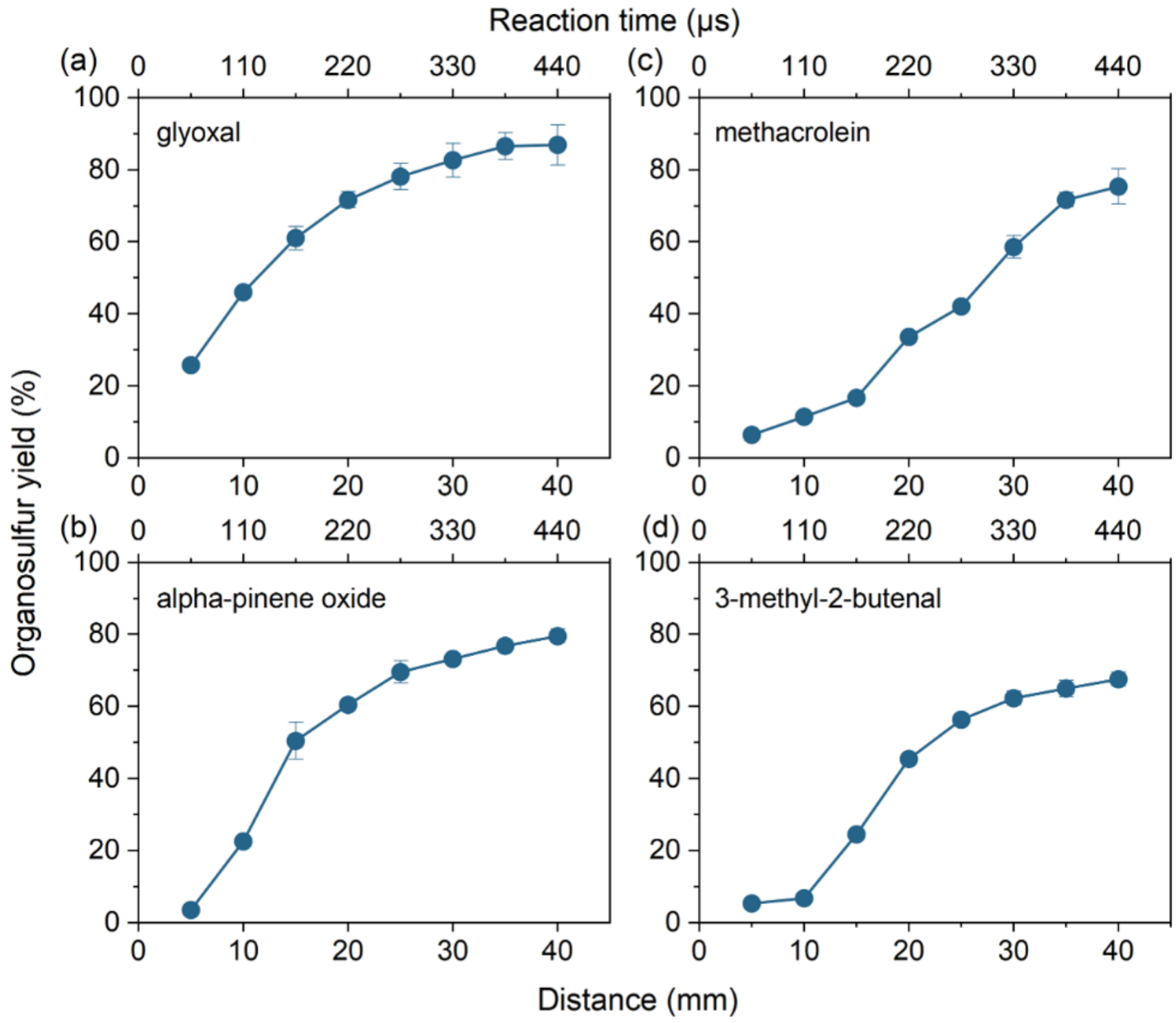

Figure S16. Organosulfur yield as a function of the reaction distance and reaction time.

5. In line 70, the authors propose that inorganic S(IV) can undergo spontaneous oxidation and subsequently react with OVOCs to produce organosulfur compounds. It would be helpful if the authors could present full-scan mass spectra, especially including the low-m/z region, to more clearly demonstrate the formation of sulfate-related species.

RE: We thank the reviewer for this valuable suggestion. In our experiments, the mass spectrometer (LTQ Orbitrap Velos) was operated in full-scan mode over an *m/z* range of 50-1000. The *m/z* 50 is the lowest that can be reliably acquired while maintaining high mass accuracy and resolution on this instrument. This mass range fully covers the low-*m/z* region relevant to inorganic sulfur species and organosulfur products investigated in this study. As shown in the Figure 1b and Figure 2, all key sulfur-containing species are clearly observed, including $SO_3^{-\bullet}$ (*m/z* 79.9573), HSO_3^- (*m/z* 80.9651), $SO_4^{-\bullet}$ (*m/z* 95.9520), and HSO_4^- (*m/z* 96.9600). We regret that this instrumental parameter was not explicitly stated, and it has now been added.

Page 24, Line 440-441, We have added this instrumental parameter in Methods Section:

"Full-scan negative ion mode across a *m/z* range from 50 to 1000 was selected."

6. The manuscript would benefit from a clearer description of the bulk-phase control experiments. In particular, the authors should specify whether similar bulk controls were performed for all OVOC systems. This clarification would strengthen the conclusion that the observed organosulfur formation is specific to the microdroplet environment.

RE: We thank the reviewer for this valuable suggestion. Additional bulk experiments were conducted for all OVOC systems using the same reactant concentrations as those employed in the microdroplet experiments. After 72 h, only the Na_2SO_3 and 3-methyl-2-butenal system showed trace product formation, while no detectable products were observed for the other OVOC systems. These results demonstrate that organosulfur formation in bulk solution is either extremely slow or negligible under comparable conditions, in strong contrast to the rapid product formation observed within microseconds in airborne microdroplets. We have clarified these control experiments and their limitations in the revised manuscript.

Page 14, Line 273-281, We have made corresponding modification as follows:

"Bulk phase experiments were conducted for all OVOC systems using the same reactant concentrations as those employed in the microdroplet experiments. After 72 h, trace amounts of organosulfur products were observed only in the Na_2SO_3 and 3-methyl-2-butenal system, whereas no detectable products were observed in the other OVOC systems. Previous studies further show that organosulfur formation in bulk requires substantially higher concentrations (2 mM) and the presence of Fe^{3+}, with reaction times of 12 hours.[10,67] These comparisons suggest that organosulfur formation is significantly

enhanced in microdroplets relative to bulk experiments, despite differences in experimental conditions, such as concentration, oxidant availability, and mixing environments."

7. For Figure 2, the mechanistic assignments are generally reasonable, but some proposed pathways would benefit from additional discussion or supporting evidence. For example, regarding the formation of $C_2H_5O_6S^-$, $C_4H_9O_5S^-$, and $C_5H_{11}O_5S^-$, it would be helpful to clarify whether the current evidence can distinguish •OH-initiated reactions at the air-water interface from possible hydrolysis-related pathways. If not, the proposed mechanisms should be described more cautiously.

RE: We sincerely thank the reviewer for this insightful suggestion. To evaluate the contribution of •OH-initiated reactions, we performed supplementary experiments using tert-butanol (TBA) as a specific •OH scavenger. The addition of TBA resulted in a substantial decrease in the proposed oxidation products (Figure S12), including $C_2H_5O_6S^-$, $C_4H_9O_5S^-$, and $C_5H_{11}O_5S^-$, suggesting that •OH-mediated oxidation plays an important role in their formation. Nevertheless, the possible contribution of hydrolysis-related pathways cannot be ruled out. Accordingly, we have discussed proposed mechanisms in revised manuscript.

Page 12, Line 222-228, We have added corresponding discussion as follows:

"To further evaluate the role of •OH in organosulfur formation, tert-butanol (TBA) was used as a •OH scavenger.[62] As shown in Figure S12, the addition of 50 μM TBA led to substantial decreases in the intensity ratios of $C_2H_5O_6S^-/C_2H_3O_5S^-$, $C_4H_9O_5S^-/C_4H_7O_4S^-$, and $C_5H_{11}O_5S^-/C_5H_9O_4S^-$, which are derived from the reactions of Na_2SO_3 with glyoxal, methacrolein, and 3-methyl-2-butenal, respectively. These results indicate that •OH contributes to the formation of organosulfur products in water microdroplets. Notably, $C_2H_5O_6S^-$, $C_4H_9O_5S^-$, and $C_5H_{11}O_5S^-$ may also be produced through hydrolysis-related pathways.

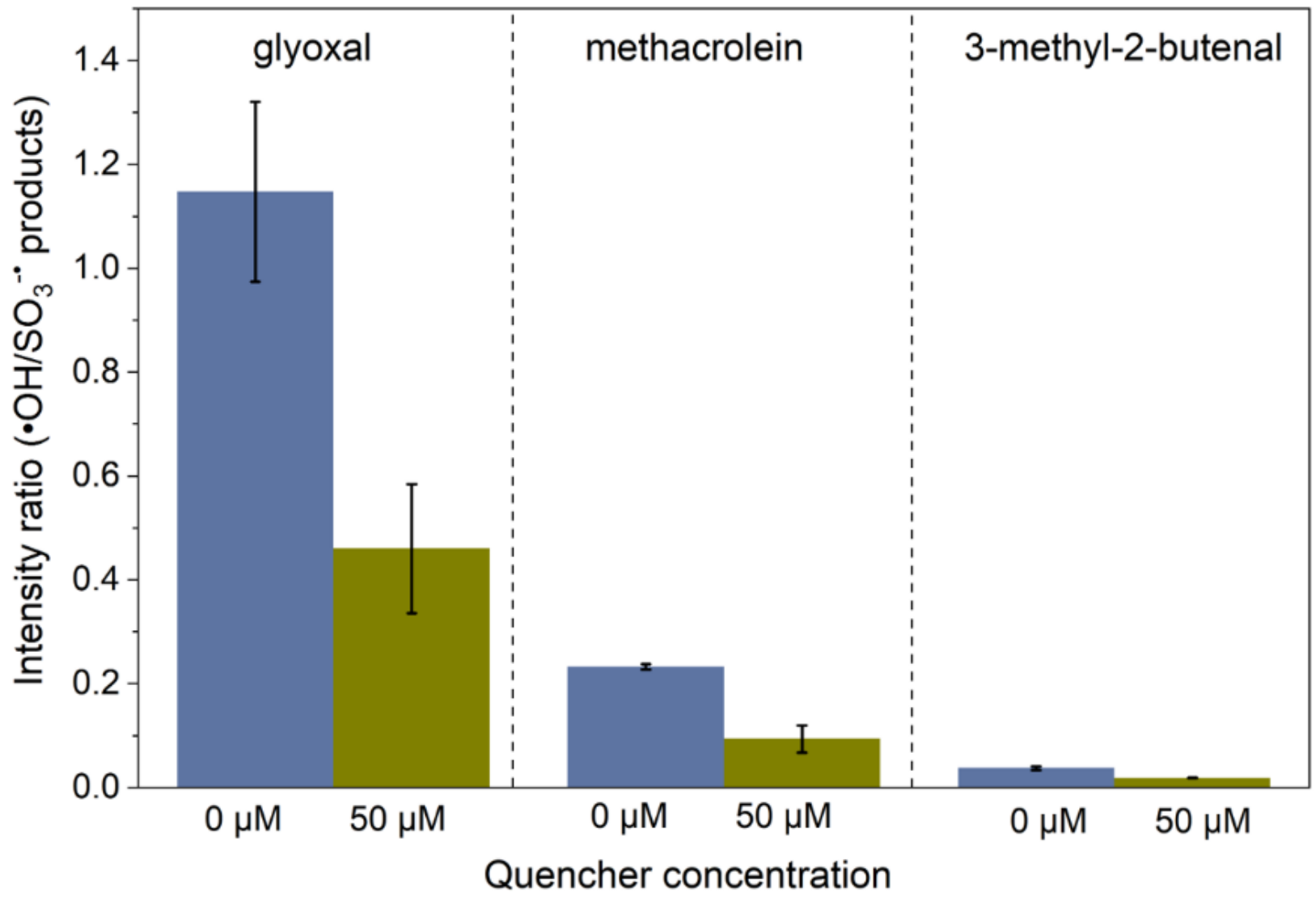

Figure S12. The intensity ratio of organosulfur products, $C_2H_5O_6S^-/C_2H_3O_5S^-$, $C_4H_9O_5S^-/C_4H_7O_4S^-$, and $C_5H_{11}O_5S^-/C_5H_9O_4S^-$, generated from the reactions of Na_2SO_3 with glyoxal, methacrolein, and 3-methyl-2-butenal, respectively, in the absence and presence of TBA.

8. The discussion of the differences between the Tianjin and Shanghuang aerosol samples is interesting. The authors attribute these differences to larger droplet sizes under typically higher relative humidity conditions. Relevant observational data from these two sites would further support this explanation. Otherwise, I suggest that the authors use more cautious wording.

RE: We thank the reviewer for this helpful comment. During the sampling periods, the relative humidity (RH) at Shanghuang Station (67-69%) was higher than that in Tianjin (53-59%) (https://www.ncei.noaa.gov/maps/lcd). According to established κ-Köhler theory and empirical studies in similar Chinese urban and coastal environments (Wu et al., *Environ. Sci. Technol. Lett.* 2018, 5, 160-166; Chen et al., *Atmos. Chem. Phys.* 2019, 19, 1327-1342), the increase in RH would result in larger particles with greater aerosol liquid water content. We have added relevant observational data in revised manuscript.

Page 21, Line 371-383, We have made corresponding modification as follows:

"Notably, the absence of the peak at *m/z* 169.0176 ($C_4H_9O_5S^-$) and *m/z* 183.0333 ($C_5H_{11}O_5S^-$) in samples collected from Shanghuang Station may be associated with the higher relative humidity (67-69%) at this background site compared with that in Tianjin (53-59%) (https://www.ncei.noaa.gov/maps/lcd). The increased aerosol liquid water content under higher relative humidity could promote particle growth, potentially weakening the interfacial electric field strength of microdroplets due to the decreased

curvature,[32,39] thereby reducing the generation of •OH and subsequently suppressing the further oxidation of the *m/z* 151.0070 ($C_4H_7O_4S^-$) and *m/z* 165.0227 ($C_5H_9O_4S^-$) product.[73] In addition, lower OVOC precursor abundance at Shanghuang station[74,75] may contribute to the absence of these products. Moreover, the signal intensity of *m/z* 233.0853 ($C_{10}H_{17}O_4S^-$) is higher in samples from Tianjin than in those from Shanghuang, which may be due to higher concentrations of anthropogenic OVOCs and more favorable interfacial oxidation conditions."

The end.

Responses to Reviewer

We greatly appreciate the positive comments and the insightful suggestions/comments of the referees about our work. We tried our best to revise and improve the manuscript and made changes in the manuscript according to the reviewers' comments. Our point-by-point responses to the comments are presented below, with the reviewers' comments in italics, our responses in regular text, and the corresponding modification made in the manuscript and Supplementary Information are highlighted in blue.

Reviewer #1:

no further comments

RE: We sincerely appreciate the reviewer's positive evaluation.

Reviewer #2:

The authors are commended for their extensive efforts in addressing the prior reviewer comments, including the addition of substantial experimental data, theoretical calculations, and targeted textual revisions. The newly added bulk-phase control experiments covering all OVOC systems, complementary ATR-FTIR and UPLC-MS characterizations, quantum chemical calculations and metadynamics simulations, as well as MS/MS verification of ambient aerosol samples, have markedly improved the comprehensiveness and evidentiary strength of the work. However, several issues remain to be clarified.

RE: We are grateful to this reviewer's positive comments. We have carefully addressed all your constructive suggestions to further improve the manuscript. The information has been presented in the revised manuscript based on the reviewer's suggestions.

1. The supplementary sheath gas pressure-dependent experiment only establishes a positive correlation between decreased droplet size and elevated $SO_3^{\bullet-}$ signal intensity. Critically, droplet size reduction is inherently accompanied by concentration effects driven by solvent evaporation, as well as enrichment of reactants at the air-water interface. The proposed mechanism requires more targeted verification, or discussion.

RE: We thank the reviewer for this insightful comment. We agree that increasing the sheath gas pressure decreases droplet size, which is inherently accompanied by concentration effects arising from solvent evaporation and enrichment of reactants at the air-water interface. Therefore, the increased $SO_3^{\bullet-}$ signal intensity likely reflects the combined effects of these factors. We have revised the manuscript and added further discussion to clarify the proposed mechanism.

Page 6-7, Line 116-123, We have added the corresponding discussion:

"As shown in Figure S2, the $SO_3^{-\bullet}$ signal intensity increased with increasing sheath gas pressure. This trend is consistent with a contribution from enhanced interfacial electric field in smaller microdroplets, which may facilitate electron transfer processes involved in $SO_3^{-\bullet}$ generation. Nevertheless, enhanced $SO_3^{-\bullet}$ abundance may arise from the combined effects of the interfacial electric field and other physicochemical characteristics inherent to microdroplet environments, including evaporation-driven concentration effects, reactant enrichment, altered solvation environments, and nonequilibrium interfacial dynamics.[45,46]"

2. From a fundamental chemical perspective, free radical reactions are intrinsically

fast processes. There is no need to explicitly emphasize that the reaction occurs on the microsecond timescale in certain statements, and the wording can be adjusted accordingly (e.g., Page 11, Line 208). Meanwhile, the authors should discuss the half-life of the relevant radical in both the bulk phase and gas phase. If the radical has an extremely short half-life, the reaction may indeed proceed within microseconds, which is consistent with the low ion intensity of the radical detected by mass spectrometry. Additional discussion on this point is needed to ensure the chemical plausibility of the proposed reaction mechanism.

RE: We thank the reviewer for this constructive suggestion. We have added discussion on the transient nature and lifetime of $SO_3^{\bullet-}$. In aqueous environments, $SO_3^{\bullet-}$ is a highly transient intermediate undergoing second-order self-decay with an effective lifetime in the sub-millisecond regime, whereas its gas phase lifetime remains undefined because this radical anion requires solvation stabilization. The transient nature of $SO_3^{\bullet-}$ is consistent with its low MS signal intensity and its rapid involvement in subsequent reactions. We have also revised the relevant statements to clarify that the microsecond residence time of microdroplets is compatible with rapid radical-mediated transformations. These considerations further support the chemical plausibility of the proposed radical-mediated reaction mechanism.

Page 10, Line 198-205, We have added the following discussion:

"These findings suggest that the C=C bond can serve as a highly reactive site for $SO_3^{-\bullet}$ addition, enabling the spontaneous formation of stable organosulfur products at the air-water interface of microdroplets. Notably, in aqueous environments, $SO_3^{-\bullet}$ is a highly transient intermediate with an effective lifetime in the sub-millisecond regime,[64,65] whereas its gas phase lifetime remains undefined because solvation is required for stabilization.[66] The transient nature of $SO_3^{-\bullet}$ is consistent with its low MS signal intensity and rapid involvement in subsequent reactions, with the microsecond residence time of microdroplets providing a temporal window compatible with these radical-mediated transformations."

Overall, the authors have completed a substantial body of supplementary work in this round of revision, which has significantly strengthened the foundational evidence of the study. I recommend acceptance of the manuscript following minor revisions.

Reviewer #3 *(Remarks to the Author):*

The authors had revised their manuscript carefully, now it is acceptable as it is.

RE: We sincerely thank the reviewer for the positive assessment.
